# Comparative Transcriptomics Provides Insights into Reticulate and Adaptive Evolution of a Butterfly Radiation

**DOI:** 10.1093/gbe/evz202

**Published:** 2019-09-13

**Authors:** Wei Zhang, Brian X Leon-Ricardo, Bas van Schooten, Steven M Van Belleghem, Brian A Counterman, W Owen McMillan, Marcus R Kronforst, Riccardo Papa

**Affiliations:** 1 State Key Laboratory of Protein and Plant Gene Research, Peking-Tsinghua Center for Life Sciences, and School of Life Sciences, Peking University, Beijing, China; 2 Department of Ecology and Evolution, University of Chicago; 3 Department of Biology, University of Puerto Rico; 4 Molecular Sciences and Research Center, University of Puerto Rico; 5 Department of Biological Sciences, Mississippi State University; 6 Smithsonian Tropical Research Institute, Gamboa, Panama

**Keywords:** transcriptome, positive selection, adaptation, hybrid speciation, *Heliconius*

## Abstract

Butterfly eyes are complex organs that are composed of a diversity of proteins and they play a central role in visual signaling and ultimately, speciation, and adaptation. Here, we utilized the whole eye transcriptome to obtain a more holistic view of the evolution of the butterfly eye while accounting for speciation events that co-occur with ancient hybridization. We sequenced and assembled transcriptomes from adult female eyes of eight species representing all major clades of the *Heliconius* genus and an additional outgroup species, *Dryas iulia*. We identified 4,042 orthologous genes shared across all transcriptome data sets and constructed a transcriptome-wide phylogeny, which revealed topological discordance with the mitochondrial phylogenetic tree in the *Heliconius* pupal mating clade. We then estimated introgression among lineages using additional genome data and found evidence for ancient hybridization leading to the common ancestor of *Heliconius hortense* and *Heliconius clysonymus*. We estimated the *K*_a_/*K*_s_ ratio for each orthologous cluster and performed further tests to demonstrate genes showing evidence of adaptive protein evolution. Furthermore, we characterized patterns of expression for a subset of these positively selected orthologs using qRT-PCR. Taken together, we identified candidate eye genes that show signatures of adaptive molecular evolution and provide evidence of their expression divergence between species, tissues, and sexes. Our results demonstrate: 1) greater evolutionary changes in younger *Heliconius* lineages, that is, more positively selected genes in the *cydno*–*melpomene*–*hecale* group as opposed to the *sara*–*hortense*–*erato* group, and 2) suggest an ancient hybridization leading to speciation among *Heliconius* pupal-mating species.

## Introduction

Neotropical *Heliconius* butterflies display highly variable wing patterns and serve as a striking example of adaptive radiation (Glor 2010; Heliconius Genome Consortium 2012). *Heliconius* butterflies are chemically defended and their bright color patterns warn predators of this toxicity (Speed 2000; Dell’aglio et al. 2016). To further enhance the warning effect and train predator avoidance learning, distantly related *Heliconius* species have evolved similar wing patterns to form Müllerian mimicry rings (Sheppard et al. 1985; Joron et al. 2006). In addition to advertising their toxicity to predators, *Heliconius* wing patterns also serve as mating cues, with many species and subspecies mating assortatively based on these visual signals (McMillan et al. 1997; Jiggins et al. 2001). However, many members of different lineages still hybridize occasionally (Mallet 2005, 2009), allowing introgressive hybridization to contribute to convergent wing pattern evolution between closely related and even distantly related mimicry pairs (Heliconius Genome Consortium 2012; Martin et al. 2013; Zhang et al. 2016). Such natural hybridization events have also been proven to lead to the origin of hybrid species, such as *Heliconiusheurippa*, which display recombinant color patterns (Mavárez et al. 2006; Jiggins et al. 2008).

In addition to wing patterns, color vision is of particular importance in mate choice, speciation, and adaptation (Oliver et al. 2009; Allen et al. 2011; Bybee et al. 2012). For example, *Heliconius* butterflies have evolved a duplicated ultraviolet opsin protein UVRh2 along with a novel yellow wing pigment, 3-hydroxy-dl-kynurenine, which provides direct evidence of correlated evolution between color vision and wing coloration (Briscoe et al. 2010; Bybee et al. 2012). Moreover, butterfly eyes are extremely complex sensory organs and diverse in both anatomy and physiology (Briscoe and Chittka 2001; Stavenga and Arikawa 2006), which differ significantly among species or even between sexes (Arikawa et al. 2005; Briscoe 2008; Ogawa et al. 2013; McCulloch et al. 2016, 2017). In *Pieris rapae*, the male photoreceptor displays different blue spectral sensitivity for discrimination between male and female wing colors (Arikawa et al. 2005), whereas, in *Heliconius erato*, female butterflies express both UVRh1 (suppressed in males) and UVRh2 for male conspecific discrimination (McCulloch et al. 2016).

Several studies have investigated the patterns of molecular evolution of known vision genes across nymphalid butterflies and linked the duplication and evolution of *opsin* molecules to key adaptations in butterfly visible spectrum (Frentiu, Bernard, Sison-Mangus, et al. 2007). In *Limenitis* butterflies, a long-wavelength (L)-sensitive photopigment, *L opsin* gene, has been shown to contain amino acid sites under positive selection (Frentiu, Bernard, Cuevas, et al. 2007). Similarly, in *Heliconius* butterflies, the adaptive evolution of *UV opsin* genes involves positive selection and gene duplication (Briscoe et al. 2010; Yuan et al. 2010). It is worth pointing out that while most of these studies on a specific group of visual genes have increased our understanding of color perception, only a few of them have addressed the adaptive evolutionary signature of an entire butterfly eye (Catalan, Höhna, et al. 2019; Catalan, Macias-Munoz, et al. 2018; Macias-Munoz et al. 2019).

A butterfly’s eye is characterized by the interplay of photopigments and other proteins that all together send input to the brain (Briscoe 2008). This synergy between photoreceptor cells and other accessory cells suggests that more molecules rather than just the *opsin* genes are driving the evolution of butterflies’ vision. In this study, we generated and analyzed de novo assembled *Heliconius* eye transcriptomes for a total of eight species in order to detect genes subject to positive selection and begin to understand a catalog of expressed genes that might play an important role in the visual property of this highly diverse group of Neotropical butterflies. In addition, by making full use of our transcriptome data and taking advantage of the growing genome resequencing data, we identified phylogenetic incongruence in the *Heliconius* pupal mating clade and depict a case of genome mosaicism in *Heliconiushortense* and *Heliconiusclysonymus*. Our data suggest a possible ancient hybridization event leading to speciation of these two species before their parapatric divergence in Central America. Overall, our study portrays a transcriptome-wide view of the adaptive evolution in butterfly eyes and teases apart relationships among distantly related butterfly species.

## Materials and Methods

### RNA Sampling, Sequencing, and De Novo Transcriptome Assembly

Butterflies eye tissues were isolated from eight newly emerged adult female samples, one of each species: *H. hortense*, *Heliconiuscydno*, *Heliconiussara*, *Heliconiusdoris*, *Heliconiushecale*, *H. erato*, *Heliconiusmelpomene*, and *D. iulia*. RNA was extracted with Trizol followed by the RNeasy minikit from Qiagen and then Illumina paired-end libraries were constructed using the Illumina Truseq protocol and sequenced with an Illumina HiSeq 2000. Raw reads were demultiplexed according to their barcodes and low-quality reads were filtered out before assembly. De novo transcriptome assembly was performed and likely coding sequences were extracted using Trinity version 2013-11-10 (Grabherr et al. 2011; Haas et al. 2013) with default parameters (supplementary table S1, Supplementary Material online).

### DNA Sequencing Data Collection and Genotyping Calling

We downloaded 34 individual genome resequencing data sets from NCBI PRJNA324415 (Van Belleghem et al. 2017) and PRJNA308754 (Zhang et al. 2016). This data set covered species in the pupal mating clade used for transcriptome analyses and also introduced additional species to facilitate characterization of potential hybrid speciation. For example, this data set included additional species in the *sara*–*sapho* clade, subspecies of *H. erato* and their closely related species, a parapatric sister species (*H. clysonymus*) and a closely related species (*Heliconiustelesiphe*) for *H. hortense*. We performed quality control for raw reads using Trimmomatic v0.36 (Bolger et al. 2014) and aligned qualified reads to the *H. melpomene* v2.0 (Davey et al. 2016) using Bowtie2 v2.2.3 (Langmead and Salzberg 2012) with parameter –very-sensitive-local. We used Picard v1.96 (https://broadinstitute.github.io/picard/; last accessed September 18, 2019) to reorder alignments and removed PCR duplicates. We realigned Indels using RealignerTargetCreator and Indelrealigner in GATK v3.7 (McKenna et al. 2010) and called genotypes across 30 individuals using UnifiedGenotyper in GATK v3.7 (DePristo et al. 2011) using the following parameters: heterozygosity 0.01, stand_call_conf 50.0, stand_emit_conf 10.0, and dcov 250 (supplementary table S2, Supplementary Material online).

### Transcriptome-Based Clustering Conserved Coding Sequences

We identified and determined conserved orthologs as reciprocal best hits existing in all the eight species using Blat (Kent 2002). The predicted CDS regions were extracted from the longest isoforms and CDS regions of each species were used as queries and targets, respectively, to search against data sets of all the other seven species. We determined reciprocal best hits with *E* values <10^−6^. With this method, we retrieved the corresponding conserved orthologous sequences that were not necessarily single-copy. All the orthologous clusters were annotated and gene ontology terms were assigned using Blast2GO (Conesa et al. 2005). Conserved mitochondrial genes were extracted using Blat by searching assembled transcriptomes against predicted genes in the mitochondrial genome of *Bombyx mandarina* (GenBank: AY301620.2) (Pan et al. 2008). For each species, “ortholog hit ratio” was calculated by comparing the hit length of each clustered CDS region to the length of the best matched gene in *H. melpomene* (Heliconius Genome Consortium 2012) or *Bombyxmori* (Xia et al. 2004) using BlastX.

### Transcriptome-Based Multiple Alignments and Phylogenetic Reconstruction

Sequences in each conserved cluster were aligned using MACSE (Ranwez et al. 2011) with default parameters. Then aligned clustered sequences were concatenated in order and converted into phylip format. A transcriptome-wide maximum-likelihood phylogenetic tree was constructed using PhyML 3.1 with GTR model and 100 bootstrap replicates (Guindon et al. 2010) whereas a mitochondrial phylogeny was constructed in the same way using aligned concatenated mitochondrial genes. In order to address incongruence between transcriptome-wide and mitochondrial phylogenies, we inferred the best tree topology for each concatenated data set. Phylogenetic trees were generated under specific topological constraints and the two constrained trees were defined as (*D. iulia*, ((*H. erato*, (*H. sara*, *H. hortense*)), (*H. doris*, (*H. hecale*, (*H. cydno*, *H. melpomene*))))) and (*D. iulia*, ((*H. sara*, (*H. hortense*, *H. erato*)), (*H. doris*, (*H. hecale*, (*H. cydno*, *H. melpomene*)))). CONSEL 0.20 49 was used to select the most confident topological structure for each data set with the Shimodaira and Hasegawa test *P* value >0.95.

### Genome-Wide Phylogeny Construction and Divergence Estimation

We extracted SNP calls with good quality (∼36.07 million SNPs per individual with Qual >30) for 17 individuals representing 17 separate species and aligned them to generate a PHYLIP file. We constructed a genome-wide maximum-likelihood phylogeny using RAxML with the GTRGAMMA model and 100 bootstrap replicates (Stamatakis 2006). The output phylogeny file was visualized using iTOL (Letunic and Bork 2016). We estimated divergence times for a tree topology including 11 taxa using PhyTime and calibrated with the mean split time estimates between *Heliconiushecalesia* and *H. erato* (∼4.5 Ma) previously estimated by Cuthill and Charleston (2012).

### Calculating *K*_a_/*K*_s_ Ratios for Orthologous Clusters

We calculated the ratio of nonsynonymous substitution rate (*K*_a_) to synonymous substitution rate (*K*_s_) for each conserved cluster of every species pair using the kaks function (Li 1993) of the seqinr package 3.1-3 (Charif and Lobry 2007). Clusters with *K*_a_/*K*_s_ ratios >1 were checked manually to remove false positive results due to poor alignment. The functional enrichment of clusters subject to positive selection was performed using Blast2GO. We used Fisher’s exact tests for significant enrichment and presented uncorrected *P* values instead of calculating a false-discovery rate (FDR), because it may be too stringent to apply FDR for an initial survey (Huang da et al. 2009).

### Phylogenetic Tests of Positive Selection

The branch-site model implemented in the CodeML program from the package PAML (Zhang et al. 2005) was used to further identify lineages and internal nodes under positive selection from orthologous clusters with *K*_a_/*K*_s_ ratios >1. The branch-site model evaluates selective pressure by comparing an estimated model against a null model. Eight species branches and four internal nodes, (*H. sara* and *H. hortense*), (*H. erato*, *H. sara*, and *H. hortense*), (*H. cydno* and *H. melpomene*), and (*H. hecale*, *H. cydno*, and *H. melpomene*) were selected as foreground branches separately, whereas all other branches were treated as background branches. We used the Benjamini–Hochberg false discovery rate (FDR) of 0.01 to control for multiple testing, which yielded an adjusted *P *<* *0.006023 (Benjamini and Hochberg 1995). The FDR correction only yielded 57 of 99 candidates and greatly masked the selection patterns of internal nodes, so we did not apply it to the actual data set due to its stringency (Huang da et al. 2009). Thus, we retrieved sites under positive selection from significant candidate clusters using Bayes Empirical Bayes method in PAML. Given that the branch-site test makes stringent assumptions about the selective pressures on the branches (Yoshida et al. 2011), we also tested robustness of the branch-site method by performing additional site model tests by comparing an estimated model against a null model including ω estimations among sites, that is, comparing models M8 against M7 and compare models M8 against M8a implemented in the CodeML program (Yang 2007). These joint tests allow us to determine how strong the evidence for positive selection is. The GA-branch method implemented in Hyphy package (Pond et al. 2005) and Datamonkey webserver (Delport et al. 2010) was also used to characterize selection pressure by determining the “best-fitting” model automatically for the putative candidate clusters with *K*_a_/*K*_s_ ratios >1. The functional enrichments of positively selected clusters yielded from the two tests were performed using Blast2GO and Fisher’s exact tests as well.

### qRT-PCR

We examined the expression patterns of four candidate genes in males and females in the eight species. These candidate genes were selected according to the following criteria: 1) orthologs that have shown to be expressed and detected in head tissue in *Drosophila melanogaster*, 2) orthologs suitable for qRT-PCR primer design across all eight species. Three newly emerged (<30 h) females and three newly emerged (<30 h) males of each species were collected and the eye, brain, and body tissues were isolated separately. Total RNA was extracted according to a standard Trizol protocol and cDNA libraries were generated using the ABI’s high-capacity cDNA reverse transcription kit. Species-specific qRT-PCR primers were designed as shown in supplementary table S3, Supplementary Material online and qRT-PCR reactions were run with three experimental replicates and analyzed on a Bio-Rad real-time CFX96 system using ABI’s SYBR green PCR master mix. A reference gene *ef1-α* was selected and analyzed for data normalization.

### *D*-Statistics for Transcriptome and Genome Data

We calculated Patterson’s *D*-statistic (Green et al. 2010; Durand et al. 2011) to characterize gene flow among the potential parental species and hybrid species. The *D*-statistic examines the distribution of derived alleles at loci supporting either an ABBA or BABA topological pattern on a four taxa phylogeny. The number of ABBA or BABA sites should be roughly equal under incomplete lineage sorting. To reject this null hypothesis of gene flow, an excess number of ABBA or BABA sites is expected. For the conserved clusters yielded from transcriptome data, we extracted SNPs from each cluster using ape-package v4.0 (Paradis et al. 2004) and calculated the total number of derived SNPs supporting either an ABBA or BABA pattern for each cluster using the original equation of *D*-statistic (Durand et al. 2011):

(1) $$D\left(P_{1},P_{2},P_{3},O\right)=\frac{\sum_{i=1}^{n}C_{\mathrm{ABBA}}\left(i\right)-C_{\mathrm{BABA}}(i)}{\sum_{i=1}^{n}C_{\mathrm{ABBA}}\left(i\right)+C_{\mathrm{BABA}}(i)}$$

where *P*_1_, *P*_2_, *P*_3_, and *O* are the four taxa of the comparison. Then, we determined the SE on *D*-statistic across all the conserved clusters by performing a leave-one-out jackknife approach using an R package bootstrap v201204 (Tibshirani and Leisch 2012). For each *D* value, we performed a two-tailed *z*-test to determine if the SE was significantly different from 0. For the SNP data yielded from genome resequencing, we calculated the frequency of the derived allele instead of direct counts for ABBA or BABA loci using a modified equation (Durand et al. 2011):

(2) $$D\left(P_{1},P_{2},P_{3},\;O\right)=\frac{\sum_{i=1}^{n}[\left(1-\hat{P_{i1}}\right)\hat{P_{i2}}\hat{P_{i3}}\left(1-\hat{P_{i4}}\right)-\hat{P_{i1}}(1-\hat{P_{i2}})\hat{P_{i3}}(1-\hat{P_{i4}})]}{\sum_{i=1}^{n}[\left(1-\hat{P_{i1}}\right)\hat{P_{i2}}\hat{P_{i3}}\left(1-\hat{P_{i4}}\right)+\hat{P_{i1}}(1-\hat{P_{i2}})\hat{P_{i3}}(1-\hat{P_{i4}})]}$$

where $\hat{P}$_*ij*_ indicates the allele frequency of SNP *i* in population *j*. For each chromosome, we calculated *D*-statistic for every 50-kb window. We determined SEs and performed *z*-tests for all the chromosomal *D* values with the same method as mentioned earlier.

## Results

### Clustering of Orthologous Genes across *Heliconius* Transcriptomes

We assembled de novo transcriptomes for eight species using RNA extracted from dissected eye tissue of adult females. Our sampling spanned all major *Heliconius* subclades, as well as a closely related outgroup species, *Dryas iulia*. The de novo assembled transcriptome sizes ranged from 62.98 to 111.59 Mb (supplementary table S1, Supplementary Material online). We found evidence of species-specific transcriptome size. *Heliconiuscydno* contained the most reads and yielded the biggest transcriptome, longest isoforms, predicted coding sequences (CDS), and unique genes, whereas *H. sara* contained the second-most reads but yielded the smallest transcriptome. This result suggests a species-specific and tissue-specific transcription pattern rather than a correlation between sequencing depth and size of the assembled transcriptome.

Using the whole data set, we extracted unique genes for each transcriptome and identified 4,042 putative orthologs that were present in all eight species, which we refer in the article as orthologous clusters. We compared the number of 4,042 clusters and the number of annotated genes per chromosome based on a Spearman’s rank correlation test (*P* value = 8.54×10^−7^, supplementary fig. S1, Supplementary Material online), which indicates a strong correlation and suggests the 4,042 clusters a homogeneous representation across the genome. We annotated all the 4,042 clusters and among them there were three opsin clusters, UVRh1 (Cluster 1917), BRh (Cluster 6216), and LWRh (Cluster 683) (supplementary table S4, Supplementary Material online). We did not include UVRh2 in the 4,042 clusters since no UVRh2 was found in the outgroup species *D. iulia*, supporting earlier findings that it evolved via duplication within the *Heliconius* genus (Briscoe et al. 2010). In addition, we identified and annotated 131 unique orthologs only present in the *sara*–*hortense*–*erato* clade and 164 only in the *cydno*–*melpomene*–*hecale* clade (supplementary table S5, Supplementary Material online). The mean lengths of predicted CDS in the orthologous clusters were similar across species; 1,060 bp for *H. hortense*, 1,126 bp for *H. cydno*, 991 bp for *H. sara*, 1,127 bp for *H. doris*, 1,085 bp for *H. hecale*, 1,147 bp for *H. erato*, 1,112 bp for *H. melpomene*, and 1,166 bp for *D. iulia*. There was no obvious correlation between the CDS length and sequencing depth. We further compared them to the mean CDS for all genes in the reference genomes of *H. melpomene* and *Bombyx mori* using the “ortholog hit ratio,” which indicates that the length of most genes has been covered by our orthologous clusters (supplementary fig. S2, Supplementary Material online).

### Topological Incongruence between Transcriptome-Wide, Genome-Wide, and Mitochondrial Phylogenies

**Fig. 1.**
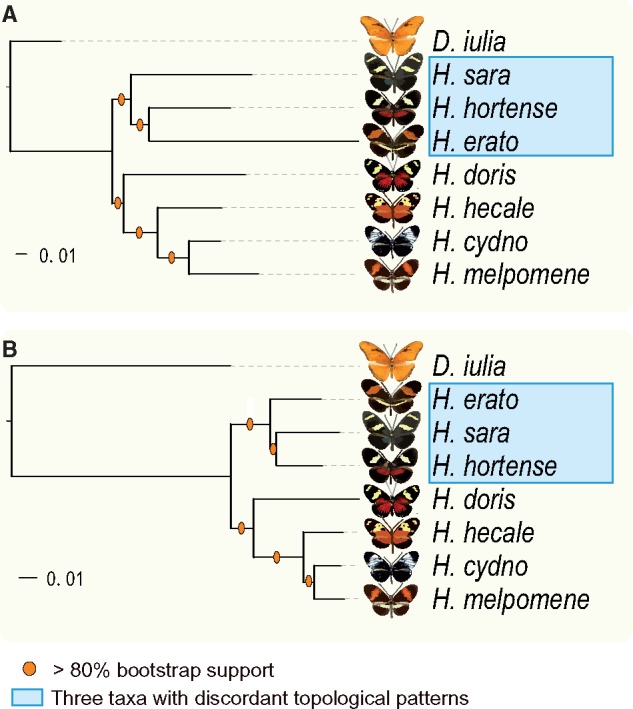
—Transcriptome-wide and mitochondrial phylogenies of *Heliconius* butterflies. The maximum likelihood phylogenetic trees are constructed based on ten mitochondrial genes (*A*) and 4,042 conserved CDS (*B*), respectively. The scale bars represent the percentage of substitutions per site.

In order to be able to test genes under positive selection, we first characterized the evolutionary relationship of our sampled *Heliconius* species, by conducting a transcriptome-wide phylogenetic analysis of our 4,042 concatenated orthologous eye CDS. We also isolated and concatenated ten mitochondrial orthologous genes (*ATP6*, *COI*, *COII*, *COIII*, *cytB*, *ND1*, *ND2*, *ND3*, *ND4*, *ND5*, and *ND6*) for each species and constructed a mitochondrial phylogeny. As expected, the mitochondrial genes yielded the same tree topology reported in studies that utilized multiple loci (Kozak et al. 2015) or multiple chromosomes without divergent genetic regions (Van Belleghem et al. 2017). In all these published topologies *H. hortense* clustered with *H. erato* (fig. 1*A*). Interestingly, our eye transcriptome phylogeny yielded a different relationship with *H. hortense* clustering with *H. sara* rather than with *H. erato* (fig. 1*B*). Both mitochondrial and whole-transcriptome tree topologies were well supported by bootstrap replicates and Shimodaira and Hasegawa test (*P *>* *0.95). We also constructed a transcriptome phylogeny by excluding all the positively selected orthologs and recovered the same topology as shown in figure 1*B* (supplementary fig. S3, Supplementary Material online). Our results reflected an actual incongruence of the relationship among *H. sara*, *H. hortense*, and *H. erato*.

To further characterize this topological incongruence, we extracted 72.9 million genome-wide single nucleotide polymorphisms (SNPs) from 17 whole-genome sequencing data sets and constructed a well-supported phylogeny (supplementary fig. S4, Supplementary Material online). This tree resulted in relationships similar to our mitochondrial phylogeny as opposed to the phylogeny constructed using 4,042 orthologous genes. In general, either incomplete lineage sorting or interspecific introgression could generate a discordant topological pattern. Thus, the topological incongruence of *H. hortense* indicated either introgression or incomplete lineage sorting.

To test the signature of introgression and resolve the relationships among our species, we constructed a time-calibrated phylogenetic tree (supplementary fig. S5, Supplementary Material online). This analysis suggested that the split between *H. hortense* and *H. clysonymus* occurred 0.991 Ma, which was earlier than the splits between *H. erato demophoon* and *H. erato hydara*, which occurred ∼0.822 Ma, and between *H. sara*, *Heliconiuscongener*, and *Heliconiussapho*, dated to 0.907 Ma. Therefore, if introgression occurred and caused phylogenetic incongruence, it probably occurred between ancestral lineages rather than extant species, for example, between an ancestor of *sara–sapho* clade and an ancestor of *erato* subspecies.

### Detecting Introgression and Determining Potential Hybrid Speciation

**Fig. 2.**
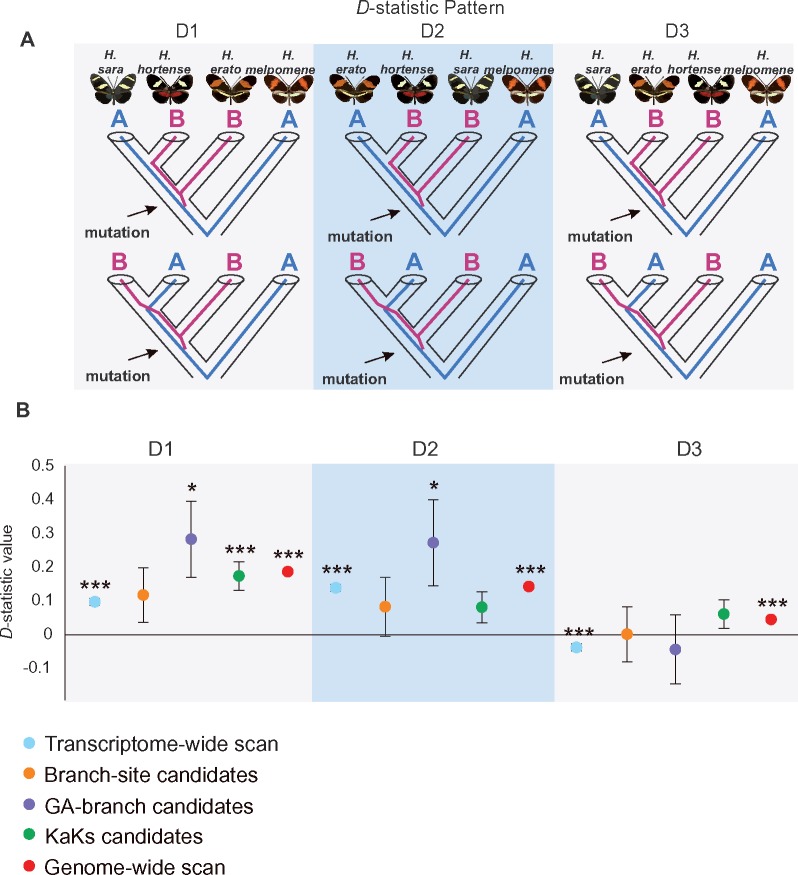
—Patterson’s *D*-statistic patterns among three *Heliconius* species. We calculated *D*-statistic values for each of the three topologies shown above (*A*). The elevated values yielded from *D*_1_ and *D*_2_ of the transcriptome-wide scan, Branch-site candidates listed in supplementary table S9, Supplementary Material online, GA-branch candidates listed in supplementary table S10, Supplementary Material online, KaKs candidates listed in supplementary table S6, Supplementary Material online, and the genome-wide scan indicate putative introgression patterns between *H. hortense* and *H. sara* and between *H. hortense* and *H. erato* (*B*). *P value <0.05; **P value <0.01; ***P value <0.001.

Given that *H. hortense*, *H. sara*, and *H. erato* were the primary taxa involved in the phylogenetic incongruence, we first used Patterson’s *D*-statistic to compare the signatures of allele sharing between *H. hortense* and *H. sara* and between *H. hortense* and *H. erato* across the 4,042 orthologous clusters. We defined three comparisons as *D*_1_ (*sara*, *hortense*, *erato*, *melpomene*), *D*_2_ (*erato*, *hortense*, *sara*, *melpomene*), and *D*_3_ (*sara*, *erato*, *hortense*, *melpomene*) (fig. 2*A*). The transcriptome-wide scan revealed an excess of allele sharing between *H. hortense* and *H. erato*, relative to *H. erato* and *H. sara* (*D*_1_ = 0.0976 ± 0.0110, *P *<* *0.001) and between *H. hortense* and *H. sara*, relative to *H. erato* and *H. sara* (*D*_2_ = 0.1391 ± 0.0109, *P *<* *0.001) (fig. 2*B*). These results suggest similar amounts of introgression between *H. hortense* and *H. erato*, relative to *H. hortense* and *H. sara*. Furthermore, we performed additional analyses for gene data sets with positive signatures of selection and observed similar patterns (fig. 2*B*). Such a signature of transcriptome mosaicism in *H. hortense* indicates that the ancestral lineages of *H. erato* and *H. sara* contributed as potential parental species. This result is similar to a documented introgression pattern among a putative hybrid species *Papilio appalachiensis* and its parental species, *Papilioglaucus* and *Papiliocanadensis* (Zhang et al. 2013).

The above phylogenetic incongruence and signature of allele sharing suggest a potential hybrid speciation event between distantly related species. However, the exact history of this event was not resolved from these analyses. We therefore investigated detailed patterns of hybridization by introducing more candidate comparisons for estimating *D*-statistic in 16 groups including three subspecies of *H. erato*, five species from the *sara–sapho* clade and two parapatric sister species, *H. clysonymus* and *H. hortense*, as well as a few outgroup taxa for each focal clade (fig. 2*B* and supplementary figs. S6 and S7, Supplementary Material online). We here investigated the excess of allele sharing among species within the pupal mating clade (Kronforst and Papa 2015) to further refine the potential common ancestor of *H. clysonymus* and *H. hortense.* We tested lineages increasingly farther from *H. clysonymus/H. hortense* while excluding parental pairs that did not co-occur historically, that is, a young geographic subspecies of *H. erato* and an ancestor of an older pair of species in *charithonia–sara–sapho* (supplementary fig. S5, Supplementary Material online). Interestingly, we did not find any evidence for *H.telesiphe*, the sister lineage of *clysonymus–hortense*, or either for the closer relatives *H. hermathena* and *H. hecalesia.* In both cases, we did not observed a similar signature of mosaicism displayed by *H. hortense* and *H. clysonymus*, but rather no or little evidence of weak hybridization. In summary, our results indicate that the most probable two parental species that originated *H. clysonymus* and *H. hortense* were the common ancestor of the *sara–sapho* and *erato* clades. We here speculate that a possible hybridization occurred between the ancestors of these two clades (*sara–sapho* and *erato*) before the *H. clysonymus* and *H. hortense* diverged.

### Genes under Positive Selection

Using our eye transcriptome topology, we performed multiple tests to identify genes that had experienced positive selection. We first calculated the *K*_a_/*K*_s_ ratio for each of the 4,042 clusters of orthologous genes, which contained 113,176 pairwise comparisons in total and selected candidates with a value >1 for positive selection. This step yielded 672 candidate comparisons representing 276 clusters (supplementary table S6, Supplementary Material online), although a subset of these clusters (163) contained only one candidate branch (supplementary table S7, Supplementary Material online). Comparison of GO terms for this 276 clusters to the total 4,042 clusters (supplementary figs. S8 and S9, Supplementary Material online) suggests some functional enrichment related to development and morphogenesis of eyes (supplementary table S8, Supplementary Material online). We compared these 276 candidates against previously identified gene data sets including opsin-like genes, vision-related genes (92 presenting in 4,042 clusters), sex-biased and positively selected genes, phototransduction genes (23 presenting in 4,042 clusters), and head-upregulated genes (83 presenting in 4,042 clusters) (Macias-Munoz et al. 2016, 2019; Catalan, Macias-Munoz, et al. 2018), which yielded a few number of overlapped clusters (supplementary tables S4 and S6, Supplementary Material online). There were two vision-related genes, *AP-1gamma* and *dan* (clusters 4116 and 7807), and three head-upregulated genes, *DIP-alpha*, *stops*, and *AstA* (clusters 5518, 5770, and 6610), showing signature of positive selection. Generally, these results highlighted very few genes under positive selection, thus suggesting an important and conserved role of genes involved in visual properties. Similarly, except for the 276 clusters under positive selection, most other clusters had a *K*_a_/*K*_s_ ratio <0.5 (supplementary fig. S10, Supplementary Material online), indicative of widespread purifying selection.

**Fig. 3.**
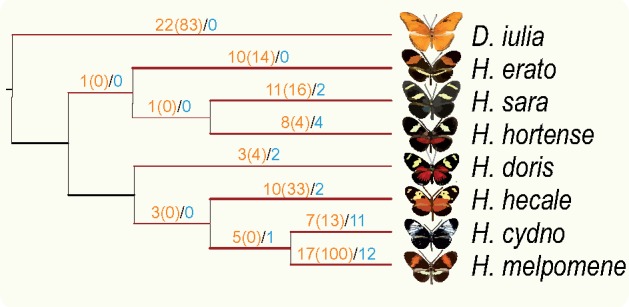
—Positive selection detected in different lineages and internal nodes. We have detected the putative orthologous clusters under positive selection for eight species lineages and four internal nodes. The topology is built according to the tree of concatenated 4,042 clusters and the branches that have been tested are labeled in red. The numbers in orange indicate significant clusters identified using CodeML branch-site model with significant sites enclosed in brackets, whereas the numbers in blue are summarized using GA-branch method.

We then performed further tests for the 276 clusters using the branch-site model and GA-branch method, and manually checked the results in order to remove alignments with a fragment size smaller than 100 bp. For the branch-site model, we chose eight lineages and four internal nodes for each candidate cluster as branches of interest (see Materials and Methods for more detail) and tested one at a time. This analysis included 3,312 tests and yielded a total of 99 significant (*P *<* *0.05) branches across 75 clusters (supplementary table S9, Supplementary Material online). To provide further evidence for positive selection, we performed two random-site tests on these 75 clusters. About 62 of the 75 clusters were supported by comparing M7 and M8 models whereas 57 of them were supported by comparing M8a and M8 models, indicating strong evidence for positive selection on these branch-site candidates (supplementary table S9, Supplementary Material online). For the GA-branch method, we determined the signature of selection across the entire phylogeny, and generated a posterior probability for every branch. We selected candidate branches with a probability >80%. This test yielded 34 significant branches in 29 clusters (supplementary table S10, Supplementary Material online). A total of 13 clusters were supported by both branch-site and GA-branch methods. No GO term emerged as being significantly overrepresented from the functional enrichment of the two candidate data sets supported by either branch-site or GA-branch (*P *<* *0.05). However, results obtained with the branch-site and GA-branch methods displayed a similar pattern of distribution with a greater number of positively selected genes in the younger clade (*cydno–melpomene–hecale*) as compared with the older one (*sara–hortense–erato*) according to a dated phylogeny presented in Kozak et al. (2018) (fig. 3). When we focused on the total significant sites under positive selection for the candidates identified with the branch-site model, we observed again more positively selected sites accumulating in the *cydno–melpomene–hecale* clade (fig. 3 and supplementary table S11, Supplementary Material online). These patterns may suggest that younger lineages show more pronounced adaptive molecular evolution because the transient signature of positive selection gets erased over time.

### Expression Patterns of Positively Selected Genes

**Fig. 4.**
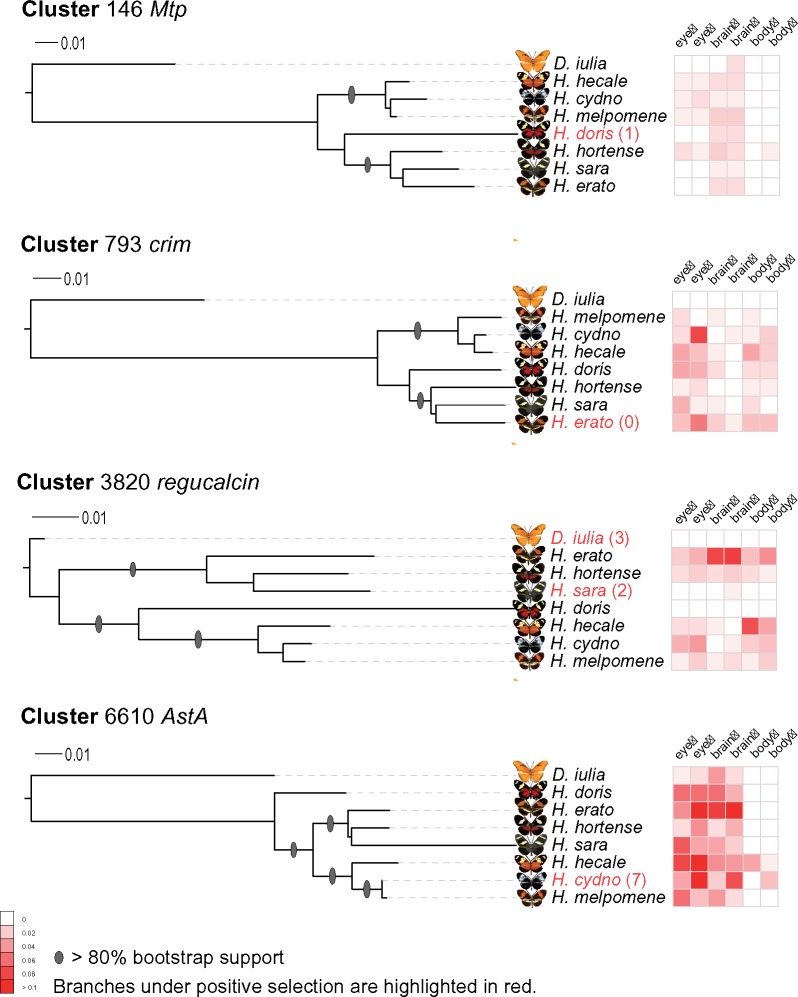
—Tissue- and sex-specific expression of four genes in eight butterfly species. For each gene, we construct a maximum likelihood phylogeny based on its conserved cluster, and the scale bar represents the percentage of substitutions per site. The branches highlighted in red, if any, indicate a significant pattern of positive selection, and the numbers in brackets are significant sites identified using CodeML branch-site model. Each heat map grid stands for relative expression base on a normalized gender- and tissue-specific qRT-PCR result with *n* = 3.

In order to test if our candidates for positive selection display distinct patterns of expression in the eye/brain across species and sex, we searched for orthologs in *Drosophila melanogaster* in Flybase (FB2014_07) (Gramates et al. 2017) that were expressed in head tissue. We selected four representative genes (*Mtp*, *crim*, *regucalcin*, *AstA*) to examine further in *Heliconius* (supplementary tables S12 and S13, Supplementary Material online). *Mtp* is required for lumen expansion in the *Drosophila* tracheal system (Baer et al. 2012); *crim* is essential for septate junction formation as a *Drosophila* Ly6-like protein (Nilton et al. 2010); *regucalcin* is a gene sensitive to day length and associated with photoperiodic regulation and cold tolerance in *Drosophila montana* (Vesala et al. 2012); *AstA is* involved in neuropeptide signaling pathway regulating feeding behavior and metabolism in *Drosophilamelanogaster* (Hentze et al. 2015). We performed quantitative reverse transcription PCR (qRT-PCR) to test the expression profile of these four genes. We used ef-1*a* as a housekeeping gene to normalize mRNA levels in the eye, brain and body tissues of newly emerged male and female butterflies sampled across the *Heliconius* genus with one outgroup species (supplementary table S3, Supplementary Material online). All four target genes were detectable in the eye and brain and body tissues, although in some cases with moderate or relatively weak expression (fig. 4). We performed ANOVA and Tukey’s HSD tests for species-specific, sex-specific, and tissue-specific comparisons for the seven *Heliconius* ingroup taxa. For all the four tested genes, sex-specific and tissue-specific expression patterns differed significantly among the seven *Heliconius* species (ANOVA *P *<* *0.001). However, most of these significant patterns were due to global variation, whereas a smaller number of these patterns were due to a few significant pairwise comparisons, for example, the expression of *Mtp* gene in female brain, *crim* in male brain and female body, *regucalcin* in female brain, and *Asta* in female/male brain and male body (supplementary fig. S11, Supplementary Material online). Zooming in on each species, we observed plenty of differences between female and male expression in different tissues and between sister species. Taking the *crim* gene for example, its sex-specific expression significantly differs in the eyes of each *Heliconius* species and moreover, two sister species show significant but opposite expression patterns with *crim* elevation observed in *H. cydno* male eyes and in *H. melpomene* female eyes. We also observed that the patterns of expression remarkably differed among eye, brain, and body tissues within species, which covers all the gender-specific groups of the seven species when testing *Mtp*, *crim*, and *AstA*. To sum up the above results, the expression of the four targets tended to be highly variable among tissues, species, and even sexes regardless of the focal branch under positive selection. Such variable levels of expression suggest the evolution of complex gene regulation. In other words, these genes might be favored by selection not only on the coding DNA sequences but also in the regulation of their expression.

## Discussion

Here, we characterized the eye transcriptomes of various *Heliconius* species and then used these data to infer patterns of reticulate and adaptive evolution across the genus. First, we demonstrated the utility of RNA-seq to redefine the history of phylogenetic relationship for some members of the genus. Furthermore, we characterized these relationship by performing additional *D*-statistic analyses using genome resequencing data. Our results revealed evidence for an ancient hybrid origin of *H. hortense* and *H. clysonymus* due to hybridization between the *erato* and *sara–sapho* clades. We clustered 4,042 conserved orthologs among eight species with a good coverage of ∼200 vision-related or head-upregulated genes according to previous studies (Macias-Munoz et al. 2016, 2019; Catalan, Macias-Munoz, et al. 2018). The new eye transcriptome phylogeny was important to correctly further characterize candidate eye expressed genes under positive selection. Overall, we identified a group of 276 genes under positive selection and found evidence for more pervasive adaptive protein evolution in younger lineages. Moreover targeted RT-PCR on a subset of these genes suggested that selection could occur simultaneously in the coding sequence and regulatory elements of these genes. Overall our work, together with previous studies (Catalan, Höhna, et al. 2019; Catalan, Macias-Munoz, et al. 2018) provides a list of strong candidate eye expressed genes underlying ecologically evolutionary adaptation.

### Topological Incongruence and the Promise of Whole-Transcriptome Phylogeny

A mitochondrial phylogeny is a conventional standard approach, especially for resolving the relationship between species that have long time diverged. Thus, it does not perform well for recent radiation, since these species tend to have a porous boundary with frequent hybridization (Edelman et al. 2018). In such scenarios, mitochondrial genes only provide a partial view of maternal inheritance, and not the evolutionary history of the entire genome. Even combining standard mitochondrial genes with a few genomic loci is not enough for resolving more complicated lineage-specific relationships when intensive introgression has recently occurred. For example, phylogenetic incongruence has been documented among a couple of *Heliconius* species, including closely related and distantly related species (Baxter 2008; Martin et al. 2013; Zhang et al. 2016; Edelman et al. 2018; Kozak et al. 2018). Therefore, a genome/transcriptome-wide phylogeny is required to reconstruct the complex history. For some extremely entangled *Heliconius* clades such as *besckei–pardalinus–numata* and *telesiphe–sara–hecalesia*, even genome-wide data was not able to provide consistent support for contrasting topologies (Edelman et al. 2018). Nevertheless, those discordant patterns yielded from the partial genome or the mitochondrial genome, if any, cannot be neglected and might be a determining factor to trace other evolutionary events accompanying speciation.

### Detecting Ancient Hybrid Speciation

It is hard to disentangle hybrid speciation, especially among ancient species, which have already become extinct or replaced. The adaptive feature for originating a hybrid species might have been masked by what happened afterward, for example, following hybridization and speciation events and fluctuating selective pressures. The continuous improvement of massive parallel whole-genome sequencing enables scientists to trace possible historical hybridization events with increasing power and precision. However, detecting the signature of hybrid speciation is still challenging. Nevertheless, frequently occurred hybridization and speciation represent a feature of species under rapid radiation. Here, we tested the power of whole-transcriptome sequencing in the *Heliconius* butterfly adaptive radiation and detected a discordant phylogenetic pattern among *H. hortense*, *H. erato*, and *H. sara*. Given that CDS regions were likely to be functional, the transcriptome could be a repository for adaptive genetic material introduced via hybridization and could thereby amplify the discordance. However, a hybrid-species like mosaic transcriptome of *H. hortense* does not permit pinpointing of the exact hybrid speciation event since *H. sara*, one of the candidate parental species, originated later than *H. hortense*. Our additive tests gradually revealed signatures of hybridization among species in *sara–sapho* and *erato* clades, which all pointed to an ancient event occurred among ancestral species. Likewise, similar genome mosaicism has been reported in *H. telesiphe*, which was resolved close to either the *H. hecalesia* or *sara–demeter* subclade (Edelman et al. 2018). With additional admixture tests, the complex topology also indicated a hybrid origin of *H. hecalesia* (Kozak et al. 2018). Interestingly, we noticed that the mosaic pattern yielded from the transcriptome data was less clear relative to the genome data in the form of smaller *D*-statistic values close to 0. This observation indicates a more conservative feature of CDS regions, perhaps leading to neutral or deleterious introgressed material purged more rapidly than in noncoding DNA. The results from putative gene data sets under positive selection also supported the transcriptome mosaicism but with lower statistical power, apparently due to smaller sample sizes.

### More Genes with Signatures of Positive Selection along Young Lineages

Our transcriptome-wide survey for positively selected genes in *Heliconius* eyes highlights more significant genes along younger lineages regardless of the detection methods (fig. 3). Likewise, the substantial branch-specific selection pressure was reported by analyzing orthologous genes among six mammalian species (Toll-Riera et al. 2011). However, the mammalian species were highly diverged to yield a general lineage-specific pattern. In view of recent radiation and similar niche space of *Heliconius* butterflies, our study might serve as a good example for elucidating general features of selection. Positive selection often plays a role as a transient driving force in evolutionary change, after which purifying selection might be responsible for shaping patterns of genetic variation (Murrell et al. 2012, 2015; Lu and Guindon 2014). *Heliconius* encompasses the most species in *Heliconiini* and serves as one of the best examples of adaptive radiation. Recently, Kozak et al. (2015) performed a comprehensive phylogenetic study using 20 nuclear and 2 mitochondrial loci as markers and found an increasing rate of diversification on the branch leading to the *Heliconius* genus. This finding overlaps with our result, which clearly demonstrates that young lineages tend to be the target of stronger positive selection.

By surveying orthologs that were unique to the two clades, *cydno*–*melpomene*–*hecale* and *sara*–*hortense–erato*, we observed a similar pattern: the younger clade *cydno*–*melpomene*–*hecale* contained more unique orthologous clusters. This result also suggests that increased novel gene functions might have formed in the younger clade. However, we could not rule out the possibility that those genes could also exist in other species but are just not detected in the eye tissue. Furthermore, those genes could evolve even faster in other species by generating additional paralogs or becoming too divergent. Then a bad alignment might result in being excluded during the reciprocal best blasting selection step. De novo assembled and annotated genomes of these species will be of great help for better characterizing the clade-specific and even species-specific genes.

### The Variable Expression Patterns among Positively Selected Genes

Another interesting finding that emerged from our study is the variable expression pattern of orthologs that show adaptive signatures of positive selection in at least one lineage. Similarly, a recent study reported that the sex-biased genes in *Heliconius* eye and brain display an interspecific variable expression pattern, as well as significantly higher evolution rates (Catalan, Macias-Munoz, et al. 2018). Note that among the 4,042 clusters, most of the previously identified vision-related or head-upregulated genes are under purifying selection. Five such genes displayed an above-one *K*_a_/*K*_s_ ratio and only one gene, *AstA*, was detected by both *K*_a_/*K*_s_ and branch-site tests and displayed a variable expression pattern. Given so few vision-related genes subject to positive selection in our data set, we present two possible interpretations. First, many vision-related genes subject to positive selection are not present in all eight species and had therefore been excluded from the 4,042 conserved clusters, such as the *Heliconius*-specific opsin gene UVRh2. Second, relative to vision-specific genes, universal or pleiotropic genes might be more likely to be favored or shaped by purifying selection. Similarly, the 4,042 clusters yielded no overlap with the sex-biased genes identified by Catalan, Macias-Munoz, et al. (2018), supporting a species-specific pattern of expression for sex-biased genes proposed by Catalan, Macias-Munoz, et al. (2018).

Despite the vision-related and sex-biased genes, the finding from Catalan, Macias-Munoz, et al. (2018) and our result seems to suggest a possible confounding effect between a variable expression pattern and higher evolutionary rates. This might reveal a predominant feature of these evolutionarily relevant genes, such that both the coding and noncoding regions might be favored by selection in different lineages. Notably, all the candidate genes we tested were not constitutively expressed, thus suggesting that genes with a low or moderate expression level are prone to evolve faster than highly expressed genes. This observation has been already supported by several studies (Krylov et al. 2003; Jordan et al. 2004). Furthermore, given that even new genes can be gradually integrated into an ancestral network and acquire an increasing number of gene partners (Zhang et al. 2015), we have reason to speculate that these fast-evolving genes might also have a chance to trigger the evolution of entire gene networks by reshaping original gene–gene interaction.

## Conclusion

Vision plays an important role in butterfly speciation and adaptation. By surveying eye transcriptomes from representative *Heliconius* clades, we identified a subset of over 200 genes that showed a signature of positive selection. These positively selected genes tended to be in the younger lineages and a subset of fast-evolving loci showed species-specific, tissue-specific, and even gender-specific patterns of expression, suggesting that they might have also been favored as regulatory hotspots. We also described phylogenetic incongruence between mitochondrial and transcriptome-wide phylogenies and further characterized genome mosaicism in *H. hortense* and *H. clysonymus*, indicating an ancient hybridization leading to speciation. Our results depict a transcriptome-wide pattern of evolution in butterfly eyes and shed light on speciation, adaptation, and organismal diversification in the *Heliconius* pupal mating clade. 

### Data Availability

We downloaded Illumina paired-end raw reads from NCBI Sequence Archive (SRA); the accession numbers are PRJNA308754 and PRJNA324415. We also deposited Illumina paired-end raw reads to NCBI Sequence Archive (SRA); the accession number is. Transcriptome assemblis are deposited in the Dryad repository under the accession number doi:10.5061/dryad.0gs7410.

## Supplementary Material

Supplementary data are available at *Genome Biology and Evolution* online.

## Supplementary Material

evz202_Supplementary_Data

## Notes

**Data deposition:** This project has been deposited at NCBI SRA under the accession number PRJNA565507 and Dryad under the accession number doi:10.5061/dryad.0gs7410.
