# Supplementary material for: Comparative Transcriptomics Provides Insights into Reticulate and Adaptive Evolution of a Butterfly Radiation
Source: Genome Biol Evol. 2019 Sep 13;11(10):2963–75. doi: 10.1093/gbe/evz202 (PMC6821300; doi:10.1093/gbe/evz202)
Supplement: evz202_Supplementary_Data [file evz202_supplementary_data.zip › Additional_file_1.pdf]

Table S1. Sample information and transcriptome statistics

ID	Sample	Reads (Gb)	Transcriptome (Mb)	Total Transcripts	Longest Isoform	Predicted CDS	Unique Genes
RP0	*H. hortense*	4.12	93.18	96,789	65,063	35,693	13,483
RP23	*H. cydno*	4.99	111.59	116,342	66,942	40,447	12,822
RP65	*H. sara*	4.45	62.98	84,228	57,348	23,928	12,789
RP83	*H. doris*	2.48	96.06	86,833	51,005	37,187	11,780
RP87	*H. hecale*	2.06	86.62	93,495	56,234	33,167	12,748
RP95	*H. erato*	2.46	78.83	62,962	38,364	26,269	12,987
RP99	*H. melpomene*	2.23	92.53	81,893	49,188	34,677	12,497
RP70	*D. iulia*	3.7	78.27	66,784	40,712	27,680	13,140

Table S2. Sample information and sequencing statistics

Sample	Taxon	Location	Data Source	Reads (Gb)	Genotype Calls (Qual>30)	Mean Depth (Qual>30)
eleu08001	*H. eleuchia*	Ecuador	PRJNA308754	11.74	33847618	15.70
con9125	*H. congener*	Ecuador	PRJNA308754	11.40	33585591	14.75
hew02130	*H. hewitsoni*	Costa Rica	PRJNA308754	11.68	34008714	17.41
saph08100	*H. sapho candidus*	Ecuador	PRJNA308754	11.50	34158665	16.75
sar08025	*H. sara veraepacis*	Ecuador	PRJNA308754	13.32	35440453	40.10
PetED3	*H. erato demophoon*	Panama	PRJNA324415	16.13	37493461	32.57
PetED4	*H. erato demophoon*	Panama	PRJNA324415	15.76	37235859	31.65
PetED5	*H. erato demophoon*	Panama	PRJNA324415	12.31	36390514	26.29
PetED6	*H. erato demophoon*	Panama	PRJNA324415	14.55	37102274	30.00
NCS1179	*H. erato hydara*	French Guiana	PRJNA324415	9.44	36741404	17.50
NCS1979	*H. erato hydara*	French Guiana	PRJNA324415	10.77	37180307	19.45
NCS2080	*H. erato hydara*	French Guiana	PRJNA324415	10.54	37055280	19.22
NCS2211	*H. erato hydara*	French Guiana	PRJNA324415	12.39	37792732	21.30
lativitta01	*H. erato lativitta*	Ecuador	PRJNA324415	11.00	36786026	19.57
lativitta02	*H. erato lativitta*	Ecuador	PRJNA324415	14.03	37747981	24.15
lativitta03	*H. erato lativitta*	Ecuador	PRJNA324415	16.01	37986065	27.64
lativitta04	*H. erato lativitta*	Ecuador	PRJNA324415	16.80	38340105	27.00
hermathena13	*H. hermathena*	Brazil	PRJNA324415	14.30	36945793	20.64
hermathena14	*H. hermathena*	Brazil	PRJNA324415	17.12	37378238	23.52
hermathena15	*H. hermathena*	Brazil	PRJNA324415	20.71	37664137	28.23
STRIWOM2312	*H. hecalesia*	Panama	PRJNA324415	14.90	37394004	23.90
NCS2541	*H. telesiphe*	Peru	PRJNA324415	6.00	34965814	10.83
MCS2550	*H. telesiphe*	Peru	PRJNA324415	11.94	37424329	20.22
MRKhort2	*H. hortense*	El Salvador	PRJNA324415	13.83	36611641	17.71
NCS2511	*H. clysonymus*	Peru	PRJNA324415	9.06	36615512	15.97
NCS2512	*H. clysonymus*	Peru	PRJNA324415	10.97	36874338	19.59
char001	*H. charithonia*	Puerto Rico	PRJNA324415	13.82	33781362	18.68
char002	*H. charithonia*	Puerto Rico	PRJNA324415	7.84	30978129	12.49
BC2004	*H. sara*	Suriname	PRJNA324415	13.10	34756833	17.63
sar003	*H. sara*	Panama	PRJNA324415	6.95	31765389	11.17

Table S3. qRT-PCR primers

Cluster ID	Species	Primers
146	*H. hortense*	146_hort_F, TCAGGCTGTTTTCTTGGACCAAGAGG; 146_hort_R, CTGTTTCATTTGCCTCTCCATCCACA
	H. cydno	146_cydn_F, TCAGGCTGTTTTCTTGAACCAAGAGG; 146_cydn_R, CTGTTTCATTTGCCTCTCCATCCACA
	H. sara	146_sara_F, TCAGGCTGTTTTCTTGGACCAAGAGG; 146_sara_R, CTGTTTCGTTTGCCTCTCCATCCACA
	H. doris	146_dori_F, TCAAGCTATTTTCTTGGACCAAGAGG; 146_dori_R, CTGTTTCATTTGCCTCTCCATCCACA
	H. hecale	146_heca_F, TCAGGCTGTTTTCTTGAACCAAGAGG; 146_heca_R, CTGTTTCATTTGCCTCTCCATCCACA
	H. erato	146_erat_F, TCAGGCTGTTTTCTTGGACCAAGAGG; 146_erat_R, CTGTTTCATTTGCCTCCCCATCCACA
	H. melpomene	146_melp_F, TCAGGCTGTTTTCTTGAACCAAGAGG; 146_melp_R, CTGTTTCATTTGCCTCTCCATCCACA
	D. iulia	146_iuli_F, TCAGTCTGTGTACTTAGACCCTGACG; 146_iuli_R, CTGTTTCATTCCCCTCGCCGTCTACA
793	*H. hortense*	793_hort_F, TGTCCAGATAATGAAGATGTGTGTGT; 793_hort_R, AGCAGCATGGCGACATCCTT
	H. cydno	793_cydn_F, TGCCCAGATAATGAAGATGTGTGTGT; 793_cydn_R, AGCGGCATGGCGACATCCTT
	H. sara	793_sara_F, TGTCCAGATAATGAAGATGTGTGTGT; 793_sara_R, AGCAGCATGGCGACATCCTT
	H. doris	793_dori_F, TGTCCAGATAATGAAGATGTGTGTGT; 793_dori_R, AGCAGCATGGCGACATCCCT
	H. hecale	793_heca_F, TGCCCAGATAATGAAGATGTGTGTGT; 793_heca_R, AGCGGCATGGCGACATCCTT
	H. erato	793_erat_F, TGTCCAGATAATGAAGATGTGTGTGT; 793_erat_R, AGCAGCATGGCGACATCCTT
	H. melpomene	793_melp_F, TGCCCAGACAATGAAGATGTGTGTGT; 793_melp_R, AGCGGCATGGCGACATCCTT
	D. iulia	793_iuli_F, TGTCCAGATAAAGAAGATGTGTGTGT; 793_iuli_R, AGCAGCGTGACGACATCCTT
3820	*H. hortense*	3820_hort_F, AGGGACGCCGGCGCCTATAG; 3820_hort_R, TGGCCTGAAAGGTCCTCAAATCCCA
	H. cydno	3820_cydn_F, AGGGACCCCAGCGCCTGTAG; 3820_cydn_R, TGGCCTGAAAGGTCCTCAAATCCCA
	H. sara	3820_sara_F, AGGGACGCCGGCGCCTATAG; 3820_sara_R, TGGCCCGAAAGGTCCTCAAATCCCA

	H. doris	3820_dori_F, GGGGACTCCGGCGCCCATAG;<br>3820_dori_R, TGTCCTGAAAGGTCCTCAAATCCCA
	H. hecale	3820_heca_F, AGGGACCCCAGCGCCTATAG;<br>3820_heca_R, TGGCCTGAAAGGTCCTCAAATCCCA
	H. erato	3820_erat_F, AGGGACGCCGGCGCTTATAG;<br>3820_erat_R, TGGCCTGGAGTGTCCTCAAATCCCA
	H. melpomene	3820_melp_F, AGGGACCCCAGCGCCTGTAG;<br>3820_melp_R, TGGCCTGAAAGGTCCTCAAATCCCA
	D. iulia	3820_iuli_F, AGGGACGCCGGCGCTTATAG;<br>3820_iuli_R, TGGCCTGAAAGGTCCTCAAATCCCA
6610	*H. hortense*	6610_hort_F, CGGTCTTGGCAAACGCTCAGT;<br>6610_hort_R, CCGAAGCTGTAGGGTCGAGCG
	H. cydno	6610_cydn_F, CGGCCTTGGCAAACGCTCAGT;<br>6610_cydn_R, CCGAAGCTGTAGGGTCGAGCG
	H. sara	6610_sara_F, CGGTCTTGGCAAACGCTCGGT;<br>6610_cydn_R, CCGAAGCTGTAGGGTCGAGCG
	H. doris	6610_dori_F, CGGTCTTGGCAAACGCTCAGT;<br>6610_dori_R, CCGAAGCTGTAGGGTCGAGCG
	H. hecale	6610_heca_F, CGGCCTTGGCAAACGCTCAGT;<br>6610_heca_R, CCGAAGCTGTAGGGTCGAGCG
	H. erato	6610_erat_F, CGGTCTTGGCAAACGCTCAGT;<br>6610_erat_R, CCGAAGCTGTAGGGTCGAGCG
	H. melpomene	6610_melp_F, CGGCCTTGGCAAACGCTCAGT;<br>6610_melp_R, CCGAAGCTGTAGGGTCGAGCG
	D. iulia	6610_iuli_F, CGGTTTGGGCAAACGGTCAGT;<br>6610_iuli_R, CCGAAGCTATATGGTCGAGCT
ef-1a	Universal primers	ef-1a_F, CCAGCTGCCGTCGCTTTCGT;<br>ef-1a_R, CTGGGGGCAAGATGGCGTCG

Table S4. Annotation of 4042 clusters

Cluster ID	Description
1	cell division cycle protein 27 homolog
100	cytoplasmic dynein 1 light intermediate chain 2
1000	antennal esterase cxe18
1003	moesin ezrin radixin homolog 1-like isoform x2
1004	hmct_bommo ame: full=hemocytin ame: full=humoral lectin flags: precursor
1007	ribosomal protein l21
101	exosome complex exonuclease rrp44-like
1010	ca2+ calmodulin-dependent protein kinase ii
1011	protein phosphatase ptc7 homolog
1012	gh regulated tbc protein-1
1013	26s proteasome non-atpase regulatory subunit 2-like
1019	cyclic amp-dependent transcription factor atf-6 alpha
1020	ttc27 protein
1021	hypothetical protein KGM_05402
1023	nuclear receptor
1024	spastin-like
103	dopamine receptor 2-like
1030	dorsal interacting protein 3
1033	wd repeat-containing protein mio-b-like
1035	map microtubule affinity-regulating kinase 3-like
1038	facilitated trehalose transporter tret1-like
104	cyclin-dependent kinase 7-like
1042	pax-interacting protein 1

105	glutamate mitochondrial-like
1050	anoctamin-8-like isoform x5
1052	organic cation transporter
1053	f-box lrr-repeat protein 20-like
1057	nck-associated protein 5
1058 [b]	kinesin-like protein cg14535-like
1059	elongation factor g mitochondrial
106	structural maintenance of chromosomes protein 1a
1061	nucleoside diphosphate kinase 7-like
1062	type i inositol -trisphosphate 5-phosphatase isoform x1
1065	hypothetical protein KGM_08018
1068	u4 u6 small nuclear ribonucleoprotein prp4-like
1069	acyl- -binding domain-containing protein 6-like
107	cyclin g-associated kinase
1070	cactin cg1676-pa
1074	cral trio domain-containing protein
1075	phrf1 protein
108	cda2 isoform a
1080	nucleolar protein 11-like
1083	putative ribophorin ii
1085	mitochondrial glutamate
1086	putative paraplegin
1087	cdgsh iron-sulfur domain-containing protein 2 homolog
1088	protein kinase shaggy-like
1090	transformation transcription domain-associated protein
1092	rho guanine nucleotide exchange factor 10-like

1093	btb poz domain-containing protein 2-like
1094	probable protein phosphatase 2c -like isoform x2
1096	low quality protein: papilin-like
11	antitrypsin isoform 1
1100	multiple epidermal growth factor-like domains protein 10-like
1104	phagocyte signaling impaired
1105	hook protein
1108	tom1-like protein 2-like
1109	vacuolar protein sorting-associated protein 45-like
111	arp2 3 complex 16 kd
1110	protein maelstrom homolog
1111	translational activator gcn1
1115	mediator of rna polymerase ii transcription subunit 27-like
1121	transmembrane protein 68-like
1128	glycosylphosphatidylinositol-specific phospholipase c
1130	mediator of rna polymerase ii transcription subunit 15-like
1131	cold shock domain-containing protein e1
1132	neuropeptide receptor a20
1133	testis-expressed sequence 2
1135	queuine trna-ribosyltransferase
1137	cg14085 cg14085-pa
1138	serine protease inhibitor
114	dnaj homolog subfamily b member 12-like
1141	iroquois-like protein
1142 [b]	forkhead box protein d3-like
115	protein shq1 homolog

1150	homeobox protein prospero prox-1
1151 [a]	beta3 protein
1154	cuticle protein
1158	hypothetical protein KGM_17169
1160	integrator complex subunit 8-like
1161	alpha- sarcomeric-like isoform x2
1162	cytochrome p450
1163	nikkomycin biosynthesis protein p6
1164	guanine nucleotide binding protein
1168	cadherin-87A-like, partial
1171	cytosolic carboxypeptidase-like protein 5-like
1176	hypothetical protein KGM_07245
1178	tonsoku-like isoform x2
1179	e3 ubiquitin-protein ligase trim9-like
118	solute carrier organic anion transporter family member 5a1
1180	polypeptide n-acetylgalactosaminyltransferase 2-like
1185	cell division cycle 7-related protein kinase-like
1186	hypothetical protein KGM_01234
1187	cullin-4b-like
1188	very low-density lipoprotein receptor-like
119	protein wings apart-like
1192	nucleobindin-2-like isoform x1
1193	myotubularin-related protein 3-like
1198	acetyl-coenzyme a synthetase-like
1199	e3 ubiquitin-protein ligase trip12-like
1201	low quality protein: tuberin-like

1205	acid phosphatase-1
1207	brain-specific homeobox protein like protein
1209	lambda-crystallin homolog
121	sister chromatid cohesion protein pds5 homolog b-a-like isoform 2
1211	neuropeptide receptor a24
1212	brain chitinase and chia
1213	glucose transporter
1216	hypothetical protein KGM_15439
1217	protein kinase c alpha binding protein
1218	ell-associated factor 2-like
1219	hypothetical protein KGM_00708
1220	dcn1-like protein 4-like isoform x1
1221	phosphatidylinositol glycan anchor biosynthesis class u
1223	hypothetical protein KGM_15607
1224	ribosome biogenesis protein bop1 homolog
1225	papilin-like isoform x1
1229	gtp-binding protein 1-like
1231	hypothetical protein KGM_04479
1234	tyrosine-protein kinase fps85d-like isoform 2
1235	probable atp-dependent rna helicase ddx56-like
1237	similar to CG15023
1238	hypothetical protein KGM_16023
1239	achi protein
124	peroxisome assembly factor 2-like
1241 [a]	scabrous protein
1244	ecdysone-induced protein 75b

1245	fragile site-associated protein
125	sv2-like protein 1
1251	eukaryotic translation initiation factor 4b-like
1252	leucine-rich repeat serine threonine-protein kinase 1-like
1253	class d atypical g-protein coupled receptor
1257	swi snf-related matrix-associated actin-dependent regulator of chromatin subfamily e member 1-like
1259	elongator complex protein 5-like
1262	golgi-associated pdz and coiled-coil motif-containing
1268	exosome complex exonuclease rrp44-like
1270	high-affinity choline transporter 1-like
1273	nadh dehydrogenase
1274	nadh-ubiquinone oxidoreductase 39 kda subunit
1275	golgi-specific brefeldin a-resistance guanine nucleotide exchange factor 1-like
1276	rna-binding protein musashi homolog rbp6-like
1277	transmembrane protein 50a
1279	male-specific lethal 3
1282	lish domain and heat repeat-containing protein kiaa1468 homolog
1285	splicing factor 3a subunit 3
1287	gram domain-containing protein 1b
1289	nuclear pore complex protein nup133-like
129	patj homolog
1292	wd repeat-containing protein 43-like
1293	protein smg8-like
1294	cuticular protein isoform a
1296	hypothetical protein KGM_06524

1297	proteasome subunit alpha type-3
1298	starry isoform b
1299	zinc finger protein 16-like
13 [a]	neurogenic locus protein delta
130	candidate tumor suppressor protein
1301	hypothetical protein KGM_17479
1302	lysine-specific demethylase 5a
1304	pdgf- and vegf-related factor isoform e
1305	chorion b-zip transcription factor
1308	heat shock 70 kda protein cognate 5-like
1309	hypothetical protein KGM_15578
1311	coenzyme q-binding protein coq10 homolog mitochondrial-like
1312	cg14521 cg14521-pa
1313	set and mynd domain-containing protein 4-like
1315	alpha amylase
1317	cytochrome p450
1318	hypothetical protein KGM_13168
1319	glutathione s-transferase
132	hypothetical protein KGM_07675
1320	phosphatidylinositol transfer protein alpha isoform-like
1326	juvenile hormone epoxide hydrolase
1327	three prime repair exonuclease 1
1330	phosphate transport protein
1332	small heat shock protein
1336	signal recognition particle 72 kda protein
1339	anaphase-promoting complex subunit 5-like

1342	hypothetical protein KGM_08504
1343	synaptobrevin-like isoform x2
1344	takeout jhbp like protein
1345	zinc finger SWIM domain-containing protein 8-like
1347 [a]	serine threonine-protein kinase nlk-like
1349	big zinc finger
1350	protein smaug homolog 2-like
1351	hypothetical protein KGM_18647
1352	hypothetical protein KGM_03031
1355	transmembrane protein 110-like
1356	probable atp-dependent rna helicase spindle-e-like
1359	translation initiation factor if- mitochondrial-like
1360	multiple epidermal growth factor-like domains protein 8-like
1362	nuclease harbi1
1363	centaurin-gamma 1a
1364	docking protein 2
1366	protein cnppd1-like
1368	cuticle protein cpr149
1369 [b]	gtpase-activating rap ran-gap domain-like protein 3-like
137	ferrochelatase, mitochondrial-like
1378	ubiquitin-specific protease
1380	cysteine synthase
1381	adenylate cyclase
1382	50 kda midgut protein
1384	aquaporin
1385	hypothetical protein KGM_05524

1386	probable small nuclear ribonucleoprotein sm d2-like
1389	hypothetical protein KGM_15549
1392	mitochondrial carrier protein
1396	upf0585 protein c16orf13-like protein a
1399	beat- isoform a
14	homeotic protein female sterile-like isoform x2
1401	histone h2b
1402	low quality protein: papilin-like
1403	histone-arginine methyltransferase carmer-like
1411 [a]	fizzy-related protein homolog
1412	skeletal receptor tyrosine protein kinase-like
1414	protein dpy-19 homolog 1
1415	60s ribosomal protein l17
1417	probable e3 ubiquitin-protein ligase mycbp2-like
1418	shc-transforming protein 1-like
142	iron-responsive element-binding protein 1
1421	hypothetical protein KGM_00177
1422	domain-containing histone demethylation protein 2b
1423	integrator complex subunit 5
1425	trehalose transporter 1
1427	n-alpha-acetyltransferase 30-like
1431	pou class transcription factor 2
1432	mitochondrial intermediate peptidase
1433	selenoprotein m-like
1434 [a]	protein giant-lens-like
1435	wing disc-specific protein

1436	werner helicase interacting protein
1437	cg17841 cg17841-pa
1438	bromodomain and phd finger-containing partial
1439	serine threonine-protein kinase sik3-like
1440	annexin ix-c
1443	general vesicular transport factor p115
1446	rna-binding protein 25
1447	protocadherin-like wing polarity protein stan-like
1448	abc transporter
145	suppression of tumorigenicity 5
1451	l-threonine dehydrogenase
146	microsomal triglyceride transfer protein large subunit-like (*Mtp*)
1460	hemolymph proteinase 19
1461	pantothenate kinase
1467	zinc finger protein 271- partial
1472	homeotic protein female sterile-like isoform x2
1474	deoxyhypusine synthase
1475	importin subunit alpha-7-like
1480	pi-plc x domain-containing protein 1-like precursor
1481	septin isoform a
1486	tyrosine-protein phosphatase 3-like
1487	cyclin a
1488	sorting nexin-25-like
149	n -(beta-n-acetylglucosaminyl)-l-asparaginase
1492	probable xaa-pro aminopeptidase 3-like
1493	ferric-chelate reductase 1 homolog

1494	cell growth regulator with ring finger domain protein 1-like
1495	rad50-interacting protein 1-like
1496	lipoma preferred partner lpp
15	pre-mrna-processing factor 40 homolog a-like
1502	mitochondrial atp synthase f chain
1505	mrna-decapping enzyme 1b
1506	protoporphyrinogen oxidase
1507	pancreatic triacylglycerol lipase
1508	neuroblastoma-amplified sequence
1509	cathepsin d
1512	hypothetical protein KGM_12011
1513	protein stoned-b-like
1517	cub and sushi domain-containing protein 3
152	ras-related protein 2
1522	probable chitinase 3-like
1523	nudt1 protein
1526	phagocyte signaling-impaired protein
1528	wd repeat-containing protein 19-like
1529	choline-binding surface protein a
153	seryl-trna mitochondrial
1530	antennal esterase cxe17
1533	uncharacterized protein C3orf33-like isoform X1
1534 [a]	kayak isoform a
1535	hypothetical protein KGM_18677
1536	hypothetical protein KGM_04341
1537 [b]	hexosaminidase d-like

1539 [a]	camp-dependent protein kinase catalytic subunit
1540	tbc1 domain family member 9
1541	ribonucleoprotein
1542	dna damage-binding protein 1-like
1543	26s proteasome non-atpase regulatory subunit 7-like
1545	26s proteasome non-atpase regulatory subunit 13
1546 [a]	Paired box protein Pax-7
1547	tudor domain-containing protein 15-like
1548	serine threonine-protein kinase chk2
1550	protein crumbs-like
1552	enolase partial
1555	serine threonine-protein kinase tao1
156	pleiotrophin-like protein
1561	lethal 35di
1568 [c]	serine threonine protein kinase akt
1569	succinyl- ligase
157	bmp-binding endothelial regulator
1570 [a]	adenylyl cyclase-associated protein
1571	cytosolic malate dehydrogenase
1574	hypothetical protein KGM_19203
1576	dna helicase mcm8
1580	histidine triad nucleotide-binding protein 1
1581	beat- isoform a
1583	a disintegrin and metalloproteinase with thrombospondin motifs 3-like
1585	hypothetical protein KGM_11495

1588	beige beach domain containing protein
1589	ubiquitin-like protein 7-like
1590	ring finger protein
1592	protein lethal denticleless-like
1593	fatty-acyl reductase 4
1594 [b]	glutamate receptor kainate 2-like
1597	hemolymph proteinase 17
1599	short-chain dehydrogenease reductase
1601	myb-like protein x-like
1602	amino acid transporter
1604 [a]	rna-binding protein 15b-like
1607	eukaryotic peptide chain release factor gtp-binding subunit erf3a
1610	tetratricopeptide repeat protein 28-like
1611	golgin subfamily a member 7-like
1612	adamts-like protein 4
1615	metallophosphoesterase 1-like
1619	mitogen-activated protein kinase kinase
1620	neuronal acetylcholine receptor subunit alpha-7-like
1626	gata-binding factor c-like
1628	tbc1 domain family member 22b-like
1629	vacuolar h
163	beat protein
1630	cuticular protein analogous to peritrophins 3-a2 precursor
1632 [a]	phenylalanine hydroxylase
1634	dipeptidase 1-like
1637	leucine-rich repeat-containing protein 4b

1638	probable splicing arginine serine-rich 7-like
1639	hypothetical protein KGM_14984
1642 [a]	paired box protein pax-
1644	deoxyribodipyrimidine photo-lyase-like
1645	sideroflexin 1
1648	nad dependent epimerase dehydratase
1649	dna polymerase epsilon
1650	tubulin-specific chaperone e
1651	potassium channel subfamily t member 1-like
1652	pyruvate dehydrogenase
1655	tnf receptor associated factor
1658	smc2 protein
1660	fat-like cadherin-related tumor suppressor-like protein
1661	non-lysosomal glucosylceramidase-like
1662	yorkie homolog
1664	low quality protein: e3 ubiquitin-protein ligase hectd1-like
1666	eukaryotic translation initiation factor 5b-like isoform x1
1667	inner nuclear membrane protein man1
1670	hdd1-like protein
1672	hermansky-pudlak syndrome 1 protein homolog
1675	wd repeat domain phosphoinositide-interacting protein 2 isoform x2
1676	uncharacterized protein LOC101736398
1680	spondin-1-like isoform x1
1682 [a]	protein pygopus
1683	usher syndrome type-1g protein homolog

1685	protein enl-like
1688	n -(beta-n-acetylglucosaminyl)-l-asparaginase
1689	jarid1a protein
169	rhomboid-related protein 3-like
1690	diphthamide biosynthesis protein 2-like
1691	phosphatidylinositol 4-kinase alpha
1693	No hits found
1694	synaptic vesicle protein
1698	hypothetical protein EAI_15424
170	protein transport protein sec31a-like
1700	histone h2a
1702	sarcoglycan isoform a
1705	circadian isoform a
1712	lipopolysaccharide-induced tumor necrosis factor-alpha factor homolog
1715	No hits found
1716	hypothetical protein KGM_07582
1717	cysteine-rich protein 2-binding
1719	nadh-ubiquinone reductase
172	zinc binding dehydrogenase
1720	mitochondrial thioredoxin 2
1721	nadh-ubiquinone oxidoreductase 75 kda subunit
1722	low quality protein: krab-a domain-containing protein 2
1723	e3 ubiquitin-protein ligase hectd1
1726	profilin
1727	atp-binding cassette sub-family g member 1-like

173	neutral ceramidase-like isoform x1
1733	cg11318 cg11318-pa
1737	protein mo25
174	guanine nucleotide-releasing factor 2-like
1740	lamin dm0-like
1742	apoptotic protease-activating factor 1-like
1744	nfx1-type zinc finger-containing protein 1-like
1745	hypothetical protein KGM_15110
1746	nitrogen permease regulator 3-like
1748 [a]	histidine decarboxylase-like
1749	enoyl- hydratase
175	map kinase phosphatase
1750	zinc finger protein 14
1751 [a]	ap-1 complex subunit beta-1
1752 [c]	pyruvate dehydrogenase
1753	dual 3 -cyclic-amp and -gmp phosphodiesterase 11-like
1754	general transcription factor ii-i repeat domain-containing protein 2-like protein
1755	succinate dehydrogenase
1759 [b]	protein-s-isoprenylcysteine o-methyltransferase-like
1763	hypothetical protein KGM_08238
1764 [c]	innexin inx3
1768	pupal cuticle protein precursor
177	guanine nucleotide-binding 3 homolog
1770	tyrosine-protein phosphatase non-receptor type 13
1771	serine threonine-protein kinase mig-15

1772	wd repeat-containing protein 92
1773	periodic tryptophan protein 2 homolog
1774	histone deacetylase complex subunit sap18
1776	protein charybde-like
1777	dalr anticodon-binding domain-containing protein 3-like
1779	26s proteasome non-atpase regulatory subunit 6-like
1781	serine arginine repetitive matrix protein 2-like isoform x1
1782	btb poz domain-containing protein 3-like
1784	hypothetical protein KGM_08284
1786	nadh-ubiquinone oxidoreductase 75 kda mitochondrial-like
1788	acid phosphatase
179	dna mismatch repair protein msh2-like
1790	hypothetical protein KGM_12934
1794	ecdysteroid 22-kinase
1799	t-complex protein 1 subunit delta-like
180	spondin-1-like
1800	inorganic phosphate cotransporter-like
1804	serine protease -like
1805	u3 small nucleolar rna-interacting protein 2
1806	26s protease regulatory subunit 6b
1809	cellulosome anchoring protein cohesin region
1812	nose resistant to fluoxetine protein 6-like
1814	forkhead box subgroup partial
1815	lysophospholipase-like protein 1-like
1818	synaptic vesicle protein
1819	wd repeat-containing protein 20 isoform x1

1820	p38 map kinase
1821	btb poz domain-containing protein kctd9-like
1822	swi snf-related matrix-associated actin-dependent regulator of chromatin subfamily a containing dead h box 1 homolog
1825	equilibrative nucleoside transporter
1826	nuclear protein localization protein 4 homolog
1827	coagulation factor xi
1828	nitric oxide synthase
183	low quality protein: dual specificity protein phosphatase mpk3-like
1831	unknown unsecreted protein
1833	importin beta-3
1835	guk- isoform b
1837	zinc finger ccch-type with g patch domain-containing
184	wd repeat-containing protein 26
1841	transformation transcription domain-associated
1842	dnaj homolog subfamily c member 16-like
1845	eukaryotic translation initiation factor 4e nuclear import factor 1
1846	amino acid transporter
1847	synaptic vesicle membrane protein vat-1 homolog-like
1848	splicing factor 3b subunit 1-like
1849	ras-related protein rab-2a
1851	guanine nucleotide-binding protein subunit beta-5-like
1852	signal peptidase complex subunit 2
1854	ras-related protein rab-10-like
1857	histone deacetylase
1859	nuclear receptor nhr-48

186	protein dead ringer-like
1863	liquid isoform i
1865	cell division cycle 5-like
1868	nuclear receptor co-repressor 1
1869	seven in absentia
187	mitochondrial processing peptidase alpha subunit
1870	serine threonine-protein phosphatase 6 regulatory subunit 3-like
1871	pupal cuticle protein
1873 [b]	ethanolaminephosphotransferase 1-like
1874	high mobility group
1876	cuticular protein 49ae
1877	otu protein
188	hypothetical protein KGM_16066
1882	collagen alpha-1 chain-like
1885	(6-4) photolyase
1887	pax-interacting protein 1
1888	utp-glucose-1-phosphate uridylyltransferase 2
1889	protein c12orf4
1890	ubiquitin thioesterase trabid-like
1891	transcription factor castor
1893	hypothetical protein KGM_06762
1894	nadh-ubiquinone oxidoreductase 42 kda subunit
1895	carboxypeptidase q-like
1896	guanylate cyclase
19	signal peptidase 18 kda subunit
190	histone acetyltransferase kat2a-like

1900	ran-binding protein 3-like
1901	denn domain-containing protein 1a-like
1902	crumbs
1903	diacylglycerol kinase
1907	serine threonine protein kinase
1908 [b]	hypothetical protein KGM_06609
1909	elmo domain-containing protein 2-like
191	ubx domain containing 2
1912	longitudinals lacking
1914	zinc finger protein 791-like
1917 [b, c]	ultraviolet-sensitive visual pigment (UVRh1)
192	protein abrupt-like
1920 [a, b, c]	arrestin homolog
1922 [b]	cellular retinoic acid binding protein
1924	abnormal wing disc-like protein
1925	TPPP family protein CG4893-like isoform X1
1926	splicing factor 3b subunit 4-like
1928	leucine-rich repeats and immunoglobulin-like domains protein 3
193	t-complex protein 1 subunit eta-like
1930	nicotinic acetylcholine receptor a6 subunit
1931	programmed cell death
1935	esterase fe4
194	low quality protein: microtubule-associated serine threonine-protein kinase 2-like
1944	serine threonine-protein kinase unc-51-like isoform x2
1945	patatin-like phospholipase domain-containing protein 2-like

1946	hypothetical protein KGM_00635
1948	iron-sulfur cluster assembly 2 mitochondrial-like
1951	maternal protein pumilio
1952	piezo-type mechanosensitive ion channel component 2-like
1953	arsenite-resistance protein
1954	programmed cell death 7
1955	ste20-related kinase adapter protein alpha-like
1961	mitochondrial prohibitin complex protein 2
1966	c2 domain-containing protein 5-like
1969	squamous cell carcinoma antigen recognized by t-cells 3-like
1972	nitrogen permease regulator 3-like
1973	maltose phosphorylase
1974	lethal isoform a
1975	quaking related 54b
1976	proliferation-associated protein 2g4-like
1979	charged multivesicular body protein 5
1980	hypothetical protein KGM_07888
1981	salivary protein mys2 precursor
1984	n-glycosylase dna lyase
1988	e3 ubiquitin-protein ligase listerin-like
1990	adhesion-regulating molecule 1
1991	methionine sulfoxide reductase
1992	hemicentin-1-like
1993	protein fam214a
1994	suppressor of hairless
1998	dnaj homolog subfamily a member 2-like

200	72 kda inositol polyphosphate 5-phosphatase-like
2002 [a]	ras-related protein rab-11a-like
2005	carboxyl-terminal pdz ligand of neuronal nitric oxide synthase
2006	serine threonine protein
2007	dentin sialophospho isoform x1
2008	serine threonine-protein kinase 40
201	alpha-tocopherol transfer
2016	hypothetical protein KGM_11518
2017	arsenite-resistance protein
2018	importin-11 isoform x2
2019	differentially expressed in fdcp 6-like protein
2023	endoplasmic reticulum resident protein 29-like
2024	phosphatidylinositol 4-kinase beta-like
2026	rho guanyl-nucleotide exchange factor
2029	growth arrest-specific protein 1-like
203	dosage compensation regulator
2031	leucine-rich repeat-containing protein 16a
2032	uncharacterized protein LOC101741884, partial
2033	nfx1-type zinc finger-containing protein 1-like
2034	protein tipin homolog
2035	phosphatidylinositol 3-kinase catalytic subunit type 3
2036	ubiquitin-like protein 4a-like
2038	calcium calmodulin-dependent protein kinase type 1
2041	eukaryotic translation initiation factor 2-alpha kinase 4-like
2042	bumetanide-sensitive sodium-(potassium)-chloride cotransporter-like, partial

2043	trithorax group protein osa
2045	pdz domain-containing
2046	nicotinic acetylcholine receptor beta 21c
2051	segmentation protein cap n collar-like
2056	protein unc-80 homolog
2057	serpin 1
2059	peptide-n -(n-acetyl-beta-glucosaminyl)asparagine amidase
206	myosin-i heavy chain
2062	solute carrier family facilitated glucose transporter member 3-like
2063 [a]	abl interactor 2
2065	dna-binding protein ets97d-like
2066	multiple coagulation factor deficiency protein 2 homolog isoform x1
2068	upf0439 protein c9orf30-like protein
2072	hypothetical protein KGM_20757
2073	sarcolemmal membrane-associated
2078	No hits found
2080 [a]	vacuolar protein sorting-associated protein 33a-like
2085	pyrroline-5-carboxylate reductase
2086	coiled-coil domain-containing protein 132-like
2094	spz1B
2095	lim and sh3 domain protein lasp-like
2097	protein efr3 homolog cmp44e-like
210	glutamate receptor 1-like
2100	general transcription factor iie subunit 1-like
2103	inactive dipeptidyl peptidase 10-like

2104	transcription factor hnf-4 homolog
2108	nuclear pore complex protein nup205
2109 [a]	sine oculis
211	39s ribosomal protein mitochondrial-like
2111	neurogenic locus notch homolog protein 3-like
2112	say domain-containing protein 1-like
2113	leucine-rich repeat-containing protein c10orf11-like protein
2114	kxdl motif-containing protein cg10681-like
2116	microtubule-associated protein jupiter-like isoform x2
2117	dihydrolipoamide dehydrogenase
2119	hypothetical protein KGM_15216
2120	actin-related protein 10-like
2121	putative armadillo protein
2122	f-box only protein 42
2123	copper homeostasis protein cutc homolog
2125	eukaryotic translation initiation factor 2a-like
2128	hypothetical protein KGM_15415
2130	kinase suppressor of ras 2-like
2132	2-type zinc finger
2134	programmed cell death protein 5-like protein
2135	No hits found
2136	late secretory pathway protein avl9 homolog
2140	striatin-3 isoform x4
2142	atp-dependent rna and dna helicase
2143	sox domain-containing protein dichaete-like
2145	hypothetical protein KGM_14812

2147	hypothetical protein KGM_08736
2148	ras gtpase-activating protein 1
215	lim domain-binding protein
2150	hypothetical protein KGM_10198
2151	glutamine synthetase 2 cytoplasmic-like isoform x2
2152	uncharacterized protein LOC101744858
2154	50-kda dystrophin-associated glycoprotein
2158	yellow-d
2159	acyl-CoA dehydrogenase family member 9, mitochondrial-like isoform X1
216	hypothetical protein KGM_20540
2161	protein rolling stone-like
2162	tho complex subunit 1-like
2168	transcription initiation factor tfiid subunit 6
2169	sp1070 cg9138-pa
2170	AMP-activated protein kinase
2171	protein bric-a-brac 2
2172	aael004946- partial
2175	insulin receptor
2176	leucine-rich transmembrane protein
2177	integrator complex subunit 7-like
2179	transcription factor btf3 homolog 4-like
2184	ankyrin repeat-containing protein
2186	hypothetical protein KGM_17728
2188	glutamyl-trna amidotransferase subunit b
2189	ribosomal protein l19

2190	homeotic protein female sterile-like isoform x2
2193	dihydrolipoamide succinyltransferase component of 2-oxoglutarate dehydrogenase
2196	c-cbl-associated protein isoform a
2197	proteasome subunit beta type-5-like
2198	cd109 antigen
22	cask isoform a
2200	insulin-like growth factor ii
2201	udp-n-acetylglucosamine pyrophosphorylase
2202	nuclear pore complex protein nup155
2204	mitochondrial glutamate carrier 1-like
2205	lutropin-choriogonadotropic hormone receptor
2207	ran-binding protein 3-like
2210	rho gtpase activating protein 21
2211	ornithine decarboxylase
2213	6-phosphogluconate decarboxylating
2214 [b]	hypothetical protein LOC100119541
2215	mitochondrial ribonuclease p protein 3-like
2216	hypothetical protein KGM_18691
2217 [a]	chimerin
2218	protein saal1-like
2219	eukaryotic translation initiation factor 3 subunit 4
2223	programmed cell death 6-interacting protein
2224	luc7-like protein 3-like
2228	scavenger receptor class b member 3
223	ww domain-binding protein 11-like

2230	transferrin
2231	tau-tubulin kinase 1-like
2232	methenyltetrahydrofolate synthase domain-containing
2233	epidermal growth factor receptor substrate 15-like 1-like
2234	thioredoxin peroxidase
2239	putative alpha-(1,6)-fucosyltransferase
2241	bric a brac isoform c
2242	replication factor c subunit 5
2245	thioester-containing protein 3
2246	nucleosome-remodeling factor subunit nurf301
2247	probable cardiolipin synthase-like
2250	probable e3 ubiquitin-protein ligase mycbp2
2252	stomatin-like protein mitochondrial-like
2257	anoctamin-8-like isoform 1
2259	vacuolar protein sorting-associated protein 13b
226	transmembrane protein 39a-like isoform x2
2264	v-type proton atpase subunit d-like
2267	dolichyl pyrophosphate man9 c2 alpha- -glucosyltransferase
2268	interphase cyctoplasmic foci protein 45
227	heparan sulfate glucosamine 3-o-sulfotransferase 3a1-like
2270	importin subunit alpha-7
2273	organic cation transporter
2274	protein ptcd3 mitochondrial-like
2276	tetraspanin 47f
2279	nephrin-like
2280	peptidyl-alpha-hydroxyglycine alpha-amidating lyase 1-like

2282	hypothetical protein KGM_20905
2283	thymidylate synthase
2288	protein tyrosine phosphatase 1
2291 [b]	mitogen-activated protein kinase kinase kinase kinase 4-like isoform x5
2292	oxidase peroxidase
2293	lactoylglutathione lyase
2294	protein tipe-like
2295	coatomer subunit epsilon
2299 [a]	protein lozenge-like
23	oxysterol-binding protein 1
2300	la-related protein 6-like
2301	low quality protein: microtubule-associated serine threonine-protein kinase 2-like
2302	gtp-binding protein rad
2303	ring finger protein 17-like
2304	dna repair protein xrcc4
2305 [b]	probable g-protein coupled receptor cg31760-like
2306	uncharacterized protein LOC101739289
2308	ecdysteroid 22-kinase
2311	folylpolyglutamate mitochondrial-like
2313	dihydrolipoamide acetyltransferase component of pyruvate dehydrogenase
2314	probable e3 ubiquitin-protein ligase mycbp2-like
2315	low quality protein: midasin-like
2317	aryl hydrocarbon receptor nuclear translocator homolog
2318	probable e3 ubiquitin-protein ligase mycbp2-like

2320	multiple epidermal growth factor-like domains protein 11-like
2321	gtp-binding protein ypt7-like
2322	glutamate carboxypeptidase
2327	probable sulfite mitochondrial-like
2329	CG31140, isoform E
2331	1-beta dynein
2333	insulin-like growth factor-binding protein complex acid labile subunit-like
2335	hypothetical protein KGM_05742
2339	glycerol kinase
2340	hypothetical protein KGM_02037
2342	hemolymph proteinase 5
2346	nf-kappa-b-repressing factor-like
2348	swi snf complex subunit smarcc2
2349	wd repeat-containing protein 35 isoform x2
235	lipoyltransferase mitochondrial-like
2350	synaptotagmin-16-like
2351	ribosomal protein l23a
2352	kynurenine aminotransferase
2353	protein flightless-1-like
2357 [b]	y+l amino acid transporter 2-like
2359	calcium channel flower-like isoform 2
236	ras guanine nucleotide exchange factor p-like isoform x3
2360	hemocyte protein-glutamine gamma-glutamyltransferase-like
2361	synaptotagmin-like protein 4-like
2363	serine protease 7

2365	upf0369 protein c6orf57 homolog
2370	interferon-related developmental regulator 1-like
2371	uncharacterized protein CG7816-like
2372	heterogeneous nuclear ribonucleoprotein
2373	ribosomal protein l7a
2375	serotonin receptor
2379	ribosomal protein s20
238	probable domain-containing histone demethylation protein 2c-like
2381	speckle-type poz isoform x2
2385	kinesin-like protein k39-like isoform x1
2386	transcription factor hes-4-like
2387	wd repeat and fyve domain-containing protein 2-like
2389	protein rrp5 homolog
239	ran gtpase-activating protein 1-like
2393	chitooligosaccharidolytic beta-n-acetylglucosaminidase
2394	serotonin receptor
2395	xpo6 protein
2396	hypothetical protein KGM_07546
2397	nuclear fragile x mental retardation-interacting protein 1
24	wd repeat-containing protein 91-like
240	tRNA methyltransferase
2400	exocyst complex component 2
2406	dna polymerase subunit gamma mitochondrial
2407	adenylosuccinate synthetase-like
2409	fibrillin 2
241	glutathione s transferase s1

2410	hypothetical protein KGM_03414
2412	ring finger protein 126-like
2415	metaxin-2
2417	protein phosphatase 1 regulatory subunit 37-like
2418	mob kinase activator-like 4-like
242	nucleoporin gle1-like isoform x1
2420	eukaryotic initiation factor 4e-2
2421	patj homolog
2425	organic cation transporter
243	hypothetical protein KGM_11178
2430	calcium/calmodulin-dependent 3',5'-cyclic nucleotide phosphodiesterase 1C-like
2431	rb1-inducible coiled-coil protein 1-like
2433	inhibitor of bruton tyrosine kinase isoform x2
2437	eater
2438	elongation factor tu
2439	myrosinase 1-like
2440	protein yipf1-like
2441	ubiquitin-protein ligase e3c-like
2443	tpa: cuticle protein
2444	cleft lip and palate transmembrane protein 1-like
2446	superoxide dismutase
2447	rna-binding protein fusilli-like
2448	neuroligin-4, Y-linked-like
2449	condensin complex subunit 3-like
245	4-aminobutyrate aminotransferase

2450	hypothetical protein KGM_22301
2452	hypothetical protein KGM_01980
2453	will die slowly
2454	ras gtpase activating protein
2455	putative uridine 5'-monophosphate synthase
2456	ubiquinol-cytochrome c reductase
2457	mucin-5ac-like isoform x3
2458	modifier of mdg4-like isoform X6
2460	protein turtle-like
2462	cadherin-related tumor suppressor
2464	Beta-1,3-glucan-binding protein
2469	flocculation protein flo11-like
247	set and mynd domain-containing protein 4-like
2470	protein smg8-like
2472	calcineurin a
2473	voltage-gated calcium channel alpha2-delta subunit 1
2475	putative survivin
2478 [a]	mitogen-activated protein kinase kinase kinase 7
2479	multiple ankyrin repeats single kh domain protein
248	cation chloride cotransporter
2480	Rapgap1, isoform F
2484	ras-related c3 botulinum toxin substrate 1-like
2485 [b]	leucine-rich repeat-containing protein 15-like
2486	hypothetical protein KGM_18639
2488	ubiquitin carboxyl-terminal hydrolase 38
2489	putative beta-1,4-galactosyltransferase

2490	e3 ubiquitin-protein ligase topors-like
2491	igf2 mrna binding protein
2494	rho-related btb domain-containing protein 1-like
2495	centrosomin-like isoform x2
2498	spatacsin isoform x1
250	probable phenylalanine--trna mitochondrial-like
2501	No hits found
2503	beta-glucosidase precursor
2507	monocarboxylate transporter
2508	hypothetical protein KGM_09242
251	ubiquitin carboxyl-terminal hydrolase 22-like
2510	short-chain dehydrogenase
2511	phosphate transporter
2512	eukaryotic translation initiation factor 2d isoform 1
2513	fas apoptotic inhibitory molecule 2
2514	organic cation transporter
2515	guanylate cyclase
2516	protein creg1-like
2517	rho-associated protein kinase 2
2518	pacifastin light chain
2520	probable g-protein coupled receptor 158-like
2522	mitochondrial processing peptidase beta subunit
2523	zinc finger ran-binding domain-containing protein 2-like
2528	charged multivesicular body protein 7-like
2531	large neutral amino acids transporter small subunit 2
2534	importin-11-like isoform x1

2535	olfactory receptor 29
2542	chitin deacetylase 4
2545	uncharacterized protein LOC101741676
2546 [b]	sodium- and chloride-dependent gaba transporter 1-like
2547	p21-activated protein kinase-interacting protein 1-like
2548	serine threonine-protein kinase mtor
2549	zinc finger protein 622-like
2550 [a]	enhancer of split mgamma
2551	tata element modulatory factor
2552	g patch domain-containing protein 1 homolog
2558	tuftelin-interacting protein 11
2559	glycerophosphoryl diester periplasmic
256	hypothetical protein KGM_15509
2562	protein spire-like
2563	general transcription factor 3c polypeptide 2
2564	low quality protein: nuclear factor related to kappa-b-binding
2566	ankyrin unc44
2567	facilitated trehalose transporter tret1-like
2569	cytochrome p450
2570	melanization protease 1
2571	hypothetical protein KGM_18645
2573	gpi mannosyltransferase 3-like isoform x2
2577	three prime repair exonuclease 1
2578	venom acid phosphatase
2580	ribosome biogenesis protein bop1 homolog
2581	zinc finger protein 830-like

2582	bsd domain-containing protein 1
2588	rna-binding protein squid-like isoform x8
259	steroid receptor-interacting snf2 domain protein
2591	cg3560
2595	peroxiredoxin prdx5
2596	yellow-h3
2598	hypothetical protein KGM_11066
26	peroxisomal biogenesis factor 3
260	probable atp-dependent rna helicase ddx46-like
2601	gpi ethanolamine phosphate transferase 2-like
2602	protein lap2 isoform x2
2603	probable u3 small nucleolar rna-associated protein 11-like
2604	e3 ubiquitin-protein ligase nedd-4-like isoform 2
2607	biorientation of chromosomes in cell division protein 1-like 1-like
2609	mitochondrial cardiolipin hydrolase-like
2611	protein asterix-like
2621	histone-lysine n-methyltransferase setmar-like
2624	mediator of rna polymerase ii transcription subunit 20-like
2625	protein kinase
2627	histone h4 transcription factor
2628	lysm and peptidoglycan-binding domain-containing protein 1-like
2630	p160 coactivator fisc
2632	fatty-acid amide hydrolase 2-like
2634	exportin-1
2635	transcription initiation factor tfiid subunit 2-like
2636	hypothetical protein KGM_00630

2639	diuretic hormone class 2-like
2641	angiomotin
2642	alpha-tocopherol transfer
2644	phosphorylated ctd-interacting factor 1-like
2647	putative acetyl-CoA acetyltransferase, mitochondrial
2648	hypothetical protein KGM_01853
265	hypothetical protein KGM_10436
2650	malate dehydrogenase
2651	mothers against decapentaplegic homolog 5 isoform x2
2653	protein dom3z-like
2655	basic helix-loop-helix protein
2657 [c]	protein kinase brain isozyme-like
2659	synapse-associated protein
2660	leucine-rich ppr motif-containing mitochondrial-like
2661	26s protease regulatory subunit s10b
2663	membrane associated progesterone receptor
2664	hypothetical protein KGM_11257
2672	tectonin beta-propeller repeat-containing
2674	dna damage-binding protein 1-like
2677	leucine-rich repeat protein
2679	vacuolar h
268	elongation factor 1 gamma
2681	ankyrin repeat and btb/poz domain-containing protein 2-like
2684	egalitarian
2686	cyclic amp response element-binding protein a-like
2687	e3 ubiquitin-protein ligase amfr-like

269	graves disease carrier protein homolog
2691	prophenoloxidase subunit 1
2692	protein o-mannosyl-transferase 2-like
2693	tumor suppressor p53-binding protein 1
2694	upf0392 protein
2695	zinc finger protein
2698	hypothetical protein KGM_08685
27	serum response factor
270	adiponectin receptor 2
2700	udp-glucosyltransferase precursor
2702	rna-binding protein 40-like
2706	hypothetical protein KGM_05600
2708	protein phosphatase PP2A 55 kDa regulatory subunit-like isoform X2
271	homeotic protein female sterile-like isoform x3
2710	glycylpeptide n-tetradecanoyltransferase 1-like
2711	synaptotagmin-15-like
2712	cytochrome p450
2716	serine protease inhibitor partial
2718	orexin receptor type 2-like
272	host cell factor 1
2720	armadillo repeat-containing protein 8
2721	hypothetical protein KGM_01355
2722	calcium release-activated calcium channel protein 1-like
2723	protein sarah-like isoform x1
2726	domon domain-containing protein cg14681-like

2727	low quality protein: src substrate protein p85-like
2728 [b]	homeobox protein hmx1
2730	membrane-associated guanylate ww and pdz domain-containing protein 2
2731	atp-dependent protease la
2732	tetraspanin 29fb
2735	vacuolar protein sorting-associated protein 13b-like
2736	exosome complex component mtr3-like
2737	coiled-coil-helix-coiled-coil-helix domain-containing protein mitochondrial-like
2739	nuclear factor 1 x-type-like isoform 1
2740	fat-like cadherin-related tumor suppressor homolog
2743	cell division control protein 45 homolog
2744	cell division protein
2745	collagen alpha-2 chain-like
2748	moxd1 homolog 1-like
2749	ell complex eap30 subunit
2750	protein lin-10
2751	zinc finger protein 709
2752	zinc finger protein 26
2754	e3 ubiquitin-protein ligase sina-like 9
2755	amp dependent coa ligase
2756	lysine-specific demethylase 8-like
2757	cytochrome p450
2758	ferm domain-containing protein 5-like
2759	transforming acidic coiled-coil containing protein 1 2
2761	dna polymerase epsilon catalytic subunit a-like

2763	fasciclin-1-like
2764	histone-lysine n-methyltransferase setdb1-like
2765	nedd4 family-interacting protein 1-like
2766	zinc finger protein
2767	hypothetical protein KGM_04304
2768	ap-2 complex subunit alpha-like
2772	hypothetical protein KGM_17725
2777	unconventional myosin-ixa-like
2781	golgin subfamily a member 2-like
2783	dual specificity mitogen-activated protein kinase kinase 6-like
2786	nudt18 protein
2787	cytochrome p450 cyp18a1
2788	hypothetical protein KGM_01960
2789	hypothetical protein KGM_10931
279	uncharacterized protein LOC101735376
2790	atypical protein kinase c
2792	zinc finger protein jing-like protein
2793	proto-oncogene tyrosine-protein kinase ros
2795	sugar transporter
28	brain tumor
2800	hypothetical protein KGM_21406
2806	chemosensory protein
2809	adenosine deaminase related growth factor
281	chromatin-remodeling complex atpase chain iswi-like
2810	myosin vii
2811	aaa atpase

2815	sorting nexin-25-like
2816	signal peptidase complex subunit 3
2817	zinc finger protein on ecdysone puffs
2819	adenylate cyclase
2821	hypothetical protein KGM_05223
2822	eukaryotic translation initiation factor 3 subunit a-like
2826	serine threonine-protein kinase minibrain-like
2828	xylulose kinase
283 [b]	down syndrome cell adhesion molecule-like protein dscam2-like
2830	protein bicaudal d
2831	uncharacterized protein LOC101737779
2835	serine threonine-protein kinase rio3 (rio kinase 3)
2836	39s ribosomal protein mitochondrial-like
2846	glycogen phosphorylase
2848 [a, b, c]	sodium calcium exchanger 3-like
2849	ribosomal protein l5
285	u7 snrna-associated sm-like protein lsm11-like
2850	ras-like gtp-binding
2853	c3 and pzp-like alpha-2-macroglobulin domain-containing protein 8
2854	monocarboxylate transporter
2855	cg1702
2857	beta amyloid protein precursor- isoform f
2859	an1-type zinc finger protein 6-like isoform x1
2860	meiotic recombination 11
2861	wd repeat-containing protein cg11141-like

2863	uncharacterized protein LOC101737983
2864	early growth response
2867	hypothetical protein KGM_00493
2868	protein ergic-53-like
287	glutaminyl-peptide cyclotransferase-like
2870	mrna turnover protein 4 homolog
2872	yellow-f4
2873	lim homeobox protein lhx9-like isoform x1
2874	cytochrome p450
2879	ankyrin unc44
288	aminoacylase-1-like
2880	takeout jhbp like protein
2884	dna replication licensing factor mcm3
2885 [a]	protein phosphatase 1 regulatory subunit 12a-like isoform x5
2886	probable atp-dependent rna helicase spindle-e-like
2889	guanylate cyclase 32e-like
289	eph receptor
2890	peroxisomal acyl-coenzyme a oxidase 3-like
2891	synoviolin-like protein
2894	glucose dehydrogenase
2896	hypothetical protein KGM_21659
2899	protein kinase c and casein kinase substrate in neurons protein 2-like isoform 1
29	zinc finger ccch domain-containing protein 13-like isoform x1
290	ankyrin repeat and fibronectin type-iii domain-containing protein 1
2906	probable chitinase 3-like

2909	shaker cognate isoform b
2911	dna mismatch repair protein pms2
2912	glutamate-rich wd repeat-containing protein 1-like
2914	phosphatidylinositide phosphatase sac1-like
2916	tyrosine-protein kinase drl-like
2919 [b]	neuroligin-1 isoform x1
292	negative elongation factor a-like
2921	oxysterol-binding protein 9
2922	lish domain and heat repeat-containing protein kiaa1468-like
2924	protein smg5
2927	cg33515 cg33515-pa
2928	discs large isoform l
2930	general transcription factor iih subunit 3-like
2936 [a]	kynurenine 3-monooxygenase
2937	cg9135-pa
2938	uncharacterized wd repeat-containing protein alr3466
2942	angiotensin converting enzyme
2948	adenylate cyclase
295	poly(adp-ribose) glycohydrolase
2951	hypothetical protein KGM_05396
2952	fibroblast growth factor receptor substrate 2-like
2955	lim homeobox protein lhx9-like
2956	rna polymerase ii subunit a c-terminal domain phosphatase ssu72-like
296	ras-related protein rab-28
2962 [a, c]	neural stem cell-derived dendrite regulator

2964	hypothetical protein KGM_14542
2965	rab32
2966	hrp65 protein
2967	nuclear pore complex protein nup205
2969	band 7 protein agap004871-like
2970	e3 ubiquitin-protein ligase march2
2971	digestive organ expansion factor homolog
2974	oxysterol-binding protein 9-like
2977	tuberous sclerosis 2 isoform 3
2979	protein turtle homolog a-like
2982	intron-binding protein aquarius-like
2983 [a, b, c]	calmodulin-binding protein trpl
2985	hypothetical protein KGM_17376
2987	unpaired-like partial
299	family protein
2991	heat repeat-containing protein 5b-like
2992	zinc finger protein 268
2993	multiple inositol polyphosphate phosphatase 1-like
2996	arrestin domain-containing protein 3-like
2997	zinc finger protein 37-like
2998	dim gamma-tubulin 3
2999	glucose-1-phosphatase inositol phosphatase
3	fgfr1 oncogene partner
3004	facilitated trehalose transporter tret1-like
3008	transmembrane protein 39a-like isoform x1
3012	membrane-associated guanylate kinase

3013	dna-directed rna polymerase i subunit rpa2-like
3014	serine threonine-protein kinase vrk1-like
3015	transmembrane and tpr repeat-containing protein 1-like
3016	aminopeptidase n-like
3018	transmembrane protein 205-like
3019	alanine aminotransferase
3022	nuclear pore complex protein nup85-like
3023	dnaj homolog subfamily c member 3-like
3024	atp-dependent rna helicase p62-like
3027	alanine--glyoxylate aminotransferase 2-like
3029	bifunctional protein ncoat-like isoform x2
303	probable rna-binding protein orb2-like
3030	zinc finger and scan domain-containing protein 4
3032	hypothetical protein KGM_10434
3036	endonuclease-reverse transcriptase
304	ddb1- and cul4-associated factor-like 1-like
3040	multiple c2 domain and transmembrane region isoform e
3041	cytoplasmic dynein 1 intermediate chain-like isoform x5
3042	seminal fluid protein cssfp034
3043	neuropeptide receptor a23
3046	myocyte-specific enhancer factor 2d
3048	cytochrome c oxidase subunit va
305	protein big brother-like
3050	atp synthase delta mitochondrial
3051	hypothetical protein KGM_03957
3052	mrna cap-binding protein eif4e

3053	heat repeat-containing protein 3-like
3054	voltage-dependent calcium channel subunit alpha-2 delta-3-like
3055	protein turtle-like
3057	myo-inositol-1 phosphate synthase
3062	hypothetical protein KGM_11445
3066	chitin deacetylase-like isoform h
3068	sodium-independent sulfate anion transporter-like
307	hypothetical protein KGM_04929
3077	wd repeat-containing protein 47-like
3081	hypothetical protein KGM_07404
3082	probable g-protein coupled receptor mth-like 5-like
3083	isoleucyl-trna mitochondrial-like
3084	28s ribosomal protein mitochondrial
3086	protein phosphatase 2c
3088	tyrosine-protein phosphatase non-receptor type 23-like
3090 [a]	ets dna-binding protein pokkuri-like
3092	tensin-1 isoform x14
3093	monocarboxylate transporter
3096	arf-gap with ank repeat and ph domain-containing protein 1
310	low quality protein: baculoviral iap repeat-containing protein 6-like
3100	matrix metalloproteinase-25-like
3106	coiled-coil domain-containing protein 102a
3108	tetratricopeptide repeat protein 28
3114	arf6 guanine nucleotide exchange factor
3116	ommochrome binding protein 1

3117	serine protease
3118	insulin-like growth factor-binding protein complex acid labile chain
3119	uncharacterized protein LOC101737485
3120 [b]	dual specificity tyrosine-phosphorylation-regulated kinase
3126	zinc transporter zip9-like
3127	beaten path iia
3132	rna exonuclease 1 homolog
3133	synaptotagmin-like protein 4-like
3135	atpase family aaa domain-containing protein 5-like
3138	kv channel-interacting protein 4-like
314	zinc finger protein 420-like
3141	partitioning defective 3
3142	protein dok-7
3147	39s ribosomal protein mitochondrial
3148	protein toll-like
315	pericentriolar material 1 protein
3150	gdp-fucose protein o-fucosyltransferase 2-like
3156 [b]	c4b-binding protein beta chain
3157	uridine phosphorylase 1-like isoform 1
3159	protein diaphanous
316 [a]	sumo ligase
3163	myotubularin-related protein 13
3164	heparanase-like protein
3165	phosphoglycerate mutase
3167	zinc finger protein

3169	39s ribosomal protein mitochondrial-like
317	tpa: cuticle protein
3170	probable ras gtpase-activating
3172	alcohol dehydrogenase
3178	Incenp
318	zinc finger protein
3185	facilitated trehalose transporter tret1-like
3191	calreticulin
3192 [a]	gp150 protein
3193	protein sidekick-like
3194	ferritin light chain
3198	hypothetical protein KGM_19674
320	sphingosine kinase b
3202	adenylate cyclase
3204	heat shock protein
3205	insulin gene enhancer protein isl-1-like
3206	bone morphogenetic protein receptor type-1b-like
3207	hypothetical protein KGM_20157
321	cathepsin b
3211	e3 ubiquitin-protein ligase mib1
3212	cdc73 domain protein
3214	mitochondrial ribosomal protein s31
3215	speckle targeted pip5k1a-regulated poly polymerase-like
3217	myosin-ib-like isoform x2
3218	metabotropic glutamate receptor 4-like
322	centrin

3223	endoplasmic reticulum aminopeptidase 2-like
3225	solute carrier organic anion transporter family member 4a1-like
3227	rho gtpase-activating protein 21
3230	cdk5 and abl1 enzyme substrate 1-like isoform x3
3232	thyroid adenoma-associated protein homolog
3233	hypothetical protein KGM_08735
3234	cytochrome b5-related
3244	luciferin 4-monooxygenase-like
3246	ribosome-recycling mitochondrial-like
3247	93 kda serpin
325	dna replication licensing factor mcm7-like
3252	acyl-protein thioesterase
3255	bm trachealess
3257	hypothetical protein KGM_06385
326	ubiquitin-conjugating enzyme e2
3260	alpha-2-macroglobulin-like protein 1-like
3263	repressor splicing factor 1
3265	homogentisate -dioxygenase
3266	lix1-like protein
3267	transmembrane emp24 domain-containing protein eca-like
3270	sugar transporter
3276	rad50-interacting protein 1
3279	apyrase
3285	myosin-viia-like isoform 2
3286	palmitoyltransferase zdhhc5-like isoform x2
3288	histone h3 methyltransferase

3289	cg12128 cg12128-pa
329	hypothetical protein KGM_16230
3292	glutathione s-transferase omega 1
3293	nuclear distribution protein
3295	nicotinic acetylcholine receptor subunit alpha7
3296	hippocampus abundant transcript 1 isoform x2
3298	transmembrane protein 115-like
3299	transmembrane 7 superfamily member 3-like
33	ribosomal protein s10
330	wd-repeat protein
3300	multiple ankyrin repeats single kh domain
3301	large subunit gtpase 1-like protein
3303	bullous pemphigoid antigen isoforms 6 9 10
3304	UDP-N-acetylglucosamine:alpha-6-D-mannoside beta-1,2-N-acetylglucosaminyltransferase II
3305	protein archease-like
3308	a disintegrin and metalloproteinase with thrombospondin motifs 2
3309	hairy cell leukemia
3311	atp-binding cassette sub-family f member 2
3312	e3 ubiquitin-protein ligase ubr4
3314	dna-directed rna mitochondrial-like
3315	nedd8-activating enzyme e1 catalytic subunit
3316	serine arginine repetitive matrix protein 1-like
3318	serine threonine-protein kinase d3
332	protein tanc2-like
3321	cell cycle regulator mat89bb homolog

3322	serologically defined colon cancer antigen 3-like protein
3325	viral a-type inclusion protein
3326 [a]	camp-dependent protein kinase catalytic subunit
3329	spermine oxidase-like
3331	j domain-containing protein
3332	chromatin modification-related protein eaf-1-like isoform x2
3336	protein fam136a-like
3337	protein spire-like
3341	speckle-type poz protein
3344	protein fam86a-like
3345	transmembrane protein 62
3346	kyphoscoliosis peptidase
3348	apolipoprotein of lipid transfer particle-i ii
335 [b]	cys-loop ligand-gated ion channel subunit
3351	methionine--trna cytoplasmic-like
3352	protein fam160b1-like
3357	zinc finger protein 91- partial
3361	nuclear pore complex protein nup88-like
3363	hypothetical protein KGM_04798
3364	hypothetical protein KGM_22211
3366 [a]	epidermal growth factor receptor
3367	hypothetical protein KGM_09832
3368	eukaryotic translation initiation factor theta subunit
337	flavone synthase i
3372	lectin c-type domain containing protein
3373	nuclear receptor-binding protein homolog

3374	dna-damage inducible protein
3375	cg6123 cg6123-pa
3376 [b]	43 kda receptor-associated protein of the synapse-like
3378	hypothetical protein KGM_19708
338	exocyst complex component 7-like
3380	transmembrane and tpr repeat-containing protein cg4050-like
3385	dentin sialophospho isoformx1
339	e3 ubiquitin-protein ligase ubr4
3392	coiled-coil domain-containing
3393	zinc finger protein 320
3394	dynamin-like 120 kda mitochondrial-like
3396	ubiquitin carboxyl-terminal hydrolase 14-like isoform 1
3397	s1 rna-binding domain-containing protein 1
3399	an1-type zinc finger protein 1-like
34	protein tamozhennic
3400	phosphoserine phosphatase isoform x2
3404	hypothetical protein KGM_02640
3405	low quality protein: kinesin-like protein kif13a-like
3409	neurobeachin-like
3411	exonuclease gor-like partial
3413	neutral sphingomyelinase-like
3414	short-chain dehydrogenase
3419	leucine-rich repeat-containing protein ddb_g0290503-like
3422	n-alpha-acetyltransferase auxiliary subunit-like
3423	camp-specific 3 -cyclic isoforms n g isoform x4
3427	hypothetical protein KGM_01235

3429	accumulation-associated protein
343	dna topoisomerase iv subunit a
3430	protein dok-7
3431	integrin beta pat-3 precursor
3433	leukotriene a-4 hydrolase
3435	isocitrate dehydrogenase
3437	z band alternatively spliced pdz-motif protein isoform b
3438	wd-repeat protein
3440	cg1676
3442	hypothetical protein KGM_01415
3444	aromatic amino acid decarboxylase
3445	mitotic spindle assembly checkpoint protein mad2a-like
3446	nucleoplasmin-like protein
3447	leucine-rich repeat-containing protein 24-like isoform x1
3449	kinesin-associated protein
3454	rna-binding protein 39-like isoform x2
3455	swi/snf-related matrix-associated actin-dependent regulator of chromatin subfamily a-like protein 1-like
3456	integrin beta-ps-like
3457	methylglutaconyl- mitochondrial-like
3459	nedd8 ultimate buster 1-like
3460	metastasis-associated protein 3
3465	lachesin-like isoform x4
3467	aquaporin transporter
347	homeobox protein pknox2
3470	ring finger protein 157-like

3471	glucose-6-phosphate 1-epimerase-like
3472	uncharacterized protein LOC101742051
3477	carbohydrate sulfotransferase 4-like
3479 [a]	rab5 gdp gtp exchange factor
348	phosphatidate phosphatase
3481	nck-interacting protein with sh3 domain-like
3482	odorant-binding protein
3483	pleckstrin-like proteiny domain-containing family a member 5
3484	runt-like protein
3485	cuticular protein glycine-rich 10 precursor
3486	ethanolamine-phosphate cytidylyltransferase
3487	transmembrane and tpr repeat-containing protein cg4050-like
3488	mothers against decapentaplegic homolog 6-like
3489	activating signal cointegrator 1
3490	wd repeat-containing and planar cell polarity effector protein fritz-like
3493	39s ribosomal protein mitochondrial-like
3494	fetal alzheimer falz
3497	putative survivin
3498	double-stranded rna-binding protein zn72d
3499	hypothetical protein KGM_08436
350	low quality protein: transcription factor hamlet-like
3505	probable atp-dependent rna helicase dhx35-like
3506	cytochrome b5
3508	uncharacterized protein LOC101737035
3510	trafficking kinesin-binding protein milt-like

352	phosphoglucomutase-like isoform x2
3520	dedicator of cytokinesis protein 9
3521	macroglobulin complement-related
3522	uncharacterized family 31 glucosidase kiaa1161-like
3523	exocyst complex component 5
3524	n-acetylgalactosamine kinase
3526	putative Myeloid differentiation primary response protein MyD88
3527	hypothetical protein KGM_14419
3529	anaphase-promoting complex subunit 15 isoform b
3532	ras-gef domain-containing family member 1b-like
3534	neurogenic locus notch homolog protein 3-like
3536	leucine zipper protein
3537	protein takeout-like
3538	probable 28s ribosomal protein mitochondrial-like
3546	hypothetical protein KGM_07418
3547	cg17950
3549	inositol monophosphatase 3-like
3550	josephin-like
3551	synaptic vesicular amine transporter-like
3552	u3 small nucleolar rna-associated protein 18 homolog
3553	diphthamide biosynthesis protein 1-like
3556	chromosome transmission fidelity protein 18 homolog
3558	putative goliath E3 ubiquitin ligase
3559	peroxisome proliferator-activated receptor binding protein
356	hypothetical protein KGM_15027
3560	cullin-associated nedd8-dissociated protein 1

3562 virilizer-like protein

3564 integrator complex subunit 6-a

3565 lissencephaly-1 homolog

3566 nipped-b-like protein

3567 vesicle transport through interaction with t-snares homolog 1a-like

3569 atp-dependent helicase brm-like

3570 pftaire-interacting factor isoform h

3571 5-formyltetrahydrofolate cyclo-ligase

3572 probable phosphorylase b kinase regulatory subunit beta-like

3573 t-complex protein 1 subunit beta-like

3574 caseinolytic peptidase b protein homolog

3575 cyclin dependent kinase 4

3576 tpa: cuticle protein

3577 peroxisomal targeting signal 1 receptor-like

3578 rab proteins geranylgeranyltransferase component a 1-like

3581 ras-related protein rab-30-like

3582 pre-mrna-splicing factor slu7-like

3583 glucosylceramidase-like

3586 e3 ubiquitin-protein ligase march6-like

3587 low quality protein: liprin-alpha-1-like

359 ras-related protein rab-27a

3591 prenylated rab acceptor protein 1

3592 phosphatidylinositol 4-kinase beta-like

3593 proteasome inhibitor

3596 histone-lysine n-methyltransferase mll5

3597 gtp-binding protein rhes

36	protein kinase c-binding protein nell1
360	rho guanine nucleotide exchange factor 11-like
3601	map kinase-activated protein kinase 2-like
3603	ubx domain-containing protein 8
3604	bendless
3607	nuclear y ccaat-box binding factor c subunit nf yc
3608	eukaryotic translation initiation factor 3 subunit l-like
361	retinal homeobox protein rx-like
3611	putative survivin
3614	protein sde2 homolog
3616	lysosomal trafficking regulator
3618	transmembrane 9 superfamily member 2-like isoform x2
3619	sina homologue
362	ribonuclease p protein subunit p20-like
3620	cullin-associated nedd8-dissociated protein 1-like
3621	histone deacetylase complex subunit sap130-like isoform x1
3622	partitioning defective 6 homolog gamma-like
3623	rho gtpase-activating protein 190-like
3624	coiled-coil domain-containing protein 85c-like
3626	hypothetical protein KGM_21255
3629	luciferin 4-monooxygenase
3631	clathrin coat assembly protein
3632	brain chitinase and chia
3636	nodal modulator 2
3637	trna 2-thiocytidine biosynthesis protein ttca
3638	juvenile hormone binding protein

3639	mixed-lineage leukemia protein
364	metallophosphoesterase 1 homolog
3640	sip1-tfip11 interacting protein
3641	chemosensory protein
3642	conserved oligomeric golgi complex subunit 4-like
3643	integrin-linked protein kinase-like
3644	colorectal mutant cancer isoform x1
3645	anopheles gambiae pest agap012803-pa
3646	ubiquitin-protein upl3
3648	osiris 24
3649	protein prrc2a-like
3651	atm interactor
3653 [b]	myosin iii
3654	verprolin-like isoform x1
3657	extramacrochaetae protein
3658	nadh dehydrogenase 1 alpha subcomplex subunit 5
366	zinc finger protein 347
3661	ribosomal protein l35a
3663 [a]	ap-1 complex subunit mu-1-like
3664	cg3563 cg3563-pa
3667	hypothetical protein KGM_00234
3668	similar to CG2519
367	coiled-coil domain-containing protein 47-like
3670	ceramide glucosyltransferase-like
3671	hypothetical protein KGM_15943
3674	troponin c

3677	tyrosine-protein kinase dnt-like
3680	dna-binding protein satb1
3681	sorting nexin-16-like
3685	nadh:ubiquinone reductase 23kd subunit precursor
3688	von hippel-lindau disease tumor suppressor-like
3690	protein atonal-like protein 8
3693	hypothetical protein KGM_04135
3695	uncharacterized protein LOC101744379 isoform X1
3697	gelsolin-like
3700	brahma associated protein 170kd
3701	charged multivesicular body protein 4b-like
3706	angiotensin-converting enzyme
3707	rho gtpase activating protein
3710	melanotransferrin-like
3711	low quality protein: water dikinase-like
3713	adp-ribosylation factor-like protein 4c-like
3714	nuclear excision repair protein rad23
3717	malate dehydrogenase
3718	esf1-like protein
3720	guanine nucleotide exchange factor
3721	camp-dependent protein kinase catalytic
3724	No hits found
3725	d-2-hydroxyglutarate mitochondrial-like
3727	eh domain-containing protein 1-like
3728	protein ect2-like
373	visceral mesodermal armadillo- isoform b

3731	maternal effect protein staufen-like
3734	transmembrane protein 147-like
3735	lachesin-like isoform 2
3736	cuticular protein 100a
3738	fatty-acid amide hydrolase 2-like
3739	malate mitochondrial-like
374	histone deacetylase complex subunit sap30 homolog
3741	glucose-induced degradation protein 4 homolog
3742	protein turtle-like protein a
3743	uncharacterized protein LOC101736142
3744	hypothetical protein KGM_14539
3745	6-phosphofructo-2-kinase fructose- -biphosphatase-like
3746	cyclic amp response element-binding protein a-like
3747	2-oxoglutarate dehydrogenase
3749	nucleosomal histone kinase 1-like
375	eukaryotic translation initiation factor 3 subunit e
3751	inositol triphosphate 3-kinase c
3754	hypothetical protein KGM_12759
3755	cral trio domain-containing protein
3756	dna replication atp-dependent helicase nuclease dna2-like
3757	uncharacterized protein LOC101735960
376	chitobiosyldiphosphodolichol beta-mannosyltransferase-like
3760	uncharacterized protein LOC101743259
3761	cd109 antigen-like
3764 [b]	down syndrome cell adhesion molecule-like protein dscam2-like
3765	mitochondrial inner membrane

3768	ubiquitin-conjugating enzyme e2 c
3769	transmembrane protein 181-like
377	phosphatase and actin regulator
3771	uncharacterized protein LOC101736695, partial
3772	ommochrome binding protein 2
3773	proteasome subunit beta type-4-like
3774	microsomal glutathione s-transferase-like
3778	26s proteasome non-atpase regulatory subunit 11-like
378	probable 28s ribosomal protein mitochondrial-like
3784	aliphatic nitrilase
3786	nsfl1 cofactor p47
3789	tho complex subunit 2
379	protein brunelleschi-like
3792	uncharacterized protein LOC101741223 isoform X1
3801	cirhin isoform x1
3803	protein cepu-1-like
3804	polypeptide n-acetylgalactosaminyltransferase 3-like
3805	uncharacterized protein LOC101738864
3806	probable maltase d-like
3807	low quality protein: myosin heavy non-muscle-like
3808	growth differentiation factor 11
3809	prohibitin protein wph
381	e3 ubiquitin-protein ligase hectd1-like
3811	actin-related protein 2 3 complex subunit 4
3812	wd-repeat protein
3813	protein phosphatase 2c

3816	insulin receptor
3818 [a]	slingshot dual specificity phosphatase
3819	cytochrome p450
3820	luciferin-regenerating enzyme, *regucalcin*
3821	transcriptional adapter 2b-like
3823	eukaryotic translation initiation factor 3 subunit b
3826	probable g-protein coupled receptor 158-like
3827	serine threonine-protein kinase haspin homolog
383	high mobility group protein a
3830	actin-related protein 5-like
3832	wd repeat-containing protein 46-like
3833	facilitated trehalose transporter tret1-like
3834	cullin-associated nedd8-dissociated protein 1
384	formin cappuccino
3843 [b]	down syndrome cell adhesion molecule-like protein dscam2-like
385	rho guanine nucleotide exchange factor 10-like
3850	metabotropic glutamate receptor 4-like
3851	zinc finger fyve domain-containing protein 26-like
3852	outer dense fiber protein 3
3856	origin recognition complex subunit 5
3857	nuclear pore complex protein nup85-like
3859	proteasome subunit beta type-6-like
3861	uncharacterized protein LOC101735576
3863	cell division cycle protein 23 homolog
3866	potassium channel subfamily k member 3-like
3867	nucleolar complex protein 4-like protein b

3873	aspartyl-trna synthetase
3874	cg7453 cg7453-pa
3875	palmitoyltransferase zdhhc17-like
3876	ring finger protein 17-like
3879	hypothetical protein KGM_21514
3885	general transcription factor iif subunit 1-like
3886	tar dna-binding protein 43-like
3887	cg6459
3888	zinc finger protein 845-like
389	kinase d-interacting substrate of 220 kda-like
3890	nucleoporin nup53
3891	sl allatostatin a receptor
3892	guanine nucleotide-binding protein subunit beta-like
3893	ubiquitin carboxyl-terminal hydrolase 46-like
3894	protein suppressor of hairy wing-like
3895	cg5466 cg5466-pa
3896	f-box only protein 28-like
3897	tetraspanin-5-like isoform x2
39	atp-dependent rna helicase bel
3903	18 wheeler
3905	sulfate transporter
3906	protein rtf2 homolog
391	histone-lysine n-methyltransferase setd1b
3912	proteasome subunit beta type-2-like
3913	LOW QUALITY PROTEIN: neurobeachin-like
3919	peptidylglycine alpha-hydroxylating monooxygenase-like

3920 [a]	transforming protein p54 c-ets-1
3921	bud13 homolog
3922	rho-associated protein kinase 2
3923 [a]	mediator of rna polymerase ii transcription subunit 12
3928	plexin domain-containing protein 2-like
3931	serine threonine-protein kinase 3-like
3934	protein sda1 homolog
3935	translation initiation factor if- mitochondrial-like
3937	regulator of microtubule dynamics protein 1-like
3938	pre-mRNA-processing factor 39-like
3939	lipase 3-like
3940	hypothetical protein KGM_11589
3941	26s protease regulatory subunit 8-like
3943	metastasis-associated protein mta1-like
3944	tripeptidyl-peptidase 2-like
3945	regulator of g protein signaling
3946	protein unc-45 homolog b-like
3947	p160 coactivator fisc
3948	pou domain drifter cf-1a
395	polypyrimidine tract-binding protein 2
3951	sodium-coupled monocarboxylate transporter 2-like
3952	tetraspanin-7 (tspan-7) (transmembrane 4 superfamily member 2) (cell surface glycoprotein a15) (talla homolog) (cd231 antigen)
3953	2-oxoglutarate mitochondrial-like
3959	pyruvate mitochondrial-like
3960	zinc finger and BTB domain-containing protein 41-like

3961	zinc finger with ufm1-specific peptidase domain
3962	guanine nucleotide exchange factor
3963	upf0420 protein c16orf58 homolog
3965	growth factor independent 1 transcription repressor
3967	casein kinase i isoform epsilon
3971	ribonucleases p mrp protein subunit pop1-like
3972	No hits found
3974	amidase isoform 1
3975	golgi-associated pdz and coiled-coil motif-containing
3976	hypothetical protein KGM_14908
3977	kinesin-like protein kif3c-like
3978 [a]	mediator of rna polymerase ii transcription subunit 13-like
398	mau2 chromatid cohesion factor homolog
3983	26s protease regulatory subunit 7
3984	uridine-cytidine kinase-like 1-like isoform 1
3988	breast cancer anti-estrogen resistance protein 1-like
3990	tektin-1-like
3992	ras-related protein rab-8a-like isoform 2
3993	dual serine threonine and tyrosine protein kinase- partial
3994	protein emsy-like
3997	protein tob1-like
40	hypothetical protein KGM_02959
4000	trehalose 6-phosphate synthase
4003	protein mab-21-like
4009	prominin-like isoform 2
4010	sodium hydrogen exchanger 9 (nhe9)

4011 [a]	abl interactor 2
4012	cyclin fold protein 1
4013	kinesin heavy chain
4019	phosphatidylinositol-4-phosphate 3-kinase c2 domain-containing subunit beta-like
4027	fit family protein cg10671-like
4029	trithorax group protein
403	glycerol-3-phosphate acyltransferase
4032	von willebrand factor type egf and pentraxin domain-containing protein 1-like
4039	l-xylulose reductase
404 [b, c]	neither inactivation nor afterpotential protein g-like
4041	protein fam102b-like
4042	leucine-rich transmembrane protein
4044	lon peptidase n-terminal domain and ring finger protein 3-like
4045	annulin-like isoform 2
4046	attractin-like protein 1-like
4047	protein furry-like
4049	alanine--trna mitochondrial-like
4051	arsenite-resistance protein
4056	coiled-coil domain-containing protein 25
4058	26s proteasome non-atpase regulatory subunit 8
4059	zinc finger protein 729-like
406	polyribonucleotide nucleotidyltransferase mitochondrial-like
4060	hemolymph proteinase 5
4064	protein hu-li tai shao
4067	dedicator of cytokinesis protein 3-like

4068	chitin deacetylase 1
4069	wiskott-aldrich syndrome protein
407 [a, c]	g protein-coupled receptor kinase 1
4072	thyroid hormone receptor interactor 12
4077	inositol monophosphatase 1-like
4079	zinc finger protein 235-like
4082	ubiquitin specific protease
4083	cyclin-related protein fam58a-like
4084	beaten path ic
4089	oxysterol-binding protein 1a
4092	metabotropic glutamate receptor 4-like
4093 [b]	acetylcholine receptor subunit alpha-like
41	adenylate cyclase
4103	small ribonucleoprotein particle protein
4104	cation-dependent mannose-6-phosphate receptor-like
4105	ranbp-type and c3hc4-type zinc finger-containing protein 1 isoform x2
4107	transketolase-like protein 2-like isoform 1
4109	leucine carboxyl methyltransferase
411	octopamine receptor
4111	mitochondrial intermediate peptidase
4112	glutathione s-transferase sigma
4114	reticulon-1-like
4115	regulation of nuclear pre-mrna domain-containing protein 2-like
4116 [a]	ap-1 complex subunit gamma-1 (*AP-1gamma*)
4117	lysosomal acid phosphatase

4119	triple functional domain
412	pr domain zinc finger protein 14
4121	poly rna polymerase gld-2 homolog b-like
4123	putative protein sidekick precursor
4124	vesicle-fusing atpase 1-like
4126	hitcher protein
4129	ubiquitin carboxyl-terminal hydrolase calypso-like
413	lethal neighbour of tid
4130	golgi-specific brefeldin a-resistance factor
4135	pre-mrna-splicing factor spf27-like
4136	heat shock protein 40
4143	fact complex subunit ssrp1
4150	protein arginine n-methyltransferase 7-like
4151	5-aminolevulinate erythroid- mitochondrial-like
4152	retinoid-inducible serine carboxypeptidase-like
4156	serine threonine-protein kinase srpk1
4160	signal peptide peptidase
4162	valacyclovir hydrolase-like
4163	nadp transhydrogenase
4164	1-acyl-sn-glycerol-3-phosphate acyltransferase
4166	zinc finger protein 598-like
4167	eukaryotic translation initiation factor 3 subunit j
4168	baculoviral iap repeat-containing protein 6
4169	protein king tubby-like
417	nischarin-like
4172	histidine-rich membrane protein ke4 homolog 2-like

4174	hypothetical protein KGM_18349
4177 [b]	poly(adp-ribose) glycohydrolase 1-like isoform 1
4181	rna polymerase ii-associated protein 1-like
4182	nadh dehydrogenase
4183	serine threonine-protein kinase sik3-like
4188	transmembrane protein 222
419	interleukin enhancer-binding factor 2 homolog
4192	corepressor interacting with rbpj 1-like
4193	voltage-dependent l-type calcium
4194	thap domain-containing protein 9
4199	adam 19
420	sarcolemmal membrane-associated
4201 [b]	hypothetical protein KGM_22676
4209	hypothetical protein KGM_09294
4213	low quality protein: midasin-like
4214	katanin p80 wd40-containing subunit b1
4215	trafficking protein particle complex subunit 2-like
4218	zinc finger protein 91-like
4219	ubiquitin-associated protein 1-like isoform x3
4222	mitochondrial thiamine pyrophosphate carrier-like
4223	splicing factor 3a subunit 1-like
4224	protein prenyltransferase alpha subunit repeat-containing protein 1
4225	60s ribosomal export protein nmd3-like
4226	atp-dependent rna helicase ddx42-like
4227	steroid dehydrogenase
423	pv-fam-d protein

4230	proto-oncogene c-fos-like
4231	e3 ubiquitin-protein ligase ubr2-like
4233	neuroendocrine convertase 2-like
4237	aspartate aminotransferase
4238	translocation protein sec62
4239	nucleolysin tiar-like
424	mitochondrial aldehyde dehydrogenase
4240	gmp reductase 1-like
4244	alpha-1,3-mannosyl-glycoprotein 4-beta-N-acetylglucosaminyltransferase B-like
4247	putative protein sidekick precursor
4248	tryptophanyl-trna synthetase
4249	integrase core domain protein
425	myotubularin-related protein 3-like
4250	low quality protein: tuberin-like
4254	peroxisome assembly protein 12
4255	single-stranded dna-binding protein 3-like
4258	peroxisomal leader peptide-processing protease-like
4259	transcription initiation protein spt3 homolog
426	tyrosine-protein phosphatase non-receptor type 9-like
4260	calmodulin-like protein 4-like
4267	protein toll-like
427	dynamin-like 120 kda mitochondrial-like
4270 [a]	tyrosine-protein kinase abl-like
4272	ataxin-7-like protein 1 isoform x2
4274	upf0378 protein kiaa0100-like

4276	hypothetical protein KGM_01858
4278	protein lsm14 homolog a-like isoform x1
4279	homeobox protein cut
4282	hemolymph proteinase 9
4284	f-box wd repeat-containing protein 1a-like
4290	max dimerization-like protein
4292	oxidative stress-responsive serine-rich protein 1-like isoform x2
4294	ubiquitin carboxyl-terminal hydrolase 20 isoform x1
4295	suppressor of g2 allele of skp1 homolog
4296	macrophage mhc class i receptor 2-like protein
4297	u8-agatoxin-ao1a-like isoform 2
43	carbohydrate sulfotransferase 11-like
430	ccr4-not transcription complex subunit 3
4302 [a]	protein abrupt-like
4303	signal sequence receptor beta
4304 [a]	ras association domain-containing protein 8-like
4305	translation initiation factor eif-2b subunit epsilon
4309	nucleoporin nup211-like
431	flap endonuclease-1
4312	zinc finger protein 888 isoform x2
4313	tetratricopeptide repeat protein 21b
4317	condensin complex subunit 3-like
4318	nadh dehydrogenase
4319	abhydrolase domain-containing protein 3-like
4320	peptidyl-prolyl cis-trans isomerase-like 4-like
4322	phosphoglycerate kinase

4323	hypothetical protein KGM_09036
4324	nmda receptor-regulated protein 2
4325	a kinase anchor protein
4326	potassium voltage-gated channel protein eag-like
433	putative tuberous sclerosis 2 isoform 3
4331	sodium chloride cotransporter isoform a
4332	catenin alpha-like
4333	glutathione s-transferase
4336	telo2-interacting protein 1 homolog
4338	bifunctional 3'-phosphoadenosine 5'-phosphosulfate synthase-like isoform X2
4340	probable ubiquitin carboxyl-terminal hydrolase faf-x
4342	rhythmically expressed gene 2
4343	nuclear pore membrane glycoprotein 210-like
4345	xaa-pro dipeptidase
4347	carboxylesterase
4349	disco-interacting protein 2-like
435	coatomer subunit alpha
4351	u1 small nuclear ribonucleoprotein c-like
4352	voltage-dependent calcium
4353	pre-mrna-splicing factor rbm22-like
4354 [a]	vacuolar protein sorting-associated protein 28 homolog
4356	pol-like protein
4364	dna (cytosine-5)-methyltransferase -like
4365	rab gtpase-binding effector protein 1-like
4367	transportin-3

4368	dorsal-ventral patterning protein sog-like
4369	phosphoinositide 3-kinase regulatory subunit 4
4371	polypeptide n-acetylgalactosaminyltransferase 5-like
4373	noki protein
4378	vacuolar protein sorting-associated protein 11 homolog
4379	tho complex subunit 6 homolog
4380	ddb1- and cul4-associated factor 8-like
4381	p94-like protein
4385	hypothetical protein KGM_12108
4386	leucyl-trna synthetase
4389	telomere-associated protein rif1-like
439	cg13189-pa
4391	serine threonine-protein kinase ulk2-like
4394	long-chain-fatty-acid--CoA ligase 4-like isoform X2
4396	wd repeat-containing protein 44-like
4397	kinesin-like protein klp68d-like
4399	vacuolar protein sorting-associated protein vta1 homolog
4401	delta -sterol reductase
4402	fatty acyl- reductase cg5065-like
4405	uncharacterized oxidoreductase dhs-27-like
4409	hypothetical protein KGM_12609
441 [a, b]	orthodenticle
4412	monocarboxylate transporter 14
4413	furin-like protease isoforms 1 1-x 2-like
4416	ankyrin repeat and btb poz domain-containing protein 2-like
4417	ddb1- and cul4-associated factor 12-like

4419	thioredoxin domain-containing protein 9
442	cg2467 cg2467-pa
4420	tyrosine aminotransferase
4421	proteasome activator complex subunit 4-like
443	hexokinase hkdc1-like
4430 [a]	zinc finger protein 643-like
4431	ef hand domain protein
4433	integrator complex subunit 4
4434	hect type e3 ubiquitin ligase
4435	flavin-dependent monooxygenase
4438 [a]	low quality protein: wd repeat and fyve domain-containing protein 3-like
4440	calcium-transporting atpase sarcoplasmic endoplasmic reticulum type-like
4445	u4 tri-snrnp-associated protein 1
4446	cd63 antigen
4447	zinc finger and scan domain-containing protein 10-like isoform x3
445	c2 domain-containing protein 5-like
4450	phosphoribosylformylglycinamidine synthase-like
4451	homeobox protein prospero
4454	connectin-like
4458 [a]	adp-ribosylation factor 2-like
446	erythrocyte membrane-associated giant protein antigen 332
4460	netrin receptor unc5b-like
4462	geranylgeranyl transferase type-1 subunit beta-like
4463	nucleosome assembly protein isoform 2
4467	potassium channel subfamily k member 18-like

4468	cg6325 cg6325-pa
4469	la-related protein 1-like
447	cysteine synthase
4470	rho gtpase-activating protein 21
4472	nicotinate phosphoribosyltransferase-like isoform x2
4474	head-specific guanylate cyclase-like
4475	mitochondrial ribosomal l23
4476	e3 ubiquitin-protein ligase siah1-like
4477	cg31997 cg31997-pa
4478	sex-lethal homolog isoform x5
4479	dual specificity tyrosine-phosphorylation-regulated kinase 2-like
4482	nuclear hormone receptor
4483	gtpase-activating protein and vps9 domain-containing protein 1-like
4484	domain-containing protein 7
4485	nuclear hormone receptor ftz-f1-like
4487	zinc finger protein 99
4488	protein o-mannosyltransferase 1-like
4492	extradenticle
4493	leucine-rich repeat and calponin-like proteiny domain-containing protein 3
4494	baculoviral iap repeat-containing protein 6-like
4496	zinc finger ccch domain-containing protein 11a
4498	uncharacterized protein LOC101742879
450	hypothetical protein KGM_15734
4502	wd repeat-containing protein 81-like
4509	neuropeptide y receptor type 2

4510	mrna-capping enzyme-like
4511 [a]	glutathione s-transferase omega 1
4512	atp-binding cassette sub-family g member 1-like
4513	probable peroxisomal acyl-coenzyme a oxidase 1-like
4516	peroxisomal n1-acetyl-spermine spermidine oxidase
4517	ommochrome-binding protein precursor
452	protein son of sevenless-like
4521	protein lingerer
4524	rh-like protein
4526	sialyltransferase
4527	fibrillarin, partial
4532	hypothetical protein KGM_10185
4535	ring finger and spry domain-containing protein 1-like isoform x1
4536 [b]	isoform d
4538	cullin
4539	ubiquitin-like domain-containing ctd phosphatase 1-like
454	putative f-spondin
4540	carnitine o-palmitoyltransferase mitochondrial-like
4544	uncharacterized protein LOC101746500
4547	transformation transcription domain-associated protein
4549	mediator of rna polymerase ii transcription subunit 16-like
455	kazrin-like isoform x1
4551	adhesion related transmembrane
4553	g-protein coupled receptor
4554	kazal-type inhibitor
4556	tetratricopeptide repeat protein 17

4562	frequenin isoform c
4563	similar to CG2519
4564	erythroid differentiation-related factor 1-like
4565	ribosomal protein s24
4567	nitric oxide-associated protein 1-like
4568	hypothetical protein KGM_14611
4572	spectrin beta brain 4-like
4573	nad-dependent adp-ribosyltransferase sirtuin-4-like
4574	glycosylphosphatidylinositol anchor attachment 1
4578	bromodomain-containing protein 7
4579	probable phospholipid-transporting atpase id-like
4581	sensory neuron membrane protein 2
4582	hypothetical protein KGM_15485
4583	xanthine dehydrogenase
4584	hemolymph proteinase 8
4585	atp-dependent clp protease atp-binding subunit clpx-mitochondrial-like
4586	ras-related gtp-binding protein a-like
4588	heat repeat-containing protein 5b-like
459	antennal esterase cxe11
4592	mitochondrial sodium hydrogen exchanger 9b2-like
460	rho gtpase-activating protein 190-like
4601	aldehyde dehydrogenase
4602	uncharacterized protein LOC101738037
4604	lipoma hmgic fusion partner-like 2
4606	activin receptor type-2b-like

4607	translocation associated membrane protein
4611	lutropin-choriogonadotropic hormone receptor
4613	ubiquitin carboxyl-terminal hydrolase 34-like
4614	5-oxoprolinase-like isoform x1
4615	e1a binding protein p400
4616	pyruvate dehydrogenase
4617	bai1-associated protein 3-like
4618	probable rna polymerase ii nuclear localization protein slc7a6os-like
4619	acetyltransferase 1
462	tbc1 domain family member 13-like
4621 [a]	kruppel homolog 1
4622	glutathione s-transferase omega class
4624	histone-lysine n-methyltransferase
4625	atpase n2b-like
463	phosphatidylserine receptor
4630	fork head domain transcription factor partial
4637	probable nuclear hormone receptor hr38
4638	e3 ubiquitin-protein ligase ubr2-like
4643	uncharacterized protein LOC101744206
4644	autophagy-related protein 16-1-like
4645	fatty acid transport protein
4649	golgi reassembly-stacking protein 2-like
4650	solute carrier organic anion transporter family member 4a1-like
4651	cell cycle regulator mat89bb homolog
4652	gpi transamidase component pig-s-like

4654	hypothetical protein KGM_21537
4658	thioredoxin domain-containing protein 15-like
4659	ubiquitin specific protease
4660	ischemia reperfusion inducible protein
4665	nf-kappa-b inhibitor zeta
4667	calcium uniporter mitochondrial
4669	zinc finger protein 91
467	probable rho gtpase-activating protein cg5521-like
4679	tmem57 partial
4680	ets-related transcription factor elf-3
4683	dna-directed rna polymerase ii subunit rpb9-like
4684	caax prenyl protease 1 homolog
4685	connectin-like
4687	acidic nucleoplasmic dna-binding protein 1
4688	protein arginine n-methyltransferase 3-like
4690	alpha-2-macroglobulin receptor-associated
4692	reversion-inducing cysteine-rich protein with kazal motifs-like
4693	magnesium transporter nipa2-like
4694	cdkal1-like protein
4695	septin-1-like
4696	furin-like convetase
4699	glutathione s-transferase omega 1
47	alcohol dehydrogenase
470	dnaj homolog subfamily a member 1-like
4701	selenium-binding protein
4704	polycomb protein suz12

4705	dual specificity protein kinase pom1-like isoform x4
4709	gata-binding factor a
471	hypothetical protein KGM_12191
4710	carotenoid-binding protein
4715	transportin-1
4717	ankyrin repeat protein
4718	bleomycin hydrolase
4719	low quality protein: merlin-like
472	replication protein a 32 kda subunit
4721	cAMP-specific 3',5'-cyclic phosphodiesterase, isoform I-like
4722	mitochondrial import receptor subunit tom20 homolog
4723	adenylate cyclase
4725	rna polymerase ii second largest subunit
4726	sox domain-containing protein dichaete-like
4729	tfiih basal transcription factor complex helicase xpb subunit
4732 [b]	seminal fluid protein hacp034
4733	galactokinase 1
4734	hypothetical protein KGM_17470
4736	anaphase-promoting complex subunit 2
4737	coatomer subunit beta
474	multiple inositol polyphosphate phosphatase 1-like
4740	protein disulfide-isomerase a6-like
4741	tnf receptor-associated factor 6-like
4742	nuclear rna export factor 1
4748	nhp2-like protein 1

475	methyl-CpG-binding domain protein 5
4751	alpha-tocopherol transfer
4752	39s ribosomal protein mitochondrial
4753	ccr4-associated factor
4754	stromal membrane-associated protein 1-like
4755	colorectal mutant cancer protein
4756	dna-binding protein ewg
4757	protein fam38b
4758	fragile x mental retardation syndrome-related protein 1
4759	monocarboxylate transporter
4763	nudc domain-containing protein 1
4765	atp-dependent rna and dna helicase
4772	hspb1-associated protein 1-like
4774	vacuolar protein sorting-associated protein
4775	thap domain-containing protein 2
4777	netrin receptor unc5c-like
4779	hypothetical protein KGM_20226
4781	hypothetical protein KGM_13282
4782	c3 and pzp-like alpha-2-macroglobulin domain-containing protein 8
4784	exportin-2-like
479	death-associated protein kinase isoform c
4790	leucine-rich transmembrane protein
4792	chromodomain-helicase-dna-binding protein mi-2 homolog
4798	e3 ubiquitin-protein ligase ubr7-like
48	endonuclease-reverse transcriptase -e01

4801	tyrosine-protein kinase csk-like
4802	surfeit locus protein 1 isoform x2
4803 [a]	probable ubiquitin carboxyl-terminal hydrolase faf-like
4804	6-phosphogluconolactonase-like
4807	protein furry-like
481	endoribonuclease dcr-1
4814	g-protein coupled receptor moody-like
4818	protein disulfide isomerase
4819	carboxypeptidase inhibitor
4823 [b]	hypothetical protein KGM_12063
4824	cg7781 cg7781-pa
4825	No hits found
4826	putative cxpwmw03
483	chromosome region maintenance protein 5 exportin
4831	hypothetical protein KGM_12603
4832	chromosome segregation protein smc
4835	centrosomal protein 350kda
4836	long-chain fatty acid transport protein 4-like
4838	tyrosine-protein phosphatase non-receptor type 23
4840	cg10206-pa
4842	pollen-specific leucine-rich repeat extensin-like protein 2-like
4843	fatty acyl- reductase cg8306-like
4844	cg5098-like protein
485	serine threonine-protein kinase pak 1-like isoform x1
4850	merozoite-related surface protein 5
4852	gpcr class b orphan receptor 1 (agap009007-pa)

4853	denn domain-containing protein 5b-like
4854	active breakpoint cluster region-related
4855	hypothetical protein KGM_06111
4858	probable multidrug resistance-associated protein lethal 03659-like
486	scramblase isoform b
4860	FancJ-like protein
4862	defective proboscis extension response
4863	dna-binding protein rfx7
4864 [b]	hypothetical protein KGM_11896
4866	cg8965 cg8965-pa
4867	uncharacterized protein LOC101746816 isoform X1
4868	sugar-free isoform c
4871	s-adenosylmethionine decarboxylase proenzyme-like
4872	membrane-bound transcription factor site-1 protease isoform x1
4875	atpase aaa
4877	gtp-binding nuclear protein ran
488	juvenile hormone esterase binding protein
4880	zinc finger protein zfy
4882	pre-mrna-splicing factor 18-like
4883	ribonucleoside-diphosphate reductase small chain
4884	serine protease
4885	potassium channel subfamily k member 9
4886	transcriptional repressor protein yy1-like isoform 1
4889	sumo-1 activating enzyme
4890	wd repeat-containing protein 48
4891	swi snf complex subunit smarcc2-like

4892	diminutive
4894	krab box and zinc finger c2h2 type domain containing protein
4900	eukaryotic initiation factor 4a-iii-like
4903	rotatin-like isoform x2
4905	las1-like protein
4907	serine hydroxymethyltransferase
4908	erythrocyte membrane-associated giant protein antigen 332-like protein
4909	hypothetical protein KGM_09759
491	hypothetical protein KGM_18603
4915	ribosome biogenesis protein bop1
4920	h aca ribonucleoprotein complex subunit 1-like
4926	ccr4-not transcription complex subunit 10 isoform x2
4927	down syndrome cell adhesion molecule-like protein dscam2-like
4929	enolase-phosphatase e1-like
493	plastin-3-like isoform x2
4930	dna helicase ino80-like
4933	uncharacterized protein LOC101744603
4934	zinc finger protein
4935	vrp1 verprolin-related protein
4937	trna-splicing ligase homolog
494	forkhead box protein o-like
4942	ubiquitin carboxyl-terminal hydrolase 10
4945	chitin synthase
4946 [b]	hemocyte protein-glutamine gamma-glutamyltransferase-like
4947 [a]	zinc finger protein ccch domain-containing

4948	low quality protein: cartilage oligomeric matrix
4949	mitochondrial ribosomal protein l1
495	zinc finger protein 347
4950	glutamate-gated chloride channel
4952	peptidyl-prolyl cis-trans isomerase d
4954	dopamine transporter
4956	hypothetical protein KGM_21980
4957	peroxisomal membrane protein 70
4958	serine threonine-protein phosphatase 4 regulatory subunit 3-like
4960	at-rich interactive domain-containing protein 5b
4961	lysosomal pro-x carboxypeptidase-like
4964	apolipoprotein o-like
4965	histone
4966	atp synthase
4967	probable ribosome production factor 1-like
497	peptidyl-prolyl cis-trans isomerase e
4973	ras-related protein rab-3-like
4974	collagen type iv alpha-3-binding
4975	multiple c2 and transmembrane domain-containing protein 1-like
4976	glutathione peroxidase
4978	protein bicaudal d-like
498	zinc transporter 1-like
4980	methylosome subunit picln
4982	vacuolar protein sorting-associated protein 11 homolog
4983	transcription elongation factor spt5-like
4985	importin-13-like

4986	lethal malignant brain tumor-like protein 3-like
4987	translocator protein
4988	sodium solute symporter
4989	truncated carboxylesterase
4992	ly-6 neurotoxin superfamily member 1
4993 [a]	protein tiptop-like
4995	probable glutamate--tRNA ligase, mitochondrial-like
4998	seipin-like
5	sugar transporter
500	f-box only protein 11-like
5002	transcription factor
5003	vinc_drome ame: full=vinculin
5004	yth domain family protein 1-like
5005	gpi transamidase component pig-s-like
5006	pseudouridylate synthase
5007 [a]	mediator of rna polymerase ii transcription subunit 12
5008	f-box only protein 11-like
501	cg8180 cg8180-pa
5011	probable dynactin subunit 2-like
5012	carbonyl reductase
5013	hypothetical protein KGM_18717
5014	leucine rich repeat protein
5015	queuine trna-ribosyltransferase
5017	cytoplasmic dynein 2 heavy chain 1
5018	dual specificity protein phosphatase 12-like
5019	dna replication licensing factor mcm5

5023	uncharacterized protein LOC101745243
5024	hypothetical protein KGM_18561
5031 [b]	hypothetical protein KGM_17721
5034	probable ribosome biogenesis protein rlp24-like
5035	vasa intronic gene
5037	ubiquitin-conjugating enzyme e2 d3 isoform x1
5038 [b]	organic cation transporter
504	oxysterol-binding protein
5040	myosin-ib-like isoform x2
5042	ribosomal protein l27
5043	sterol regulatory element-binding protein 1
5046	mitochondrial nadh:ubiquinone oxidoreductase
5048	tata box-binding protein 1-like
5051	rho rac-interacting citron kinase
5052	wd repeat-containing protein 19-like
5053	facilitated trehalose transporter tret1-like
5055	rab gtpase-binding effector protein 1-like
5056	b-cell receptor-associated protein 31-like
5059	rna exonuclease nef-sp-like
506	cyclin h
5062	angiotensin converting enzyme
5064 [b]	nmda-type glutamate receptor 1
5066	integrator complex subunit 11-like
5068	amp dependent coa ligase
5070	hypothetical protein KGM_12969
5071	condensin-2 complex subunit d3-like

5072	interferon regulatory factor 2-binding protein 2-like
5074	aquaporin
5075	transmembrane protein 8a-like
5076	e3 ubiquitin-protein ligase mylip-like
5078	hypothetical protein KGM_00004
508 [b]	morn repeat-containing protein 4-like
5081	No hits found
5084 [b]	transcription initiation factor tfiid subunit 12-like
5087	aldo-keto reductase
5088	zinc finger protein 569-like isoform x3
509	flocculation protein flo11-like
5092	probable methyltransferase-like protein 15 homolog
5093	low density lipoprotein receptor adapter protein 1-like
5096	bm8 interacting protein 2d-2
5098	dna damage-binding protein 1-like
5100	protein sidekick
5101	ninjurin a
5103	map kinase-activating death domain
5104 [a]	voltage-dependent anion-selective channel isoform 1
5106	antennal esterase cxe5
5107	citrate synthase
5108	low quality protein: integrator complex subunit 3 homolog
5110	h+ transporting atp synthase subunit d
5111	transmembrane protein 43 homolog
5112	h+ transporting atp synthase gamma subunit
5113	ornithine decarboxylase

5115	atp-binding cassette sub-family b member mitochondrial-like
5120	monocarboxylate transporter
5122	hypothetical protein KGM_06309
5123	fmrfamide receptor
5124	putative guanyl-nucleotide exchange factor
5126	wd40 repeat-containing protein smu1-like
5128	protein ndrg3-like
513	hypothetical protein KGM_05643
5130	fad oxidoreductase
5131	e3 ubiquitin-protein ligase listerin
5132 [a]	afadin-like isoform x2
5136	nadph:adrenodoxin mitochondrial-like
5138	s1 rna-binding domain-containing protein 1-like
5140	viral a-type inclusion protein
5141	rna-binding protein 11-like
5143	zinc finger protein 598-like
5144	fatty-acyl reductase 6
5145	homocysteine s-methyltransferase isoform 1
5149	probable aminopeptidase npepl1
5150	noc2l protein
5151	peroxiredoxin 1-like
5152	myelin transcription factor 1-like protein
5153	groucho protein
5154	diphthine--ammonia ligase-like isoform x1
5157	viral a-type inclusion protein
5159	prostamide prostaglandin f synthase

516	phosphoglucose mutase
5160	roundabout homolog 2-like
5161	lysosomal thioesterase ppt2 homolog
5163	protein sidekick-like
5165	hypothetical protein KGM_08018
5166	potassium channel subfamily k member 18-like
5168	cg1785-like protein
5169	wnt11 protein
517	glycosyl-phosphatidyl-inositol-anchored protein
5171	polycomb protein sfmbt
5172	acyl- synthetase family member mitochondrial-like isoform 2
5173	hypothetical protein KGM_10611
5175	potassium channel kcnq
5177	39s ribosomal protein mitochondrial
5178	secreted protein
5180	cytochrome c1
5181	ribosomal protein p0
5184	hexamerin 2 beta
5185 [a]	hypothetical protein KGM_09342
5186	s-adenosyl-l-homocysteine hydrolase
5188	atpase inhibitor mai- mitochondrial-like
519	protein toll
5191	nuclear nucleic acid-binding protein c1d-like
5193	synaptic vesicle glycoprotein 2b-like
5194 [b]	down syndrome cell adhesion molecule-like protein dscam2-like
5196	mucin-2-like

5197	hypothetical protein KGM_16722
5198	39s ribosomal protein mitochondrial-like
520	hypothetical protein KGM_09033
5201	adenylate cyclase
5203 [a]	dally- isoform a
5204	phosphoribosylformylglycinamidine synthase-like
5205	splicing factor 1-like
5209	vacuolar atp synthase 21 kda proteolipid subunit
5211	facilitated trehalose transporter tret1-like
5213	mitochondrial import inner membrane translocase subunit tim22
5214	transmembrane protein 87a-like
5219	dna cross-link repair 1a protein
5221	transformation transcription domain-associated protein
5224	fad-dependent oxidoreductase domain-containing protein 1-like
5225	alkaline tissue-nonspecific isozyme-like
5226	hypothetical protein KGM_20846
5227	hypothetical protein KGM_18075
5228	single-stranded dna-binding mitochondrial
5229	1-acylglycerol-3-phosphate acyltransferase
5230	cuticular protein analogous to peritrophins 3-e
5233	helicase arip4
5236	f-box lrr-repeat protein 6-like
5237	cytochrome p450
5238	dual serine threonine and tyrosine protein kinase
524	serine protease p54
5241 [b]	homeobox protein arx

5242	zinc transporter 8-like
5243	glycerol-3-phosphate acyltransferase
5246	hypothetical protein KGM_20043
5247	serine threonine-protein kinase smg1-like
5248	neuroendocrine protein 7b2
5252	zgc:173726 protein
5255	udp-n-acetylglucosamine--peptide n-acetylglucosaminyltransferase 110 kda subunit-like
5256	dual specificity protein phosphatase cdc14a-like
5257	peptidyl-prolyl cis-trans isomerase 5
5259	endothelin-converting enzyme 1
5260	small integral membrane protein 14
5262	aldo-keto reductase
5263	cytochrome b-c1 complex subunit mitochondrial-like
5266	mannose-1-phosphate guanyltransferase beta-like
527	transcription factor sox-5-like
5270	protein 60a-like
5272	formin-like protein cg32138-like
5273	tyrosine-protein kinase shark-like
5274	sphingosine-1-phosphate phosphatase 1-like
5275	trna mitochondrial-like
5278	heat shock protein 70
5279	monocarboxylate transporter 10-like
5280	thioredoxin-related transmembrane protein 1-like
5282	low quality protein: protein unc-80 homolog
5284	max-binding protein mnt-like isoform x1

529	gelsolin precursor
5290	serine protease inhibitor partial
5291	u6 snrna-associated sm-like protein lsm4
5293	serine threonine-protein kinase gd17699-like
5294	dna damage-binding protein 1
5295	lethal essential for life l2efl
5296	partitioning defective 3 homolog
5297	groucho protein
5298	mrna cap-binding protein eif4e
5299	hypothetical protein KGM_00342
530	dyslexia-associated protein kiaa0319-like
5300	serine-threonine kinase receptor-associated protein
5302	zinc iron transporter
5304	d-3-phosphoglycerate dehydrogenase-like
5308	peptidyl-prolyl cis-trans isomerase-like 2-like
5310	small g protein signaling modulator 3 homolog
5311	pumilio homolog 1-like
5312	metabotropic glutamate receptor 2-like
5313	eukaryotic translation initiation factor 4e transporter
5317	general receptor for phosphoinositides 1-associated scaffold protein
532	uncharacterized protein LOC101742398
5321	zinc finger protein 161 homolog
5322	hypothetical protein KGM_00374
5325	phosphoglycerate mutase family member 5
5326	hypothetical protein KGM_06006

5327	14-3-3 epsilon protein
533	2-deoxyglucose-6-phosphate phosphatase
5330	s-phase kinase-associated protein 1
5331	sortilin-related receptor-like
5333	10 kda heat shock mitochondrial-like
5336	serine protease
5337	n -dimethylguanosine trna methyltransferase
5338	nadh dehydrogenase
534 [b]	defective proboscis extension
5344	acetylcholinesterase 1
5345	eukaryotic translation initiation factor 3 subunit i-like
5346	udp-glycosyltransferase ugt33d6
535	n-acetylgalactosaminyltransferase 6-like
5350	cysteinyl-trna synthetase
5352	low quality protein: phosphopantothenate--cysteine ligase-like
5356	lipoma preferred partner lpp
5357	dna fragmentation factor-related protein isoform a
5358	gtpase-activating protein
5359	c2 domain-containing protein 5-like
536 [b]	homeobox protein nkx- -like
5360	hypothetical protein KGM_02740
5362	serine proteinase-like protein 1
5366	signal recognition particle 19 kda
537	rho rac-interacting citron kinase
5371	transmembrane protein 173

5372	ww domain-containing adapter protein with coiled-coil-like isoform x2
5374	dnaj homolog subfamily c member 3-like
5375	chitinase
5377	ras-like protein 2-like
5378	40s ribosomal protein sa-like
5380	hypothetical protein KGM_00432
5383	neuropeptide receptor a15
5384	acylphosphatase-1-like
5385	kinase d-interacting substrate of 220 kda-like
5386	exostosin-1-like isoform 1
539	protein sly1 homolog
5390	nose resistant to fluoxetine protein 6-like
5391	methyltransferase-like protein 22-like
5395	calcium homeostasis endoplasmic reticulum isoform x1
5396	hd domain-containing protein 2-like
5398	ubiquitin carboxyl-terminal hydrolase-like
5399	ring-box protein 1a-like
54	g protein pathway suppressor 1
540	f-box only protein 9-like
5400	integrin beta subunit
5401	chemosensory protein 2 partial
5403	hypothetical protein KGM_01472
5404	translational activator gcn1
5405	chromodomain-helicase-dna-binding protein 7
5408	suppressor of lurcher protein 1-like

5410	high mobility group protein
5412	diacylglycerol kinase epsilon-like
5413	aminopeptidase n
5414	general transcription factor 3c polypeptide 5 isoform 2
5417	active breakpoint cluster region-related
5419	endonuclease iii-like protein 1
542	udp-glucose:glycoprotein glucosyltransferase
5421	protein wnt-5b-like
5423	hypothetical protein KGM_04852
5424	cytospin-a-like isoform x2
5427	vacuolar protein sorting-associated protein 8 homolog
5429	serpin 12 non-inhibitory serine protease inhibitor
5432	mediator of rna polymerase ii transcription subunit 25
5435	n6-adenosine-methyltransferase 70 kda subunit
5436	neuroblastoma-amplified protein
5437	ap-3 complex subunit delta-like
5438	nadh dehydrogenase
5440	liprin- isoform a
5445	No hits found
545	ribosomal protein l4
5454	pleckstrin homology domain-containing family f member 2-like
5455	probable multidrug resistance-associated protein lethal 03659-like isoform x1
5456	aldo-keto reductase
5457	rna-binding protein nob1-like
5459	liprin-beta-2-like isoform x1

546	odorant-binding protein partial
5462	inhibitor of apoptosis 2 protein
5467	nadh dehydrogenase
547	cyclohex-1-ene-1-carboxyl- hydratase
5470	elongation factor 1 beta
5472	calponin transgelin
5473	odorant-binding protein
5474	leucine-rich repeat-containing protein 20
5477	endoplasmin-like precursor
5478	circadian locomoter output cycles protein kaput
5479	dcn1-like protein 3-like
5480	vacuole membrane protein 1 isoform x1
5481	dna replication licensing factor mcm6
5483	alpha-taxilin isoform x2
5484	glutathione s-transferase c-terminal domain-containing protein homolog
5485	ecdysone oxidase
5487	sid1 transmembrane family member 1
5489	carbon catabolite repressor protein
5492	protein hid1-like isoform x2
5495	la-related protein 1-like
5496	tribbles homolog 2-like
550	kinase d-interacting substrate of 220 kda-like
5500	unkown protein
5503	u4 u6 small nuclear ribonucleoprotein prp31-like
5504	endophilin-a-like isoform x2

5507	ral GTPase-activating protein subunit beta-like
5511	protein toll-like
5512	tata-box-binding protein
5517	mitochondrial nadh-ubiquinone oxidoreductase 9 kda subunit-like protein
5518 [b]	lachesin-like (*DIP-alpha*)
5519	neurotrimin- partial
552 [b]	hypothetical protein KGM_19577
5520	growth arrest and dna damage-inducible proteins-interacting protein 1-like
5521	u5 small nuclear ribonucleoprotein 40 kda
5522	hypothetical protein KGM_14433
5523	acyl- z9 desaturase
5524	afadin-like isoform x3
5525	nascent polypeptide-associated complex subunit muscle-specific form-like
5527	cationic amino acid transporter
5532	26s protease regulatory subunit 4-like
5533	tetraspanin 29fb
5534	putative FL2D protein
5537	eukaryotic translation initiation factor 4h
554	bhlhzip transcription factor max bigmax
5541	chemosensory protein
5542	protein extra bases-like
5545	lipoyltransferase mitochondrial-like
5546	endothelial differentiation-related factor 1 homolog
5548	tensin

5549	atpase aaa
5551 [b]	down syndrome cell adhesion molecule-like protein dscam2-like
5554	nadh dehydrogenase
5555	hypothetical protein KGM_10100
5561	nuclear pore complex protein nup155
5563	actin-binding rho-activating
5564	cytoskeleton-associated protein 5
5565	oxysterol-binding protein 1
5566	nadh-ubiquinone oxidoreductase fe-s protein 2
5569	aldehyde dehydrogenase
557	tenascin-like isoform x2
5570	26s proteasome non-atpase regulatory subunit 9
5571	probable actin-related protein 2 3 complex subunit 2-like
5575	protein transport protein sec24c-like
5578	g t mismatch-specific thymine dna
5580	protein prickle-like
5581	hypothetical protein KGM_22463
5582	cgi-01 protein
5583	nuclear cap-binding protein subunit 1-like
5584	upf0468 protein c16orf80 homolog
5585	serine protease inhibitor 5
5586	saccharopine dehydrogenase domain-containing protein
5588	zinc finger fyve domain-containing protein 26
5589	visual system homeobox 2-like
5592	vacuolar protein sorting
5593	ankyrin repeat and fibronectin type-iii domain-containing protein 1

5596	congested-like trachea
5597	low quality protein: ca(2+) calmodulin-responsive adenylate cyclase-like
5598	beta-sarcoglycan
5599	serine threonine protein kinase
56	protein tssc1-like
5600	ddb1- and cul4-associated factor 10 homolog
5605	aminopeptidase n
5606	nedd8-conjugating enzyme ube2f-like
5607	guanine nucleotide-binding protein subunit beta-like protein 1-like
5609	protein n-terminal asparagine amidohydrolase-like
5611	glutathione s-transferase omega 1
5613	signal sequence receptor
5614	c-Cbl-associated protein isoform A
5617	eukaryotic translation initiation factor theta subunit
5618	p23-like protein
5619	serine arginine repetitive matrix protein 2-like isoform x2
5621	acidic leucine-rich nuclear phosphoprotein 32 family member b-like isoform x3
5623	ribosomal protein l28
5628	hemicentin-1-like isoform x1
563	probable protein phosphatase cg10417-like
5630	hemolymph proteinase 16
5632	hypothetical protein KGM_04624
5639	gpn-loop gtpase 3-like
564	hypothetical protein KGM_20399
5642	protein dgcr14 homolog

5644	echinoderm microtubule-associated 1-like isoform x5
5646	g patch domain and ankyrin repeat-containing protein 1 homolog
5647	transmembrane and coiled-coil domains protein 1-like
5649	hypothetical protein KGM_11032
565	signal recognition particle 72 kda protein
5650	succinate dehydrogenase
5653	loc733328 protein
5654	serine arginine repetitive matrix protein 5-like isoform x1
5655	exocyst complex component 4
5657	pogo transposable element with znf domain
5658 [a]	e3 ubiquitin-protein ligase mind-bomb-like
566	t-box protein h15-like
5660	aconitate mitochondrial-like
5661	coatomer subunit beta
5663	adenylate cyclase
5664	udp-glucosyltransferase precursor
5665	leucine-rich repeat-containing protein ddb_g0290503-like isoform x2
5666	uncharacterized protein LOC101739175, partial
5667	protein sco1 mitochondrial-like
5670	and pleckstrin domain-containing protein 2
5671	abhydrolase domain-containing protein 11-like
5675	chondroitin sulfate proteoglycan 4
5677	peroxidase-like isoform x1
5678	hsp70-binding protein 1-like
5679	hypothetical protein KGM_14526

5681	ubiquitin-fold modifier-conjugating enzyme 1-like
5682	hypothetical protein KGM_16828
5686	solute carrier family 25 member 35-like
5690	control protein hctl023
5692	nadph:quinone reductase
5693	atpase family aaa domain-containing protein 3
5697	dipeptidyl peptidase 4
5698	hypothetical protein KGM_19061
570	wd repeat domain 35
5701	inactive pancreatic lipase-related protein 1-like
5702	fumarylacetoacetase
5703	ribonuclease p mrp subunit
5705	dna-directed rna polymerase ii subunit rpb3-like
5706	probable nucleolar gtp-binding protein 1-like
5711	amine oxidase
5712	poly -binding protein 3-like
5716	ankyrin unc44
5719	secernin-3
572	ubiquitin domain-containing protein 2-like
5720	protein asteroid-like
5721	hypothetical protein KGM_20527
5724	cop-coated vesicle membrane protein p24
5725	atp-binding cassette sub-family e member 1
5726	phd finger protein 12-like
5727	hypothetical protein KGM_08056
5729	carboxypeptidase b-like

5730	growth arrest-specific protein 2-like
5731 [a]	probable ubiquitin carboxyl-terminal hydrolase faf-x
5732	signal recognition particle 54 kda protein
5733	bindin, partial
5735	apolipoprotein of lipid transfer particle-iii
5736	cytosolic fe-s cluster assembly factor nubp2 homolog
5744	heat shock 70 kda protein 4l-like
5746	hormone-sensitive lipase
5749	hypothetical protein KGM_01384
5754 [b, c]	cg11638 cg11638-pa
5755	zinc finger protein 271 (zinc finger protein 7) (zinc finger protein znfphex133) (epstein-barr virus-induced zinc finger protein) (znf-eb) (ct-zfp48) (zinc finger protein
5756	zinc finger C2HC domain-containing protein 1C-like isoform X2
5757	cg9383-pa
5758	transmembrane gtpase marf-like
5760	shc sh2 domain-binding protein 1 homolog b-like
5763	phosphatidylglycerophosphatase and protein-tyrosine phosphatase 1-like
5765	protein transport protein sec23
5766	golgi snap receptor complex member 2
5767	single-strand selective monofunctional uracil dna glycosylase-like
5768	trafficking protein particle complex subunit 8-like
5769	coiled-coil-helix-coiled-coil-helix domain-containing protein mitochondrial-like
5770 [b]	hypothetical protein KGM_13863, *stops*
5771	hypothetical protein KGM_21008
5776	decaprenyl-diphosphate synthase subunit 2-like

5778	dnaJ-like protein 9
5783	hypothetical protein
5785	ubiquitin protein ligase
5788	scp-related protein
5789	fanconi anemia group d2
579	iroquois-class homeodomain protein irx
5790	dna topoisomerase 1-like
5791	alpha-tocopherol transfer
5793	venom serine carboxypeptidase-like
5794	down syndrome cell adhesion molecule-like protein dscam2-like
5795	hypothetical protein KGM_05133
5797	dym_drome ame: full=dymeclin
5803	ribonuclease h1
5804	hypothetical protein KGM_14348
5805	nedd8 ultimate buster 1-like
5806	heterogeneous nuclear ribonucleoprotein l-like
5807	glutaredoxin 2
5808	amyloid beta a4 precursor protein-binding family b member 2-like
5809	dappled
5813 [a]	adenylyl cyclase-associated protein 1-like isoform x3
5816	glutathione peroxidase
5817	hypothetical protein KGM_08102
5820	importin alpha
5822	mannosyl-oligosaccharide glucosidase gcs1-like
5823	hypothetical protein KGM_04190
5824	fas-associated factor 1

5825	defective proboscis extension response
5826	zinc finger protein 77
5827	dna replication factor cdt1
5832	adp-ribosylation factor-like protein 1-like
5834	rho guanine nucleotide exchange factor 11
5835	luciferin 4-monooxygenase
5836 [b]	tachykinin precursor
5837	fidgetin-like protein 1-like
5839	regulator of chromosome condensation
584	cleavage and polyadenylation specificity factor subunit 4-like
5840	neurexin-4-like isoform x2
5841	probable maleylacetoacetate isomerase 2-like
5843	ap-3 complex subunit mu-1
5844	dipeptidyl peptidase 9
5846	transcriptional regulator atrx homolog
5847	telomerase-binding protein est1a-like
585	protein n-terminal glutamine amidohydrolase-like
5850	low quality protein: probable e3 ubiquitin-protein ligase mycbp2-like
5852	nucleolar protein 10-like
5854	glutathione s-transferase epsilon 2
5855 [a]	low quality protein: wd repeat and fyve domain-containing protein 3-like
5856	protein timeless homolog
586	hexokinase
5864	nad-dependent protein deacylase sirtuin- mitochondrial isoform x1
5865	protein odr-4 homolog

5873	acid phosphatase-like protein 2-like
5874	blastoderm-specific protein 25d
5876	acyl- delta desaturase-like
5879	insulin-related peptide binding protein
5881	mitochondrial nadh:ubiquinone oxidoreductase subunit
5882	ubiquitin carboxyl-terminal hydrolase 20-like
5885	glucosyl glucuronosyl transferases
5887	t-complex protein 1 subunit alpha-like
5888	transmembrane and coiled-coil domain-containing protein 7
5890	multidrug resistance-associated protein 7-like
5891	zinc finger cchc domain-containing protein 8 homolog
5892	flocculation protein flo11-like
5893	phosphoglucose isomerase
5894	No hits found
5896	fh2 domain-containing protein 1
5897	chromosome segregation protein smc
5902	rho gtpase-activating protein 18-like
5903	fibrillin 2
5905	cg13055 cg13055-pa
5912	proto-oncogene tyrosine-protein kinase ros-like
5915	ddb1- and cul4-associated factor 12-like
5916	cytochrome p450
5917 [a]	adp-ribosylation factor 1
5918	pre-mrna-splicing factor cwc25 homolog
5920	kinase d-interacting substrate of 220 kda-like
5922	ribosome biogenesis protein brx1 homolog

5923	leucine-rich repeat protein soc-2 homolog
5925	peroxisome biogenesis factor 1
5928	serine threonine-protein kinase sik3-like
5929	succinate-semialdehyde mitochondrial-like
593	trna-dihydrouridine synthase
5930	elav-like protein 3
5934	poly(adp-ribose) glycohydrolase
5935 [a]	neurogenic protein mastermind-like
5936	pyridine nucleotide-disulfide oxidoreductase domain-containing protein 1-like
5940	ferritin hch
5945	ribosomal protein l13a
5946	phosphatidylinositol 4-kinase type-ii
5947	nitric-oxide synthase like protein
5949	ribosomal protein l10a
5952	protein fam73b-like
5955	spermatogenesis-associated protein 20-like
5956	sv2-like protein 1
5958	rap1 gtpase-activating protein 1
5959	at-rich interactive domain-containing protein 4b-like
5961	facilitated trehalose transporter tret1-like
5962	e3 ubiquitin-protein ligase march5
5963	g-protein coupled receptor mth2-like
5964	crossover junction endonuclease mus81-like
5967	beta-lactamase-like protein 2 homolog isoform x1
5969	putative roundabout

5971	dna (cytosine-5)-methyltransferase -like
5972	reticulon nogo
5973	proton-coupled amino acid transporter 1
5975	methoprene-tolerant protein
5976	nucleolar complex protein 3 homolog
5977	iron-sulfur cluster assembly enzyme mitochondrial-like
5978	cationic amino acid transporter 3-like
5979	low quality protein: e3 ubiquitin-protein ligase hectd1-like
598	protein transport protein sec24c-like
5980	neuropeptide precursor protein precursor
5982	proto-oncogene tyrosine-protein kinase ros-like
5983	s-phase kinase-associated protein 2-like
5985	neurotactin-like isoform x1
5986	irregular chiasm c-roughest
5989	palmitoyltransferase zdhhc6-like
5990	anaphase-promoting complex subunit 5-like
5991	wd repeat-containing protein cg11141-like
5993	bardet-biedl syndrome 1 isoform x1
5994	ubiquitin thioesterase otubain-like
5995	dna repair protein rad21
5999	dual specificity testis-specific protein kinase 2-like
60	sequestosome-1-like isoform x3
6000 [b]	rhodopsin, partial
6001	sugar transporter
6002	3-phosphoinositide-dependent protein kinase 1
6006	ribosomal protein s19

6007	ribosomal protein l22
6008	endoplasmic reticulum oxidoreduction 1-like protein
601	mlx-interacting protein
6010	ribosomal protein lp1
6011	zinc finger protein 729-like
6012	putative activin receptor type I
6013	eukaryotic initiation factor 4a
6014	filamin
6015	transmembrane protein 132B-like
6017 [a]	protein phosphatase slingshot homolog 2-like isoform x2
6021	leucine-rich repeat flightless-interacting protein 2-like
6023	protein star-like
6025	beaten path ic
6027	iduronate 2-sulfatase
6029	troponin t
603	dentin sialophospho
6030	ubiquitin-conjugating enzyme e2-230k
6033	protein sidekick
6034	protein efr3 homolog cmp44e-like
6035	autophagy-related protein 2-like protein b
6036	actin binding protein
6038	g-protein coupled receptor
6040	vacuolar protein sorting-associated protein 26-like
6043	o-glucosyltransferase rumi homolog
6045	catenin delta-2-like
6048	atp synthase-beta

605	phd finger protein
6054	protein fam98a-like
6058	histone-lysine n-methyltransferase setd1b-like
6059	ras-related c3 botulinum toxin substrate 1 (rho small gtp binding protein rac1)
6060	ankyrin repeat and mynd domain-containing protein 2-like
6061	zinc finger protein
6062	hypothetical protein KGM_15031
6064	phosphatidylinositol 3-kinase 60
6065	protein bowel-like
6066	alpha-2-macroglobulin-like protein 1-like
6067	lysosomal alpha-glucosidase-like
6068	cytochrome p450 cyp314a1
607	e3 ubiquitin-protein ligase smurf2
6070	v-type proton atpase subunit e-like
6072	elongation factor 1 alpha
6073	sugar phosphate exchanger 2
6079	atp-dependent dna helicase q4-like
6080	ribosomal protein l26
6082	enhancer of mrna-decapping protein 3-like
6083	arf6 guanine nucleotide exchange factor
6085	probable cation-transporting atpase 13a3-like
6086	breakpoint cluster region protein
6087	g-protein coupled receptor mth2-like
6088	equilibrative nucleoside transporter 3-like
609	solute carrier organic anion transporter family member 5a1-like

6091	swi snf-related matrix-associated actin-dependent regulator of chromatin subfamily d member 1-like
6092	cell division cycle 25
6095	f-box lrr-repeat protein 15-like
6096	run domain-containing protein 1-like
6099	carboxy-terminal domain rna polymerase ii polypeptide a small phosphatase 1-like
61 [a]	scarlet
610	probable phosphorylase b kinase regulatory subunit beta-like
6100	e3 ubiquitin-protein ligase herc2-like
6105	en protein binding engrailed nuclear homeoprotein-regulated protein
6107	probable tyrosine--trna mitochondrial-like
6109	n-alpha-acetyltransferase 40-like
6110	ubiquitin conjugation factor e4 a-like
6111	sex peptide receptor
6112	histone deacetylase 3-like
6113	wd repeat-containing protein 3-like
6114 [b]	acetylcholine receptor subunit alpha-like 2-like
6115	collagen alpha-1 chain-like
6120	protein fam8a1-like
6123	tubulin beta-1 chain
6125	double-stranded rna-specific editase adar-like
6128	zinc finger swim domain-containing protein 5
613	proteasome activator complex subunit 3-like
6131	sodium-dependent noradrenaline transporter-like
6132	geranylgeranyl transferase type-2 subunit beta-like

6133	monocarboxylate transporter
6134	apolipoprotein d-like
6136	saxophone
6137	e3 ubiquitin-protein ligase hectd1
6138	gtp-binding protein rheb homolog
614	amp-activated protein gamma regulatory subunit
6141	retinol dehydrogenase 11-like
6142	e3 ubiquitin-protein ligase rnf25-like
6143	udp-glucose 4-epimerase
6145	hypothetical protein KGM_06296
6147	hypothetical protein KGM_13295
6150 [b]	lysocardiolipin acyltransferase 1-like
6151	lysosomal-trafficking regulator-like
6153	retinol-binding protein
6154 [a, b, c]	arrestin homolog isoform 1
6155	sterol regulatory element-binding protein 1
6156	probable atp-dependent rna helicase ddx47-like
6157	pecanex-like protein 1-like
6158	monocarboxylate transporter
6160	fetal alzheimer falz
6161	zinc finger ccch-type with g patch domain-containing
6163	hypothetical protein KGM_00692
6168	zinc finger protein
617	and pleckstrin domain protein 1-like protein
6170 [b]	down syndrome cell adhesion molecule-like protein dscam2-like
6173	histone acetyltransferase type b catalytic subunit-like

6174	cytoplasmic fmr1-interacting
6175	hypothetical protein KGM_07541
6176	cytochrome p450
6177	growth hormone-inducible transmembrane
6178	mesenchymal stem cell protein dscd75
618	cg9893
6180	forkhead box protein k1
6183	ubiquitin carboxyl-terminal hydrolase 31-like
6184	alkaline phosphatase
6185	ultraspiracle protein
6187	protein suppressor of white apricot-like
6189	reverse transcriptase
6190	iron-sulfur cluster assembly 1-like mitochondrial
6194	angio-associated migratory cell
6198	succinyl-coa synthetase beta chain
620	tetraspanin 47f
6201	high-affinity copper uptake protein 1
6203	zinc finger protein 709-like
6206 [a]	homeobox protein homothorax-like
6207	double-strand-break repair protein rad21 homolog
6209	peroxisomal n1-acetyl-spermine spermidine oxidase
621	protein gawky-like
6212	replication protein a3
6213	couch isoform n
6214	pre-mrna-processing factor 6-like
6216 [a, b]	blue-sensitive visual pigment (BRh)

6217	unknown protein, partial
622	exocyst complex component 2-like
6220	ribosomal protein s6
6221	methyltransferase nsun6-like
6222	gdnf family receptor alpha-3
6223	ribosomal protein l30
6227	ribosomal protein l10
6228	peroxisomal membrane protein pex16
623	ubiquitin-conjugating enzyme e2 o
6230	muscle m-line assembly protein unc-89-like
6233 [a]	homeodomain-interacting protein kinase 2-like
6235	glyoxylate reductase hydroxypyruvate reductase
6236	serine threonine-protein kinase mtor-like
624	actin-binding protein anillin-like
6245	translation initiation factor eif-2b subunit delta
6247	zinc finger protein
6248	adenylate cyclase
6250	wd repeat-containing protein 59
6251	f-box lrr-repeat protein 16-like
6252	nascent polypeptide associated complex protein alpha subunit
6254	pyrroline-5-carboxylate dehydrogenase
6255	transmembrane and coiled-coil domains protein 1-like
6256	proteasome activator complex subunit 4
6257	torn and diminished isoform c
6258	gram domain-containing protein 1b-like
626	host cell factor 1-like

6266	tudor domain-containing protein 1-like
627	xylosyltransferase oxt-like
6270	counting factor associated protein d-like
6274	hypothetical protein KGM_07163
6278 [b]	corticotropin-releasing factor-binding
6279	alkylated dna repair protein alkb homolog 8-like
6281	c-jun nh2-terminal kinase
6284	molybdenum cofactor sulfurase
6288	probable atp-dependent rna helicase cg8611-like
6289	axoneme-associated protein
6291	heat shock cognate 70 protein
6293	probable enoyl- mitochondrial-like
6295	organic cation transporter 1-like
6296	cytochrome c
6299 [b]	hypothetical protein KGM_18874
63	calcyclin-binding protein
6303	meiosis arrest female protein 1 homolog
6304	hypothetical protein KGM_03744
6309	atrial natriuretic peptide-converting enzyme-like
631	sumo-activating enzyme subunit 2-like
6310	hypothetical protein KGM_00431
6312	inositol-tetrakisphosphate 1-kinase-like
6315	protein fam188a homolog
6316	capicua protein
6317	dead-box helicase dbp80
6318	bone morphogenetic protein receptor type-2-like

6319	ras gtp exchange son of sevenless
632	endonuclease exonuclease phosphatase family domain-containing protein 1
6320	kat8 regulatory nsl complex subunit 1-like
6323	math and lrr domain-containing protein pfe0570w-like isoform x1
6324	vesicle associated protein
6326	fgfr1 oncogene partner 2 homolog
6328	sorting nexin-7
633	hect e3 ubiquitin ligase
6330	gmp synthase
6332	hypothetical protein KGM_18990
6333	abc transporter
6334	es2 protein
6335	hemolymph proteinase 16
6336	hypothetical protein KGM_15294
6339	low quality protein: lysine-specific demethylase 6a-like
6340	nucleolar protein 56-like
6341	serine threonine-protein phosphatase 2a 65 kda regulatory subunit a alpha isoform-like
6342	progestin and adipoq receptor family member 3-like
6344	atp-dependent rna helicase dhx30
6347	small subunit processome component 20 homolog
6351	cg3683
6352	bcl2 adenovirus e1b 19 kda protein-interacting protein 3-like
6354	zinc finger protein 502- partial
6357	variable lymphocyte receptor a
6358	calcyphosphine isoform 1

6359	nadh-ubiquinone oxidoreductase ashi subunit
636 c	vacuolar h
6361	nadh dehydrogenase
6363	u2 small nuclear ribonucleoprotein auxiliary factor 2
6364	protein kr-h2-like
6365	retinol dehydrogenase 14-like
6368	putative coracle
6369	chaperone activity of bc1 complex- mitochondrial-like
6370	ribosomal protein l23
6371	ribosomal protein s30
6372	protein spire-like isoform x4
6374	adamts-like protein 3-like
6377	james isoform a
6378	bifunctional protein ncoat
6381	two pore potassium channel protein sup-9-like
6386	inward rectifier potassium channel 2-like
6390	u3 small nucleolar rna-associated protein 15 homolog
6391	protein jagunal-like
6392	activator of 90 kda heat shock protein atpase homolog 1-like
6395	coagulation factor xi
6396	vacuolar atp synthase subunit s1
6397	cytoplasmic dynein 2 heavy chain 1-like
6398	eukaryotic translation initiation factor 3 subunit 5
64	signal-induced proliferation-associated 1-like protein 2-like
640	wd-repeat protein

6400	elongation of very long chain fatty acids protein aael008004-like isoform x1
6401	kelch-like ech-associated protein 1-like
6402	17-beta-hydroxysteroid dehydrogenase 14-like
6404	cytosolic purine 5 -nucleotidase-like
6407	organic solute transporter alpha-like
6408	serine protease inhibitor 5
6409	No hits found
6410	dorsal-ventral patterning tolloid-like protein 1
6411	myelin transcription factor 1-like protein
6413	3-hydroxyisobutyryl-coenzyme a hydrolase
6416	ubiquitin-conjugating enzyme e2-24 kda-like
6417	aladin-like
6421	zinc finger protein
6422	gtp-binding protein parf
6423	ribosomal protein l11
6424	ribosomal protein l31
6425	imaginal disc growth factor 4
6426	udp-glucose glycoprotein:glucosyltransferase
6427	probable bax inhibitor 1-like
6429	hypothetical protein KGM_10924
6431	mau2 chromatid cohesion factor homolog
6434	pdz domain protein
6435	protein zwilch-like
6436	protein ric1 homolog
6438	hypothetical protein KGM_14452

644	low-density lipoprotein receptor-related protein 2
6440	zinc finger protein 569
6442	trna-dihydrouridine synthase 1-like
6443	encore protein
6444 [a]	paired box protein pax-6-like isoform x2
6447	similar to cg6426
6453 [a]	ribosomal protein l8
6454	glyoxalase domain-containing protein 4-like
6455	zinc finger protein 271-like isoform x1
6457	thymidine kinase mitochondrial
6460	methionine-trna synthetase
6462	phosphatidate phosphatase lpin2-like
6463	aminopeptidase n-like
6464	rho gtpase-activating protein 11a
6465	heat shock protein
6466	ectodysplasin
6467	dynamin-like 120 kda mitochondrial-like isoform x1
6468	kinesin-like protein costa-like
6469	hypothetical protein KGM_00243
6470	rrna methyltransferase 3
6471	wd-repeat protein
6475	heat repeat-containing protein 7a
6479	cystathionine beta-synthase-like
6481	cuticular protein cpg24
6482	unknown secreted protein
6485	exosome complex component rrp43-like

6486	class d atypical g-protein coupled receptor
6487	putative 1,4-alpha-glucan branching enzyme
6489 [c]	beta-arrestin 1
649	nad-dependent deacetylase sirtuin-2-like
6492	tyrosine transporter
6496	ribosomal protein s15
6498 [c]	calmodulin
6499	alpha- -mannosyl-glycoprotein 2-beta-n-acetylglucosaminyltransferase
65	probable atp-dependent rna helicase ddx55 homolog
650	nucleoside diphosphate-linked moiety x motif mitochondrial-like
6500 [a]	segment polarity protein dishevelled-like protein dvl-3
6501	reticulon nogo receptor
6507	adp atp translocase
6508	n-myc downstream regulated
6509	peroxisome assembly factor 2-like isoform x2
6511	hypothetical protein KGM_08170
6513	protein lap4-like
6514	f-box lrr-repeat protein 4
6516	ga-binding protein subunit beta-2- partial
6517	poly -specific ribonuclease parn-like
6518	malic enzyme
652	glucosyl glucuronosyl transferases
6520	serine protease
6521	e3 ubiquitin-protein ligase trim37-like isoform x3
6522	low quality protein: upf0518 protein agap011705-like

6523	probable phenylalanine--trna ligase alpha subunit-like
6525	methyltransferase-like protein 2-a-like
6526	cytochrome p450
6527	nuclear pore complex protein nup98-nup96-like
6530	cyclin g
6531	ribosomal rna methyltransferase nop2-like
6532	dnaj homolog subfamily c member 22-like
6533	cysteine serine-rich nuclear protein 2
6534	ribosomal protein l24
6536	cuticular protein 4
6537	chromobox protein homolog 1
6539	vlc
654	cklf-like marvel transmembrane domain-containing protein 4-like
6540	heparan sulfate 2-o-sulfotransferase pipe-like
6541	cg12811 cg12811-pa
6542	beclin 1-associated autophagy-related key regulator-like
6543	translation initiation factor eif-2b subunit alpha-like
6545	ras-related protein rab-43-like
6547	hypothetical protein KGM_15107
6548	peroxiredoxin-4-like isoform x1
6549	piggybac transposable element-derived protein 3-like
655	zinc finger protein
6550	microphthalmia-associated transcription factor-like
6552	myeloid leukemia factor
6554	ras-related and estrogen-regulated growth inhibitor-like
6555	chaperonin

6558	eukaryotic peptide chain release factor gtp-binding subunit
656	uncharacterized protein LOC101737164
6562	alkaline tissue-nonspecific isozyme-like
6564	potassium-dependent sodium-calcium exchanger
6567	pyruvate dehydrogenase
6568	target of rapamycin complex subunit lst8-like
6571	ribosomal protein s26
6573	probable atp-dependent rna helicase ddx17-like
6574	nonmuscle myosin essential light chain
6578	hypothetical protein KGM_19707
658	down syndrome cell adhesion molecule-like protein cg42256-like
6580	translationally controlled tumor protein
6582	glyceraldehyde-3-phosphate dehydrogenase
6584	zinc finger protein
6585	pyrazinamidase nicotinamidase
6586	hypothetical protein KGM_03567
6587	abc transporter
6588	adp-ribosylation factor-binding protein gga1-like
6592	macrophage erythroblast attacher-like isoform 1
660	putative survivin
6602	upf0668 protein c10orf76 homolog
6604	dullard-like protein
6608	hypothetical protein KGM_16338
6610 [b]	allatostatin neuropeptide precursor (*AstA*)
6615	succinate dehydrogenase subunit mitochondrial
6617	glutathione s-transferase

6618	a disintegrin and metalloproteinase with thrombospondin motifs 7-like
6620	solute carrier family 41 member 1-like
6622	zinc finger protein 135-like
6624	ubiquitin-conjugating enzyme e2 j1-like
6633	wd repeat-containing protein 37-like
6635	lysyl-trna synthetase
6636	cdk5 regulatory subunit-associated protein 3-like
6637	hypothetical protein KGM_06966
664	inactive rhomboid protein 1- partial
6645	succinyl-CoA:3-ketoacid coenzyme A transferase 1, mitochondrial-like
6646	upf0505 protein c16orf62 homolog
6649	solute carrier family 35 member f5-like
665	protein naked cuticle homolog 2-like
6650	mixed-lineage leukemia mll
6651	ribosomal rna processing protein 1 homolog
6652	antennal binding protein
6653	cytochrome c oxidase subunit iv
6654 [b]	sarcoplasmic calcium-binding protein 2
6655	mitochondrial cytochrome c oxidase subunit vb
6656	ribosomal protein s16
6657	control protein hctl025
6659	beat protein
6661 [b]	kda small heat shock protein
6664	methionine-r-sulfoxide reductase b1-like isoform 1
6665	inhibition of apoptosis protein 2

6667 [b]	lysosome-associated membrane glycoprotein 1-like
6668	protein ovo
6669	gtp:amp phosphotransferase mitochondrial
667	vacuolar protein sorting-associated protein 13c-like
6671	ctp synthase
6673	scaffold attachment factor b2-like
6677	short-chain dehydrogenease reductase
6680	dihydrouridine synthase domain containing protein
6682	tfiih basal transcription factor complex helicase xpd subunit-like
6685	van gogh protein
6686	h/aca ribonucleoprotein complex non-core subunit naf1-like
6687	putative voltage- gated calcium channel alpha subunit
6690	dna-directed rna polymerase iii subunit rpc3-like
6691	tyrosine-protein kinase wsck-like
6692	hypothetical protein KGM_15928
6693	hypothetical protein KGM_15276
670	hypothetical protein KGM_17101
6703	hypothetical protein KGM_14148
6705	transformation transcription domain-associated protein
6707	viral a-type inclusion protein
6708	peptidoglycan-recognition protein 2-like
6709	palmitoyl-protein thioesterase 1-like isoform x1
671	metal response element-binding transcription factor-1
6710	tenascin- partial
6714	puromycin-sensitive aminopeptidase-like
6715	zinc metalloproteinase yil108w-like

6716	sluggish isoform a
6718	hypothetical protein KGM_14094
672	a disintegrin and metalloproteinase with thrombospondin motifs 7-like
6722	fork head domain transcription factor partial
6724	diphthine synthase
6726	cysteine protease atg4b-like
6727	gamma-interferon-inducible lysosomal thiol reductase
6728	heat shock protein
6729	alpha-n-acetylglucosaminidase
673	protein lingerer
6730	nadph cytochrome p450 reductase
6736	ras-related protein rab-1a-like
6740	poly(adp-ribose) glycohydrolase
6742	beta-tubulin cofactor d
6743	transmembrane protein 198-like isoform x2
6751	myoneurin-like isoform x2
6752	hypothetical protein KGM_18714
6753	serine protease inhibitor partial
6754	microsomal glutathione s-transferase-like
6755	progestin and adipoq receptor family member 4-like
6757	alpha-galactosidase alpha-n-acetylgalactosaminidase
6758	regulator of chromosome condensation-like
6759	uncharacterized protein LOC101737461
6760	eukaryotic translation initiation factor 3 subunit d
6762	cyclin l

6765	oligopeptide transporter
6766	hypothetical protein KGM_22420
6767	histone h4 transcription factor-like
6771	39s ribosomal protein mitochondrial
6772	pyruvate kinase
6774	chaoptin-like
6775	upstream stimulatory factor 1-like
6777	ccr4-not transcription complex subunit 3
6778	hypothetical protein KGM_19457
678	protein mis12 homolog isoform x1
6780	zinc finger protein 177
6781	sp1070 cg9138-pa
6782 [a]	protein peanut-like
6783	tubulin-specific chaperone d
6784	n-acetylglucosaminyl-phosphatidylinositol biosynthetic
6785	synaptosomal-associated protein 25-like isoform x3
6786	glycogen-binding subunit 76a-like isoform x2
6787	aminopeptidase n
6789	protein piccolo-like
6790	vacuolar atp synthase subunit d
6792	mitochondrial 28s ribosomal protein s14
6794	glyoxylate reductase hydroxypyruvate reductase-like
6797	glycogen
6798	dna repair protein rev1-like
6799	nitrate reductase
68	eukaryotic translation initiation factor 3 subunit h

680 [a]	forkhead box transcription factor subgroup n2
6800	serine threonine-protein kinase vrk
6801	cytochrome c oxidase assembly protein cox16 mitochondrial-like
6803	protein phosphatase methylesterase 1-like
6806	translocon-associated delta subunit
6809	eukaryotic translation initiation factor 3 subunit k
6810	rab gdp-dissociation inhibitor
6812	proteasome subunit alpha type-2-like
6813	fructose -bisphosphate aldolase
6815	cg2765 cg2765-pa
6819	apolipoprotein d
682	mitochondrial ribosomal protein s11
6820	uncharacterized threonine-rich gpi-anchored glyco isoform x2
6821	af4 fmr2 family member 4-like
6822	ribosomal protein s12
6823	hypothetical protein KGM_01838
6824 [b]	glucose dehydrogenase
6825	ribosomal protein s2
6827	c-maf-inducing isoform 1
683 [a, b, c]	long wavelength-sensitive visual pigment (LWRh)
6832	inhibitor of growth protein 3
6834	protein preli-like
6839	fibulin 1 and
6840	transmembrane emp24 domain-containing protein 7
6841	nephrin-like
6843	thioredoxin domain-containing protein 11-like

6844	mediator of rna polymerase ii transcription subunit 13-like
6846	choriogenin h
6847	regulator of g-protein signaling loco-like
6848	uridine 5 -monophosphate synthase
6849	leucine-rich repeat-containing protein 57-like
6852	calponin homology domain-containing protein ddb_g0272472-like isoform x19
6854	bromodomain and phd finger-containing
6858	5 -3 exoribonuclease 2 homolog
686	zinc transporter 5
6861	solute carrier organic anion transporter family member 3a1-like
6862	protein smg7
6865	cell death activator cide-b
6867	eukaryotic translation initiation factor 6
6869	tmc6 protein
6873	dendritic cell protein
6875	hypothetical protein KGM_06323
6877	gamma-soluble nsf attachment protein
6879	actin-related protein 2 3 complex subunit 3-like
688	eukaryotic translation initiation factor 2 subunit 1-like
6880	probable cation-transporting atpase 13a3-like
6881	agrin-like isoform x5
6882	ankyrin repeat domain-containing protein 17-like
6884	ring finger protein 168
6885	uncharacterized protein LOC101744916 isoform X1
6886	sodium-coupled monocarboxylate transporter 1-like

6888	mitochondrial atp synthase coupling factor 6
6889 [c]	innexin 2
689	polycomb protein su(z)12
6892	cysteinyl-trna synthetase
6893	major facilitator superfamily domain-containing protein 6-like
6894	replication factor c subunit 2
6895	oligosaccharyl transferase
6896	ribosomal protein l27a
6897 [b]	lachesin
6898	exocyst complex component 6b-like isoform 1
6899	ribosomal protein s5
690	growth and transformation-dependent protein
6900	adp-ribosylation factor-like protein 3
6903	apolipoprotein d-like protein
6904	trans-golgi network integral membrane protein 2-like
6906	low quality protein: transcription initiation factor tfiid subunit 4-like
6907	reverse transcriptase
6908	dna helicase ino80-like
6909	mediator of rna polymerase ii transcription subunit 14
691	neuroblastoma-amplified sequence-like
6910	n-terminal acetyltransferase a complex catalytic subunit ard1
6911	odorant binding protein
6912	aliphatic nitrilase
6916	probable s-acyltransferase at2g14255-like
6918	dna-3-methyladenine glycosylase 1

6919	serine 3-dehydrogenase
692	cue domain-containing protein 1-like
6921	huntingtin-interacting protein 1
6923	protein eva-1-like
6925	cytoplasmic protein nck1-like
6929	conserved oligomeric golgi complex subunit 7-like
693	brain protein i3-like
6930	tyrosine-protein kinase btk29a
6933	protein memo1-like
6936	versican core protein
6937	zinc finger protein
6938	expanded
6939	neuroblastoma-amplified sequence
694	mitochondrial inner membrane protease subunit 2-like
6942	phospholipid-transporting atpase 1 (aminophospholipid flippase 1)
6944	membrane associated ring finger
6945 [c]	vacuolar h
6947	homeobox protein goosecoid-like
6949	death-related protein
695	fatty acyl- reductase cg5065-like
6951	juvenile hormone binding protein
6953	ubiquitin-like modifier-activating enzyme 5
6954	low quality protein: cadherin-23-like
6957	h aca ribonucleoprotein complex subunit 4
6958	mitochondrial gtpase 1-like
696	atp-dependent rna helicase me31b-like

6962	cathepsin l
6964	ribosomal protein l35
6965	myotubularin-related protein 13
6967	ribosomal protein s7
6968	ubx domain-containing protein 1
697	protein smg7-like isoform x1
6970	uncharacterized protein LOC101892431
6972	ubiquitin-conjugating enzyme e2 variant 2-like
6973	protein-associating with the carboxyl-terminal domain of ezrin-like
6975	ribosomal protein s23
698	rna-binding protein 45-like
6980	mitochondrial assembly of ribosomal large subunit protein 1-like
6981	cd151 antigen-like isoform x2
6982	fused lobes
6983	hypothetical protein KGM_19656
6985	ubiquitin carboxyl-terminal hydrolase 36-like
6987 [a]	low quality protein: dachshund homolog 1-like
6988	ras-related and estrogen-regulated growth inhibitor-like
6989	transcriptional enhancer factor tef-1
699	f-spondin
6993	low-density lipoprotein receptor-related protein 2
6994	lachesin-like isoform x3
6998	ubiquitin carboxyl-terminal hydrolase 10-A-like
7	glutamyl-trna amidotransferase subunit a
70	peripheral plasma membrane protein cask

700	hypothetical protein KGM_14168
7004	neural wiskott-aldrich syndrome
7005	hypothetical protein KGM_17495
701	actin cytoskeleton-regulatory complex protein pan1-like
7011	trafficking kinesin-binding protein milt-like
7015	chaperonin subunit 6a zeta
7016	serine-rich adhesin for platelets-like isoform x1
702	splicing factor u2af 38 kda subunit
7021	transcriptional adaptor 3
7025	mucin-22-like isoform x9
7026	glutamate synthase
7028	gtp-binding protein
7034	exocyst complex component 6b-like isoform 1
7035	delta(3,5)-Delta(2,4)-dienoyl-CoA isomerase, mitochondrial-like
7036	far upstream element-binding protein 1-like
7037	tau-like protein
7042	regulatory-associated protein of mtor
7043	ras-related and estrogen-regulated growth inhibitor-like
7044	oxysterol-binding protein 1
7047	protein gpr107-like
7048	methylenetetrahydrofolate dehydrogenase
7049	downstream of receptor kinase
705	probable atp-dependent rna helicase pitchoune-like
7053	transcriptional repressor p66-beta-like
7054	inactive dipeptidyl peptidase 10-like
7057	fruitless

7058	organic cation transporter
7059	putative coronin
7060	rho gtpase-activating protein 5
7063	zinc finger protein 484-like
7064	bm8 interacting protein 2d-2
7067	g-protein coupled receptor 143-like
7070	hypothetical protein KGM_14526
7072	cg11981-pa
7074	leucine-rich repeats and immunoglobulin-like domains protein 3-like
7076	potassium channel modulatory factor 1
7078	vacuolar atp synthase subunit f
708	transmembrane protein 161b-like
7081	purine biosynthesis protein pur6
7084	hypothetical protein KGM_13363
7085	estradiol 17 beta-dehydrogenase
7086	remodeling and spacing factor 1
7087	kelch-like protein diablo-like
7088	atlastin-like isoform x3
709	tetratricopeptide repeat protein 28-like
7092	membrane-associated guanylate kinase
7094	atrial natriuretic peptide-converting enzyme-like
7095	protein efr3 homolog cmp44e-like
7099 [c]	phosphatidate phosphatase-like
71	twinkle mitochondrial-like
710	transmembrane protein 104 homolog

7102	netrin-1-like
7103	chromatin target of prmt1
7104	seminal fluid protein CSSFP048
7105	hypothetical protein KGM_19707
7106	cyclin-dependent kinase 14-like
7108	isocitrate dehydrogenase
7109	zinc finger hit domain-containing protein 2-like
7113	disheveled associated activator of morphogenesis
7114	zinc transporter zip3-like
7116	alpha-tocopherol transfer
7117	fructose-1,6-bisphosphatase
7118	star-related lipid transfer protein mitochondrial-like
7120	flap endonuclease gen-like
7121	myotubularin-related protein 10-b-like
7129	ig-like and fibronectin type-iii domain-containing
7133	phosphatidylinositol-4-phosphate 5-kinase type-1 alpha
7134	exostosin-1-like
7135	syntaxin-like protein
7136 [a]	ap-3 complex subunit delta-like
7137 [b]	venom acid phosphatase acph-1-like
7139	sodium-dependent phosphate transporter
714	pentatricopeptide repeat-containing protein mitochondrial-like
7140	hypothetical protein KGM_14478
7142	heat shock protein
7143	mitochondrial sodium hydrogen exchanger 9b2-like
7144	integrator complex subunit 1-like isoform x2

7145	sugar transporter
7146	zinc finger ccch domain-containing protein 10-like
7147	sodium potassium-dependent atpase beta-2 subunit
7149	small nuclear ribonucleoprotein sm d1
715	adp-ribosylation factor-like protein 5b-like
7154	hypothetical protein KGM_03595
7158	serine-enriched protein
7159	mpn domain-containing protein cg4751
716	cklf-like marvel transmembrane domain-containing protein 4-like
7160	leucine-rich repeat protein shoc-2-like
7161	low quality protein: an1-type zinc finger protein 4
7162	annexin b11 isoform a
7163	transport and golgi organization protein 1
7164	glucosamine--fructose-6-phosphate aminotransferase
7165	red protein
7166	40s ribosomal protein s3a
7167	fggy carbohydrate kinase domain-containing
7168	pyruvate mitochondrial
7171	ras-related protein rab-40c-like
7173	sex-determining protein fem-1
7174	max-binding protein mnt-like
7175	calcium-transporting atpase type 2c member 1-like
7176	tyrosine-protein kinase hopscotch
7179	t-box transcription factor tbx3
7181	transcription factor sox-2-like
7182	longitudinals partial

7185	Beta-1,3-glucan-binding protein
7187	potential grip domain golgi protein
7190	methyl-CpG-binding domain protein 4-like isoform X1
7192	fyve finger-containing phosphoinositide
7194	ankyrin repeat domain-containing protein 17
72	cytoplasmic dynein heavy chain 1b
720	angiotensin-converting enzyme-like isoform x2
7200	serine threonine-protein phosphatase 2a 56 kda regulatory subunit alpha isoform-like
7205	macrophage mhc class i receptor 2-like protein
7208	origin recognition complex subunit 4
721	mitogen activated protein kinase kinase kinase mekk5
7210	thyroid hormone receptor interactor 12
7211	ribosomal protein s4
7212	cullin 3
7215	atp-dependent clp protease proteolytic mitochondrial-like
7216	protein kinase c-binding protein nell1
7217	proteasome alpha 4 subunit
7218	replication factor c subunit 4-like
7219	hepatitis b virus x associated hbxa
722	hsc70-interacting isoform x1
7223	epsin-like protein
7228	alpha-mannosidase 2-like
7234	probable methyltransferase tarbp1-like
7235	glutaminase kidney mitochondrial-like
7239	zinc finger protein 26

724	beat protein
7241	probable atp-dependent rna helicase kurz-like
7242	histone h3 methyltransferase
7243	wd repeat-containing protein 61-like
7244	transcriptional repressor scratch 2-like
7245	hypothetical protein KGM_06662
7246	p160 coactivator fisc
7247	zinc finger and btb domain-containing protein 34-like isoform 2
7248	methyltransferase-like protein 9-like
7249	phosphatidylinositol-5-phosphate 4-kinase type-2 alpha-like
7250	adp-dependent glucokinase-like
7251	protein yippee-like 2
7254	hypothetical protein KGM_11994
7256	serine 3-dehydrogenase
7258	ribonucleic acid binding protein s1
7259	dna replication licensing factor mcm5-like
7260	uncharacterized protein LOC101742533
7262	circadian clock-controlled protein
7263	heparan-alpha-glucosaminide n-acetyltransferase-like
7264	beat protein
7265	valyl-trna synthetase
7267 [a]	innexin inx1-like
7270	f-box/lrr-repeat protein 14-like
7271	af162221_163 orf163
7273	2-aminoethanethiol dioxygenase-like
7275	protein rolling stone-like

7276	thioredoxin-like protein
7277	aael009271- partial
7279	renin receptor-like isoform 1
728	ankyrin repeat domain-containing protein 17
7280	cg7484
7281	hypothetical protein KGM_12763
7282	nuclear cap-binding protein subunit 2-like
7283	cyclin-g-associated kinase-like
7288	vacuolar atp synthase subunit g
7289	hypothetical protein KGM_08637
7290	stromal cell-derived factor 2 precursor
7292	major facilitator superfamily domain-containing protein 12-like
7294	coiled-coil domain-containing protein 142
7295	pleiotropic regulator 1
7296	enhancer of mrna-decapping protein 4-like
7297	protein canopy homolog 1-like
7298	protein 5nuc-like isoform x1
7299	prolyl endopeptidase-like
73	protein zer-1-like protein
7305	inorganic phosphate cotransporter
7309	aminopeptidase n
731	conserved oligomeric golgi complex subunit 3-like
7311	forkhead box protein n3-like
7315 [c]	dopa decarboxylase
7317	lysosomal alpha-glucosidase-like
7322	mesoderm development candidate 2

7323	udp-glucose 6-dehydrogenase-like
7324	facilitated trehalose transporter tret1-like
7325	chromodomain-helicase-dna-binding protein 1
7326	von willebrand factor a domain-containing protein 9-like
7328	isoleucyl trna synthetase
7330	protein farnesyltransferase geranylgeranyltransferase type-1 subunit alpha-like
7331	member ras oncogene family
7333	phosphoenolpyruvate synthase-like
7335	without children, isoform C
7336	rho gtpase-activating protein 6
7339	hypothetical protein KGM_19306
7340	optineurin-like isoform x1
7342	uncharacterized protein LOC101744228
7344	cleavage stimulation factor subunit 1-like
7345	integrator complex subunit 10-like
7346	c-type lectin 27kd
7348	p23-like protein
7349	protein crumbs-like
735	exportin-4-like isoform 2
7350	probable transcription-associated protein 1-like
7351	rna-directed dna polymerase from mobile element jockey- partial
7352	mothers against decapentaplegic homolog 3-like
7355	inter-alpha-trypsin inhibitor heavy chain H4-like
7357	alkyldihydroxyacetonephosphate synthase-like
7358	cactus

7362	protein fam40a-like
7363	actin cytoskeleton-regulatory complex protein pan1-like
7364	glucose 6 phosphate dehydrogenase
7365	fibronectin type-iii domain-containing protein 3a
7366	inosine-5 -monophosphate dehydrogenase
7367	h+ transporting atp synthase o subunit isoform 1
7369	dna ligase 3
7370	pre-mrna-processing factor 39-like
7372	ubiquitin carboxyl-terminal hydrolase 34-like
7374	myb-like protein x-like isoform x1
7375	rho guanine nucleotide exchange factor 10-like
7377	ribonucleoside-diphosphate reductase large subunit-like
7379	single ig il-1-related receptor-like
7381	short coiled-coil protein b-like
7386	map microtubule affinity-regulating kinase 3-like
739	deoxyhypusine hydroxylase-like
7391	hypothetical protein KGM_20512
7392	multidrug resistance-associated protein 7-like
7393	serine threonine-protein kinase mtor
7395	probable serine threonine-protein kinase ddb_g0283337-like isoform x1
7397	hypothetical protein KGM_22041
7398	ecdysone-induced protein isoform b
7399	protein trs85-like protein
7400 [b]	inositol polyphosphate 5-phosphatase k-like
7405	cg4553 cg4553-pa

7407	hypothetical protein KGM_08073
7410	zinc transporter
7411	suppressor of t-cell receptor signaling 1
7412	prolactin regulatory element-binding
7413	wolframin-like isoform x2
7414 [b]	otopetrin
7416	mms19 nucleotide excision repair protein homolog
7417	mitochondrial manganese superoxide dismutase
7418	tubulin-folding cofactor b-like isoform x1
7423	set1 ash2 histone methyltransferase complex subunit ash2-like
7424	bestrophin-2-like isoform 1
7426	retinol dehydrogenase 11-like
7427	transmembrane protein 129-like
743	chitin binding peritrophin-
7436	presqualene diphosphate phosphatase
7444	g1 s-specific cyclin-d3 isoform x1
7447	epidermal growth factor receptor kinase substrate 8-like
7448	prip interacting pimt
745	aldehyde dehydrogenase
7450	arginine kinase
7451	transmembrane protein 55b-like
7452	probable atp-dependent rna helicase ddx52-like
7453	substrate- ligase
7456	magnesium transporter protein 1-like
7457	viral a-type inclusion protein
7459	diphosphomevalonate decarboxylase

746	conserved oligomeric golgi complex subunit 4-like
7460	hypothetical protein KGM_15143
7462	coronin-2b-like isoform 1
7463	tetratricopeptide repeat protein 5-like
7468	pancreatic triacylglycerol lipase
7469	juvenile hormone epoxide hydrolase
747	rho gtpase-activating protein 20
7471	hypothetical protein KGM_11226
7474	hypothetical protein KGM_08650
7475	zinc finger protein 84
7476	proteasome 54kd subunit
7478	unconventional myosin-xv-like
7479	structural maintenance of chromosomes protein 2-like
748	transmembrane protein 38a
7480	hypothetical protein KGM_17447
7481	dna replication licensing factor mcm4
7484	protease m50 membrane-bound transcription factor site 2 protease
7485	mediator of dna damage checkpoint protein 1
7487	paternally expressed 3
7488	ecdysteroid 22-kinase
749	mediator of rna polymerase ii transcription subunit 18-like
7491 [c]	phosphatidylinositol synthase
7492	nedd8-activating enzyme e1 regulatory subunit-like
7493	low quality protein: dachshund homolog 1-like
7496	deoxyribose-phosphate aldolase
7498	coiled-coil domain-containing protein 50-like

7506	carnitine o-acetyltransferase
7509	tubulin polyglutamylase ttll4-like
7510	perq amino acid-rich with gyf domain-containing protein 2
7511	prip interacting pimt
7514 [b]	basic helix-loop-helix protein
7516	luciferin 4-monooxygenase-like
7517 [b]	defective proboscis extension
7520	t-complex protein 11-like protein 1-like
7521	btb poz domain-containing adapter for cul3-mediated degradation protein 3-like
7523	ceramide synthase 5-like
7525	transcription cofactor vestigial-like protein 4-like isoform X2
7526	cyclin j
7528	dnaj homolog subfamily c member 13-like
7529	ccr4-not transcription
753	uncharacterized protein LOC101735560
7530	cuticular protein isoform c
7532	cg8281-like partial
7533	transcription factor e2f2
7535	hiv tat-specific factor 1 homolog
7537	pab-dependent poly -specific ribonuclease subunit 2-like
7539	baculoviral iap repeat-containing protein 6-like
7540	hypothetical protein KGM_13628
7543	cathepsin l
7544	guanine nucleotide-binding protein subunit alpha homolog
7547	n-acetyl-d-glucosamine kinase-like

7549	metal-response element-binding transcription factor 2
7552	ecdysteroid-regulated 16 kda protein
7553	host specificity j domain partial
7556	gmp synthase
7558	activating transcription factor 2 isoform 1
7559	nadh dehydrogenase
7562	transcription elongation factor spt6-like
7563 [a]	protein crumbs-like
7564	hypothetical protein KGM_07492
7566	tpa: cuticle protein
7568	dynein light chain tctex-type 1-like
7569	disks large-associated protein 5-like isoform x4
757	wd repeat-containing protein 74-like
7571	SINA2
7572	thymosin isoform 2
7573	dolichyl-diphosphooligosaccharide--protein glycosyltransferase subunit stt3a-like
7577	Bumetanide-sensitive sodium-(potassium)-chloride cotransporter
7579	hypothetical protein KGM_15618
7581	guanylate cyclase soluble subunit beta-1-like
7584	transmembrane 4 isoform a
7586	28 kda heat- and acid-stable phosphoprotein-like
7588	somatostatin receptor type 2-like
759	uncharacterized protein LOC101742119 isoform X2
7591	protein kinase c and casein kinase substrate in neurons
7592	yth domain family protein 1-like

7593	serine threonine protein kinase
7594	upf0568 protein c14orf166 homolog
7595	dna polymerase epsilon catalytic subunit a-like
7598	upf0480 protein c15orf24 homolog
7599	regulator of g-protein signaling loco-like
76	mitochondrial ribosomal protein l39
7601	hypothetical protein KGM_01708
7602	nck-associated protein 5
7609	galactosylgalactosylxylosylprotein 3-beta-glucuronosyltransferase i-like
7612	glycine n-acyltransferase-like protein 3-like
7613	neuronal growth regulator 1- partial
7615	hypothetical protein KGM_00190
7617	homeobox protein cut
7623	3-hydroxyisobutyrate dehydrogenase
7624	cell division protein kinase 5
7625	wd repeat-containing protein 61-like
7626	adenosine deaminase-like
7635	bromodomain adjacent to zinc finger domain protein 1a
7638	k3 protein
764	28s ribosomal protein mitochondrial-like
7641	malic enzyme
7647	down syndrome cell adhesion molecule-like protein 1
7648	cell division control protein 45 homolog
7649	gs1- isoform b
765	galactosylgalactosylxylosylprotein 3-beta-glucuronosyltransferase I-like

7652	nk homeobox 7
7653	takeout jhbp like protein
7654	low quality protein: kinesin-like protein kif13a-like
7655	flavin-dependent monooxygenase fmo3b
7657	calsyntenin-1-like
766	aryl hydrocarbon receptor nuclear translocator homolog isoform x1
7661 [a]	cryptochrome 1
7663	synaptotagmin-like protein 4-like
7667	sodium-independent sulfate anion transporter-like
7673	apoptotic protease-activating factor 1-like
7676	tata-binding protein-associated factor 172
7677	pre-mrna 3 -end-processing factor fip1-like
768	protein spinster homolog 1-like
7681	hemicentin-1-like isoform x3
7684 [a]	steroid receptor seven- isoforms b c-like
7685	rna pseudouridylate synthase domain-containing protein 2-like
7686	rho-related gtp-binding protein
7688 [a]	protein decapentaplegic
769	e3 ubiquitin-protein ligase topors-like
7691	glycogenin
7697	hmct_bommo ame: full=hemocytin ame: full=humoral lectin flags: precursor
77	tho complex subunit 2
7705	eater
7706	b-cell cll lymphoma 11a
7707	atp-dependent rna helicase abstrakt

771	long-chain-fatty-acid-- ligase 1-like
7710	histone-lysine n-methyltransferase ehmt1-like
7712	small heat shock protein
7714	matrix metalloproteinase-14-like isoform x1
7718	usp6 n-terminal-like
7719	dna repair and recombination protein rad54-like
7722	protein phosphatase 1 regulatory subunit 16a-like
7725	mitochondrial import receptor subunit tom70-like
7726	beaten path iia
7728	protein c10-like
7729	atp-binding cassette sub-family b member mitochondrial-like
773	x-linked retinitis pigmentosa gtpase regulator-like protein
7731	atp-binding cassette sub-family a member 1-like
7734	wd repeat-containing protein 18-like
7736	protein fam185a-like
774	sugar transporter
7741	dna helicase mcm9
7743	zinc finger protein 84
7746	decaprenyl-diphosphate synthase subunit 1-like
7747	cell death regulator
7748	low quality protein: cartilage oligomeric matrix
775	otu domain-containing protein 7b-like
7760	actin-related protein 8-like
7762	probable gdp-fucose transporter-like
7763	cysteine sulfinic acid decarboxylase
7766	chemosensory protein 1 partial

7767	solute carrier family 35 member f1
7768	ribosomal protein l44e
7769	ribosomal protein l36
7770	hypothetical protein KGM_13512
7771	ribosomal protein s17
7773	mitogen-activated protein kinase kinase kinase 4-like
7775	dystroglycan-like isoform x4
7778	necap-like protein cg9132-like
778	tgf-beta-activated kinase 1 and map3k7-binding protein 1-like
7784	hypothetical protein KGM_02809
7788	zinc finger protein 155-like
7789	cg3595
7798	btb poz domain-containing protein 3-like
78	negative elongation factor e-like
780	gpi-anchored wall transfer protein 1
7802	ras-related protein rab-21
7803	transmembrane protein ddb_g0273707 ddb_g0273361-like
7804	beta-galactosidase-like
7805	connectin-like
7806 [a]	mediator of rna polymerase ii transcription subunit 12-like
7807 [a]	map7 domain-containing protein 2 isoform x9 (*dan*)
7808	vacuolar protein sorting-associated protein
781	autism susceptibility gene 2
7811	hypothetical protein KGM_15082
7812 [b]	sodium potassium-transporting atpase subunit beta-2
7816	runt

7817	v-type proton atpase catalytic subunit a
7820	myelin expression factor 2-like
7822 [a]	ataxin-2-like protein
7824	rho guanyl-nucleotide exchange factor
7827	serine threonine-protein phosphatase 2a catalytic subunit alpha isoform-like
7829	n-acetyl-glucosamine-6-phosphate isomerase
783	birt-hogg-dube homolog
7831	zinc finger protein
7834	putative Xsmad4a
7837	serine protease
7838	chemosensory protein-14
7839	cg6847 cg6847-pa
784	eukaryotic translation initiation factor 4 gamma 2-like
7840	glutamate decarboxylase
7841	importin subunit beta
7842	activating transcription factor of chaperone-like
7843	ribosomal protein s14
7844	ribosomal protein l13
7845	ribosomal protein l9
7847	protein arginine n-methyltransferase
7848	e3 ubiquitin-protein ligase rbbp6-like
7849	Mod(mdg4)-heS00531
785	transcription factor tfiiib component b partial
7850	phospholipase d2
7851	heat shock protein

7852	tbc1 domain family member 14- partial
7854	glucose transporter
7855	roundabout, isoform B
7857	mki67 fha domain-interacting nucleolar phospho
7859	stam-binding a-like
7860	sodium solute symporter
7862	low quality protein: protein fam91a1
7864	lamin dm0-like
7865	broad-complex core protein isoform 6
7867	gephyrin-like isoform x1
787	hypothetical protein KGM_15656
7871	uncharacterized protein LOC101737397
7872	g-protein coupled receptor moody-like
7873	hypothetical protein KGM_12445
7874	hypothetical protein KGM_09034
7875	organic cation transporter
7877 [b]	carbonic anhydrase-related protein 10-like
788	2-hydroxyacyl- lyase 1
7882	ribosomal protein s6 kinase
7885	ran-binding protein 9-like
7886	ribosomal protein s8
7887	ribosomal protein s18
7888	dystrobrevin beta-like
7889	autophagy related protein atg8
7890	lanb2
7891	cyclophilin a

7892	ribosomal protein s25
7893	ribosomal protein l18a
7896	cg12324 protein
7898	sphingomyelin phosphodiesterase
7899	60s acidic ribosomal protein p2
79	translation elongation factor 2
790	ubiquitin carboxyl-terminal hydrolase 43
7900	gamma-aminobutyric acid receptor isoform 3b
7902	forkhead box protein p1-like
7903	baculoviral iap repeat-containing protein 6
7906	histone-lysine n-methyltransferase pff1440w-like isoform x1
7907	probable g-protein coupled receptor 158
7909	mediator of rna polymerase ii transcription subunit 17-like
7910	mucin 68d
7913	major facilitator superfamily domain-containing protein 8-like
7914	endothelial-monocyte activating polypeptide ii
7917	actin binding protein
7918	ankyrin repeat and lem domain-containing protein 2-like
7923	general transcription factor 3c polypeptide 1-like
7925	protein-cysteine n-palmitoyltransferase porcupine-like
7926	wd repeat-containing protein 75-like
7927	hypothetical protein KGM_08739
7928	core alpha -fucosyltransferase
793	hypothetical protein KGM_07922, *crim*
7930	atp-dependent rna helicase ddx1-like
7932	hypothetical protein KGM_04823

7933	pyroglutamyl-peptidase 1
7934	integrator complex subunit 5-like
7935	deoxyribonuclease tatd
7940	small subunit processome component 20 homolog
7941	threonine--trna cytoplasmic-like isoform x2
7942 [a]	rap guanine nucleotide exchange factor 6-like isoform x10
7943	glutamyl aminopeptidase-like
7944	inactive ubiquitin carboxyl-terminal hydrolase 53-like isoform x2
7946	hypothetical protein KGM_05651
7951	adenylate kinase 2
7952	dentin sialophosphoprotein
7953	dynactin subunit 6
7954	hypothetical protein KGM_00373
7957	cell division protease ftsh
7960	leucine-rich transmembrane protein
7961	protein mon2 homolog
7963	atrial natriuretic peptide receptor 1-like
7965	ribosomal protein l3
7966	serine protease easter-like
7967	ribosomal protein l7
7968	abhydrolase domain-containing protein 2-like
797	at-rich interactive domain-containing protein 4a
7970	ribosomal protein l14
7971	e3 ubiquitin-protein ligase rfwd2
7972	chiffon, isoform A
7974	ribosomal protein s3

7976	dag protein
7978	40s ribosomal protein s13
7982	upf0501 protein kiaa1430-like protein
7983	modifier of mdg4
7989	6-phosphofructo-2-kinase fructose- -bisphosphatase short form
7990	hypothetical protein KGM_09817
7992	vesicle transport protein use1
7993	m7 -mrna hydrolase-like
7994	synaptotagmin 4
80	mitochondrial ribosomal protein s25
8001	sterol regulatory element-binding protein 1
8006	nudix hydrolase 8-like
8007	disintegrin and metalloproteinase domain-containing protein 10-like
8009	hypothetical protein KGM_12054
801	maestro heat-like repeat-containing protein family member 1-like isoform x2
8010	phosphatidate phosphatase lpin2-like
8016	epidermal growth factor receptor kinase substrate 8-like
8017 [b]	transcription factor collier-like
802	probable ribonuclease zc3h12c-like
8020	alpha-tocopherol transfer
8021	cd98hc amino acid transporter protein
8022	hypothetical protein KGM_02212
8024	probable phospholipid-transporting atpase iib-like
8026	cellular retinaldehyde binding protein
8027	casein kinase i isoform alpha

8029	phosphatidylinositol-4-phosphate 3-kinase c2 domain-containing beta polypeptide
8030	ras-related protein rab-14-like
8031	nicotinamide mononucleotide adenylyltransferase 1
8032	1-acylglycerophosphocholine o-acyltransferase 1-like
8033	inositol -trisphosphate receptor-like
8035	deleted in autism protein 1 homolog
8036	probable nucleoporin nup54-like
8038	ribosomal protein l15
8039	probable 26s proteasome non-atpase regulatory subunit 3-like
8040	translation initiation factor 2 gamma subunit
8042	low quality protein: e3 ubiquitin-protein ligase cbl-like
8045	ribosomal protein l32
8047	nuclear pore complex protein nup205
8048	venus kinase receptor
8049	phosphoinositide 3-kinase adapter protein 1
8051 [b]	aminoacylase-1-like
8053	hypothetical protein KGM_13066
8056	protein alpha isoform-like isoform x2
8058	myosin light chain 2
8059	protein hira homolog
8066	sine oculis-binding protein
8067	protease inhibitor 4
8069	charged multivesicular body protein 2b
8072	thyroid hormone receptor interactor 12
8074	hypothetical protein KGM_20796

8075	syntaxin 5a
8076	vacuolar protein sorting-associated protein 35-like
8077 [b]	peritrophin type-a domain protein 3
8080	mushroom body large-type kenyon cell-specific protein 1-like
8084	hypothetical protein KGM_19303
8085	hectd1 protein
8086	alcohol dehydrogenase
8087	gremlin-2
8088	was protein family homolog 1-like
8089	N-acetyllactosaminide beta-1,3-N-acetylglucosaminyltransferase-like isoform X1
809	hypothetical protein KGM_11587
8092	formin cappuccino
8093	hectd1 partial
8094	probable nucleoporin nup54-like
8095	rho-related gtp-binding protein
8096	homeobox protein invected
8098	heat shock 70 kda protein 4l-like
81	neurobeachin-like
8100	probable nadh dehydrogenase
8102	hypothetical protein AaeL_AAEL007493
8103	hypothetical protein KGM_15690
8105	protein tkr-like
8106	transmembrane protein 131-like
8108	protein takeout-like
8109	fibroblast growth factor receptor

8111	coiled-coil domain-containing protein 47
8112	hypothetical protein LOC100160548
8114	glutaminyl-trna synthetase
8117	fch and double sh3 domains protein 2-like
8119	zinc finger c4h2 domain-containing
8120	organic cation transporter
8121	gamma-aminobutyric acid type b receptor subunit 1-like
8122	actin-binding protein ipp
8125	tbc domain-containing protein kinase-like protein
8127	calcium-transporting atpase type 2c member 1-like
813	nicotinate phosphoribosyltransferase-like isoform x2
8130	pollen-specific leucine-rich repeat extensin-like protein 3-like
8131	nuclear migration protein nudc
8134	hypothetical protein KGM_15878
815	uncharacterized protein LOC101740198
818	telomerase-binding protein est1a
82	very long-chain specific acyl- mitochondrial-like
821	ecdysteroid-regulated 16 kda protein
822	lysM and putative peptidoglycan-binding domain-containing protein 3-like isoform X2
824	dedicator of cytokinesis protein 3
827	peroxisomal membrane protein 11c-like
828	glycerol kinase
830	pyridoxal-dependent decarboxylase domain-containing protein 1-like
831	alkaline nuclease
832	neural precursor cell developmentally down-regulated 1

834	double-strand-break repair protein rad21 homolog
836	myotubularin-related protein 13
837	nck-associated protein 5
838	spectrin beta
839	camp-dependent protein kinase r2
840	prolyl 3-hydroxylase 2-like
846	integrator complex subunit 1-like
848	bis(5 -nucleosyl)-tetraphosphatase
849	steroid dehydrogenase
852	a disintegrin and metalloproteinase with thrombospondin motifs like
853	syntaxin-18-like
854	lrp16 protein
856 [c]	transient receptor potential-gamma
857	sumo ligase
860	bilin binding protein 1
862	n-acetylgalactosaminyltransferase 7-like
863 [b]	gustatory receptor 4
864	rna polymerase ii-associated factor 1 homolog
869	low quality protein: reticulocalbin-2-like
871	ankyrin repeat and lem domain-containing protein 1-like
873	acidic mammalian chitinase-like
874	ER membrane protein complex subunit 1-like
875	rho gtpase-activating protein 18-like isoform x1
880 [b]	titin-like
885 [b]	sodium-dependent phosphate transporter

886	nodal modulator 3-like
888	amino acid transporter
890	upf0505 protein c16orf62 homolog
894 [b]	nephrin-like
895	multidrug resistance protein 1a-like isoform x1
896	polycystic kidney disease protein 1-like 3-like isoform 2
898	low quality protein: gtpase-activating
899	fibroin-modulator-binding protein-1
9	ld14 cg12664-pb
90 [a]	vacuolar protein sorting-associated protein 41 homolog
901	facilitated trehalose transporter tret1-like
902	mediator of rna polymerase ii transcription subunit 14-like
904	myosuppressin receptor
907	creb-binding protein
911	glutamate--cysteine ligase regulatory subunit-like
912	transcription elongation factor spt6-like
913	probable fatty acid-binding
915	protein transport protein sec31a
919	probable glutamine--trna ligase-like
922	ubiquitin carboxyl-terminal hydrolase 2
924	No hits found
925	integrin alpha-ps2-like
926	myosin vii
928	thyroid receptor-interacting protein 11
93	crag protein
930	domain-containing histone demethylation protein

933	blood meal-induced protein
935	udp-glycosyltransferase ugt41a1 precursor
936	hypothetical protein KGM_06697
938	protein tweety-like
94	growth arrest and dna damage-inducible protein gadd45 alpha-like
940	dedicator of cytokinesis protein 7-like
941	protein fam91a1-like isoform x1
942	ecdysteroid regulated protein
944	transcription factor sp3-like
945	glycerol kinase
947	breakpoint cluster region protein
95	pancreatic triacylglycerol lipase-like
951	dnaj homolog subfamily c member 10
952	dedicator of cytokinesis protein 7-like
953	sorting and assembly machinery component 50 homolog
954	protein twisted gastrulation-like
96	zinc finger protein 395-like
960	adenosine isoform b
962	carbonic anhydrase 1
964	ribosome biogenesis protein bms1 homolog
967	No hits found
968	beat protein
969	X box binding protein-1
97	caspase-1
970	male-specific lethal 3
971	hypothetical protein KGM_20020

972	adenine phosphoribosyltransferase
973	octopamine receptor
974	uncharacterized protein LOC101736981
977	tyrosine-protein phosphatase corkscrew-like
978	inhibitor of growth ing4
979	ubiquitin-protein ligase e3c-like
980	neuronal calcium sensor 2-like
983	nfat isoform f
986	adamts-like protein 3-like
987	putative N-acetyllactosaminide beta-1,3-N-acetylglucosaminyltransferase
990	titin-like isoform x2
991	inhibitor of growth protein 3-like
994	twelve cysteine protein 1
995	dynamin-associated protein

[a] indicates vision-related genes.

[b] indicates genes commonly upregulated in heads.

[c] indicates phototransduction genes.

Table S5. Annotation of unique orthologs

unique orthologs in the *sara-hortense-erato* clade	
ID in *H. erato* transcriptome assembly	Annotation
comp1043_c0_seq1|1.m.166	f-spondin
comp10973_c1_seq1|1.m.691	NA
comp13142_c0_seq1|1.m.847	NA
comp13776_c0_seq1|1.m.1114	Down syndrome cell adhesion molecule-like protein Dscam2-like
comp13789_c0_seq1|1.m.1119	phosphopantothenate--cysteine ligase-like
comp14430_c0_seq1|1.m.1694	tudor and KH domain-containing protein-like
comp14462_c0_seq1|1.m.1718	sphingosine-1-phosphate lyase-like
comp14669_c0_seq1|1.m.1885	Agrin precursor, putative
comp15344_c0_seq1|1.m.2112	hypothetical protein KGM_12941
comp15925_c1_seq1|1.m.2282	protein msta, isoform B-like
comp15939_c0_seq1|1.m.2286	serine/threonine-protein kinase fused-like
comp16808_c0_seq1|1.m.2473	maestro heat-like repeat-containing protein family member 1-like isoform X1
comp17178_c0_seq1|1.m.2559	transcriptional regulator ATRX
comp17881_c0_seq1|1.m.2799	NA
comp18943_c1_seq1|1.m.3305	kinesin-like protein KIF16B
comp19381_c0_seq1|1.m.3526	6-phosphofructo-2-kinase, isoform A
comp19390_c0_seq1|1.m.3533	cation transport ATPase
comp19748_c0_seq1|1.m.3743	K3 protein
comp20087_c0_seq1|1.m.3957	cryptochrome 1
comp20170_c1_seq1|1.m.4009	nucleolar pre-ribosomal-associated protein 1-like
comp20615_c0_seq1|1.m.4260	Cad88C CG3389-PA
comp20903_c0_seq1|1.m.4398	dynein beta chain, ciliary-like
comp20957_c1_seq1|1.m.4412	loricrin-like isoform X1
comp21287_c0_seq1|1.m.4557	trypsin, alkaline C-like
comp21301_c0_seq1|1.m.4561	BEL12_AG transposon polyprotein
comp21471_c0_seq1|1.m.4651	enhancer of mRNA-decapping protein 4-like
comp21527_c0_seq1|1.m.4678	neurexin 1, isoform A
comp21579_c0_seq1|1.m.4708	reverse transcriptase
comp21980_c0_seq1|1.m.4888	bifunctional heparan sulfate N-deacetylase/N-sulfotransferase-like
comp22026_c0_seq1|1.m.4911	Down syndrome cell adhesion molecule-like protein Dscam2-like
comp22595_c0_seq1|1.m.5169	single-minded homolog 1-like
comp23405_c0_seq1|1.m.5652	NAD(P) transhydrogenase, putative
comp23937_c0_seq1|1.m.6157	cuticle protein 3
comp23961_c0_seq1|1.m.6182	heat shock protein 90
comp24034_c0_seq1|1.m.6268	neuronal acetylcholine receptor subunit beta-3
comp24258_c0_seq1|1.m.6569	hypothetical protein KGM_08194

comp24918_c0_seq1|1.m.7139	leucine-rich transmembrane protein
comp25186_c0_seq1|1.m.7379	red protein
comp25447_c2_seq1|1.m.7620	m-rdgb2 retinal degeneration protein b
comp25490_c0_seq1|1.m.7658	probable phospholipid-transporting ATPase IIB-like
comp25566_c0_seq1|1.m.7722	Uncharacterized 50 kDa protein in type I retrotransposable element R1DM
comp25840_c0_seq1|1.m.7962	serine/threonine-protein kinase PRP4 homolog isoform X1
comp25919_c0_seq1|1.m.8077	putative mitochondrial inner membrane protein-like
comp26016_c0_seq1|1.m.8173	coiled-coil and C2 domain-containing protein 1-like
comp26170_c0_seq1|1.m.8356	phosphoenolpyruvate carboxykinase
comp27297_c0_seq1|1.m.9902	60 kDa heat shock protein, mitochondrial-like
comp27314_c0_seq1|1.m.9924	phospholipid scramblase 1, putative
comp27608_c0_seq1|1.m.10429	Putative glycerol kinase 3
comp27610_c0_seq1|1.m.10435	long-chain fatty acid transport protein 4-like
comp27636_c0_seq2|1.m.10483	dorsal interacting protein 3
comp27713_c0_seq1|1.m.10599	hypothetical protein KGM_11742
comp27851_c0_seq1|1.m.10829	nucleoporin NUP188 homolog
comp28125_c1_seq1|1.m.11308	microtubule-actin cross-linking factor 1-like isoform X7
comp28137_c0_seq1|1.m.11393	C-myc promoter-binding protein-like
comp28312_c1_seq1|1.m.11733	PREDICTED: uncharacterized protein LOC101736405
comp28498_c0_seq1|1.m.12047	neuroendocrine convertase 2-like
comp28531_c0_seq1|1.m.12101	protein unc-13 homolog C-like
comp28656_c0_seq1|1.m.12403	gag-like protein
comp28714_c2_seq1|1.m.12501	hypothetical protein KGM_06299
comp28723_c0_seq1|1.m.12515	cadherin-23-like isoform 1
comp28818_c0_seq2|1.m.12717	52 kDa repressor of the inhibitor of the protein kinase-like
comp29007_c0_seq1|1.m.13108	Down syndrome cell adhesion molecule-like protein Dscam2-like
comp29025_c0_seq1|1.m.13126	ubiquitin conjugation factor E4 A-like
comp29093_c0_seq1|1.m.13322	nitric oxide synthase
comp29327_c0_seq1|1.m.13877	endonuclease-reverse transcriptase
comp29390_c0_seq1|1.m.14028	glycerol-3-phosphate acyltransferase 1, mitochondrial-like
comp29424_c0_seq1|1.m.14082	Apolipophorins
comp29517_c0_seq1|1.m.14252	scavenger receptor class B member 3
comp29644_c0_seq1|1.m.14515	DNA topoisomerase 2-binding protein 1-like
comp29826_c0_seq1|1.m.15043	Seryl-tRNA synthetase, mitochondrial
comp29990_c0_seq1|1.m.15502	timeless
comp30225_c0_seq1|1.m.16207	exosome complex exonuclease RRP44-like
comp30243_c0_seq4|1.m.16275	mpv17-like protein-like
comp30252_c0_seq1|1.m.16308	glypican-4-like isoform X2

comp30345_c0_seq2|1.m.16648	enzymatic polyprotein; Endonuclease; Reverse transcriptase, putative
comp30907_c1_seq1|1.m.18737	CLK4-associating serine/arginine rich protein-like
comp31056_c0_seq1|1.m.19741	protein lin-37 homolog
comp31111_c0_seq1|1.m.20105	ATP-citrate synthase
comp31271_c0_seq1|1.m.20729	unconventional myosin-Va-like
comp31494_c0_seq1|1.m.21626	aldehyde oxidase 2
comp31578_c5_seq1|1.m.22051	facilitated trehalose transporter Tret1-like
comp31590_c0_seq1|1.m.22074	putative rab6 gtpase activating protein, gapcena
comp31674_c1_seq7|1.m.22584	gustatory receptor 23
comp31805_c0_seq1|1.m.23123	E3 ubiquitin-protein ligase hyd-like
comp31872_c0_seq1|1.m.23391	hypothetical protein KGM_17397
comp31945_c0_seq1|1.m.23842	glass protein
comp32106_c0_seq1|1.m.25595	reverse transcriptase
comp32189_c2_seq1|1.m.26356	MEK1 interacting protein 1
comp32212_c0_seq1|1.m.26472	SH3 and multiple ankyrin repeat domains protein 3
comp32282_c0_seq1|1.m.26892	protein abrupt-like
comp32467_c0_seq1|1.m.28547	nucleoporin NUP188 homolog
comp32576_c0_seq1|1.m.29347	cadherin 88C
comp32703_c0_seq1|1.m.30655	zinc finger protein 594-like
comp32746_c0_seq1|1.m.30915	FAD synthase-like
comp32793_c0_seq1|1.m.31266	piggyBac transposable element-derived protein 3-like
comp33030_c0_seq1|1.m.34345	ATP dependant DNA helicase
comp33050_c3_seq1|1.m.34529	Ubiquitin carboxyl-terminal hydrolase 5
comp33074_c0_seq1|1.m.35292	cGMP-dependent PKG
comp33088_c1_seq1|1.m.35425	bifunctional heparan sulfate N-deacetylase/N-sulfotransferase-like
comp33341_c0_seq1|1.m.41148	tetratricopeptide repeat protein 17-like
comp33446_c0_seq1|1.m.43305	knockout, isoform A
comp33467_c0_seq1|1.m.43824	coiled-coil domain-containing protein 147
comp33492_c0_seq1|1.m.44574	Pol protein
comp33513_c1_seq1|1.m.45090	PREDICTED: uncharacterized protein LOC101746874
comp33549_c0_seq1|1.m.45750	envelope fusion protein
comp33559_c0_seq1|1.m.45866	endonuclease and reverse transcriptase-like protein
comp34238_c0_seq1|1.m.46494	aldehyde dehydrogenase
comp34256_c0_seq1|1.m.46507	1-phosphatidylinositol-3-phosphate 5-kinase-like
comp34353_c0_seq1|1.m.46560	signal recognition particle receptor subunit beta-like
comp34683_c0_seq1|1.m.46700	ATP-binding cassette sub-family A member 13
comp34739_c0_seq1|1.m.46723	myosin-IB-like isoform X2
comp35272_c0_seq1|1.m.46898	CG42340, isoform B
comp35383_c0_seq1|1.m.46940	cation-transporting ATPase
comp36580_c0_seq1|1.m.47214	monocarboxylate transporter
comp36891_c0_seq1|1.m.47281	semaphorin-2A-like isoform X2

comp37436_c0_seq1|1.m.47377	probable ATP-dependent RNA helicase YTHDC2-like
comp37937_c0_seq1|1.m.47474	BEL12_AG transposon polyprotein
comp38538_c0_seq1|1.m.47562	hypothetical protein KGM_08728
comp41037_c0_seq1|1.m.47914	intraflagellar transport protein 122 homolog
comp41966_c0_seq1|1.m.48040	chemosensory protein 1 variant
comp43302_c0_seq1|1.m.48176	zinc finger protein 99-like isoform X1
comp46899_c0_seq1|1.m.48498	CG41538 CG41538-PA
comp47848_c0_seq1|1.m.48584	NA
comp49526_c0_seq1|1.m.48724	ceramide synthase 6-like isoform X2
comp51234_c0_seq1|1.m.48822	NA
comp57161_c0_seq1|1.m.49084	peroxidase-like
comp594_c1_seq1|1.m.69	CCR4-NOT transcription complex subunit 1
comp61424_c0_seq1|1.m.49244	scavenger receptor class B member 1-like
comp62860_c0_seq1|1.m.49283	5-HT2
comp648_c0_seq1|1.m.82	non-LTR retrotransposon CATS
comp6766_c0_seq1|1.m.480	NA

unique orthologs in the *cydno-melpomene-hecale* clade

ID in *H. melpomene* transcriptome assembly	Annotation
comp10518_c0_seq1|7.m.3153	retrovirus-related Pol polyprotein from transposon TNT 1-94
comp10697_c0_seq1|7.m.3216	NA
comp10697_c0_seq1|7.m.3215	predicted protein
comp1143_c0_seq1|7.m.111	lipase
comp12440_c0_seq1|7.m.3573	fibrillin-2-like
comp12958_c1_seq1|7.m.3773	NA
comp13016_c0_seq1|7.m.3816	sorbitol dehydrogenase
comp1329_c0_seq1|7.m.145	nose resistant to fluoxetine protein 6-like
comp13597_c1_seq1|7.m.4001	regulator of telomere elongation helicase 1 homolog
comp13738_c1_seq1|7.m.4040	reverse transcriptase
comp13872_c0_seq1|7.m.4080	atrial natriuretic peptide-converting enzyme-like
comp13956_c0_seq1|7.m.4102	cytochrome c oxidase assembly protein 3, mitochondrial-like
comp14093_c0_seq1|7.m.4198	dentin sialophosphoprotein-like
comp14555_c1_seq1|7.m.4507	CG17360, isoform B
comp15079_c1_seq1|7.m.4937	glutamate receptor ionotropic, kainate 2-like
comp15402_c0_seq1|7.m.5115	supporter of activation of yellow protein-like
comp15652_c0_seq1|7.m.5277	alpha-tocopherol transfer protein-like
comp16056_c1_seq1|7.m.5529	endonuclease-reverse transcriptase
comp16118_c0_seq1|7.m.5559	NA
comp1644_c0_seq1|7.m.185	NA
comp16536_c0_seq1|7.m.5860	endonuclease-reverse transcriptase
comp16543_c0_seq1|7.m.5869	predicted protein

comp16737_c0_seq1|7.m.6074	CG6236, isoform A
comp16796_c0_seq1|7.m.6152	endonuclease-reverse transcriptase
comp16954_c3_seq3|7.m.6315	zinc finger protein
comp17133_c0_seq1|7.m.6504	non-LTR retrotransposon CATS
comp17203_c0_seq1|7.m.6575	RNA-directed DNA polymerase from mobile element jockey-like
comp17203_c1_seq1|7.m.6576	predicted protein
comp17372_c0_seq1|7.m.6769	putative epidermal cell surface receptor-like
comp17446_c0_seq1|7.m.6845	endonuclease-reverse transcriptase
comp17532_c0_seq1|7.m.6939	reverse transcriptase
comp1807_c0_seq1|7.m.199	tigger transposable element-derived protein 6-like
comp18295_c0_seq1|7.m.7923	origin recognition complex subunit 4
comp18656_c0_seq1|7.m.8450	endothelin-converting enzyme 1-like
comp1888_c0_seq1|7.m.210	PREDICTED: uncharacterized protein LOC101746992
comp19082_c0_seq1|7.m.9235	Large proline-rich protein BAT2
comp19131_c0_seq1|7.m.9337	metalloproteinase, putative
comp19242_c0_seq1|7.m.9624	proline-rich protein 13
comp19743_c0_seq1|7.m.10463	gag-like protein
comp19815_c1_seq1|7.m.10590	Zinc finger protein 271
comp20131_c0_seq1|7.m.11359	serine protease
comp20298_c0_seq1|7.m.11650	PREDICTED: uncharacterized protein LOC101746620, partial
comp20450_c0_seq1|7.m.11996	retinol dehydrogenase 14-like
comp20456_c0_seq2|7.m.12024	transposase
comp20745_c0_seq1|7.m.12830	NA
comp21094_c1_seq1|7.m.14050	neuropilin and tolloid-like protein 2-like
comp21097_c1_seq1|7.m.14053	ankyrin repeat domain-containing protein 12
comp21108_c1_seq1|7.m.14129	Zinc finger CCHC domain-containing protein 9
comp21270_c0_seq1|7.m.14823	ionotropic receptor 7c, isoform A
comp21521_c0_seq1|7.m.16108	4-coumarate-CoA ligase-like protein
comp21701_c0_seq1|7.m.16683	P94
comp2170_c0_seq1|7.m.237	rho-gtpase-activating protein
comp22083_c0_seq1|7.m.18300	CG34114, isoform C
comp22212_c0_seq1|7.m.19793	steroid receptor-interacting snf2 domain protein
comp22219_c0_seq1|7.m.19809	piggyBac transposable element-derived protein 3-like
comp22345_c0_seq1|7.m.20371	putative Zinc finger protein 294
comp22386_c0_seq1|7.m.20872	bromodomain-containing protein DDB_G0280777-like
comp22614_c0_seq1|7.m.22537	cellulosome anchoring protein cohesin region
comp22739_c0_seq1|7.m.23016	NA
comp22862_c0_seq1|7.m.23633	collapsin response mediator protein
comp22988_c0_seq1|7.m.24085	putative inorganic phosphate cotransporter-like
comp23111_c1_seq1|7.m.24583	NA

comp24491_c0_seq1|7.m.33997	chascon, isoform A
comp24492_c1_seq1|7.m.34008	NA
comp24699_c1_seq1|7.m.36218	carboxypeptidase D-like
comp24784_c0_seq1|7.m.36944	Dynein intermediate chain, cytosolic
comp2478_c0_seq1|7.m.307	protein four-jointed-like
comp25000_c0_seq1|7.m.39167	cdep, isoform G
comp25140_c0_seq1|7.m.40589	unc-13, isoform C
comp25217_c0_seq1|7.m.41213	tRNA 2-thiocytidine biosynthesis protein ttcA
comp25257_c4_seq1|7.m.41521	endonuclease-reverse transcriptase
comp25300_c0_seq1|7.m.42302	Cad88C CG3389-PA
comp25323_c3_seq1|7.m.42508	Tigger transposable element-derived protein 1
comp25356_c0_seq1|7.m.42836	PREDICTED: uncharacterized protein LOC101740623
comp25405_c0_seq1|7.m.43524	NA
comp25458_c0_seq1|7.m.44666	serine protease precursor
comp25486_c1_seq1|7.m.45430	NA
comp25492_c0_seq1|7.m.45476	Pleckstrin-like proteiny-like domain family B member 2
comp25497_c0_seq1|7.m.45524	Transposable element Tc3 transposase
comp25826_c0_seq1|7.m.45926	Hexokinase type, putative
comp26785_c0_seq1|7.m.46489	ring finger protein
comp27191_c0_seq1|7.m.46559	small optic lobes, isoform C
comp2730_c0_seq1|7.m.364	zinc finger protein 728-like
comp27417_c0_seq1|7.m.46617	vacuolar protein sorting 13D
comp27480_c0_seq1|7.m.46635	DDB1- and CUL4-associated factor 10 homolog isoform X1
comp27554_c0_seq1|7.m.46656	conserved oligomeric Golgi complex subunit 3-like
comp27575_c0_seq1|7.m.46662	argininosuccinate synthase
comp27692_c0_seq1|7.m.46696	S08405hypothetical protein 2 - silkworm transposon mag
comp27701_c0_seq1|7.m.46701	hypothetical protein KGM_20511
comp28088_c0_seq1|7.m.46824	cell division control protein 45 homolog
comp28119_c0_seq1|7.m.46835	reverse transcriptase, putative
comp28234_c0_seq1|7.m.46871	groucho protein
comp2880_c1_seq1|7.m.406	transient receptor potential A1
comp2900_c0_seq1|7.m.412	Alpha-aminoadipic semialdehyde synthase, mitochondrial
comp2965_c0_seq1|7.m.426	NA
comp3161_c0_seq1|7.m.457	PREDICTED: hypothetical protein LOC100572469
comp3287_c0_seq1|7.m.471	vitellogenin, putative
comp32957_c0_seq1|7.m.47102	unkown protein
comp33071_c0_seq1|7.m.47132	probable multidrug resistance-associated protein lethal(2)03659-like
comp33081_c0_seq1|7.m.47134	retinol dehydrogenase 13-like
comp33090_c0_seq1|7.m.47138	NA

comp33094_c0_seq1|7.m.47140	PREDICTED: uncharacterized protein LOC101746815
comp33176_c0_seq1|7.m.47163	GTPase-activating protein and VPS9 domain-containing protein 1-like
comp33230_c0_seq1|7.m.47181	potassium voltage-gated channel subfamily H member 7-like
comp33296_c0_seq1|7.m.47202	TATA-binding protein-associated factor 172-like
comp33297_c0_seq1|7.m.47204	Bloom syndrome protein homolog
comp33528_c0_seq1|7.m.47262	argonaute 3
comp33755_c0_seq1|7.m.47320	WD repeat-containing protein 81-like
comp34142_c0_seq1|7.m.47404	cuticular protein RR-1 motif 46
comp34199_c0_seq1|7.m.47417	sorbitol dehydrogenase
comp3443_c0_seq1|7.m.494	probable multidrug resistance-associated protein lethal(2)03659-like
comp34572_c0_seq1|7.m.47475	PREDICTED: uncharacterized protein LOC101742291
comp34649_c0_seq1|7.m.47488	Lin29, isoform D
comp34655_c0_seq1|7.m.47491	WD repeat-containing protein 16-like
comp34657_c0_seq1|7.m.47492	TBC1 domain family member 19-like
comp34771_c0_seq1|7.m.47503	Limulus clotting factor C
comp34878_c0_seq1|7.m.47530	transcriptional repressor scratch 2-like
comp35038_c0_seq1|7.m.47563	putative zinc finger protein
comp35140_c0_seq1|7.m.47582	putative fatty acyl-CoA reductase CG5065-like
comp35146_c0_seq1|7.m.47587	NA
comp35251_c0_seq1|7.m.47603	reverse transcriptase
comp35480_c0_seq1|7.m.47649	RNA-directed DNA polymerase from mobile element jockey-like
comp35529_c0_seq1|7.m.47654	bipolar kinesin KRP-130-like
comp35742_c0_seq1|7.m.47695	microtubule-associated protein futsch-like
comp3577_c0_seq1|7.m.525	hypothetical protein KGM_10100
comp35963_c0_seq1|7.m.47727	ribosomal protein S6 kinase alpha-3-like
comp36006_c0_seq1|7.m.47736	Down syndrome cell adhesion molecule-like protein Dscam2-like
comp36113_c0_seq1|7.m.47759	NA
comp36119_c0_seq1|7.m.47764	tyrosine-protein kinase-like otk-like
comp36335_c0_seq1|7.m.47796	DC-STAMP domain-containing protein 2
comp36611_c0_seq1|7.m.47849	neuropeptide receptor A19
comp37088_c0_seq1|7.m.47925	RNA-binding protein 26-like
comp3784_c0_seq1|7.m.579	gag-like protein
comp37864_c0_seq1|7.m.48033	UPF0378 protein KIAA0100-like
comp38198_c0_seq1|7.m.48074	post-GPI attachment to proteins factor 2-like
comp38778_c0_seq1|7.m.48139	neuropeptides capa receptor-like
comp39000_c0_seq1|7.m.48169	retroelement polyprotein
comp39055_c0_seq1|7.m.48173	cuticular protein RR-2 motif 143
comp3925_c1_seq1|7.m.608	NGFI-A-binding protein homolog

comp39668_c0_seq1|7.m.48226	hypothetical protein KGM_15935
comp40130_c0_seq1|7.m.48274	A disintegrin and metalloproteinase with thrombospondin motifs 2-like
comp40180_c0_seq1|7.m.48283	reverse transcriptase, putative
comp4070_c0_seq1|7.m.651	NA
comp4117_c0_seq1|7.m.661	ATP dependant DNA helicase PIF1
comp43257_c0_seq1|7.m.48519	NA
comp44046_c0_seq1|7.m.48553	putative DNA helicase
comp4563_c0_seq1|7.m.763	RNA-directed DNA polymerase from mobile element jockey-like
comp47238_c0_seq1|7.m.48692	serine-rich adhesin for platelets-like
comp47355_c0_seq1|7.m.48695	putative endonuclease and reverse transcriptase-like protein
comp47953_c0_seq1|7.m.48709	reverse transcriptase
comp5369_c0_seq1|7.m.1120	remodeling and spacing factor 1-like
comp5580_c0_seq1|7.m.1209	toutatis, isoform A
comp5767_c0_seq1|7.m.1259	Nanos homolog 2
comp806_c0_seq1|7.m.73	cadherin 96Cb, isoform C
comp8200_c0_seq1|7.m.1783	sickie, isoform H
comp8224_c0_seq1|7.m.1796	Protein spaetzle
comp8288_c0_seq1|7.m.1811	probable Rho GTPase-activating protein CG5521-like
comp8687_c0_seq1|7.m.1981	cytochrome P450
comp881_c0_seq1|7.m.87	hypothetical protein KGM_13684
comp8831_c0_seq1|7.m.2051	Uncharacterized ATP-dependent helicase YHR031C
comp8975_c0_seq1|7.m.2147	TATA element modulatory factor
comp9047_c0_seq1|7.m.2185	hypothetical protein TcasGA2_TC016102
comp9267_c0_seq1|7.m.2318	RNA-directed DNA polymerase from mobile element jockey-like
comp9710_c0_seq1|7.m.2718	cuticular protein RR-2 motif 143

Table S6. Annotation of 276 positively selected clusters identified by Ka/Ks

Cluster ID	Description
1062	type i inositol -trisphosphate 5-phosphatase isoform x1
1096	low quality protein: papilin-like
1121	transmembrane protein 68-like
1158	hypothetical protein KGM_17169
1164	guanine nucleotide binding protein
1188	very low-density lipoprotein receptor-like
1212	brain chitinase and chia
1216	hypothetical protein KGM_15439
1237	similar to CG15023
1239	achi protein
1276	rna-binding protein musashi homolog rbp6-like
1279	male-specific lethal 3
1309	hypothetical protein KGM_15578
1332	small heat shock protein
1342	hypothetical protein KGM_08504
1396	upf0585 protein c16orf13-like protein a
1427	n-alpha-acetyltransferase 30-like
1433	selenoprotein m-like
1436	werner helicase interacting protein
146	microsomal triglyceride transfer protein large subunit-like (*Mtp*)
149	n -(beta-n-acetylglucosaminyl)-l-asparaginase
1529	choline-binding surface protein a
1548	serine threonine-protein kinase chk2

1597	hemolymph proteinase 17
1639	hypothetical protein KGM_14984
1645	sideroflexin 1
1648	nad dependent epimerase dehydratase
1658	smc2 protein
1667	inner nuclear membrane protein man1
1690	diphthamide biosynthesis protein 2-like
1693	No hits found
1715	No hits found
1744	nfx1-type zinc finger-containing protein 1-like
1768	pupal cuticle protein precursor
1777	dalr anticodon-binding domain-containing protein 3-like
1794	ecdysteroid 22-kinase
1815	lysophospholipase-like protein 1-like
1827	coagulation factor xi
1837	zinc finger ccch-type with g patch domain-containing
1841	transformation transcription domain-associated
1874	high mobility group
1946	hypothetical protein KGM_00635
1948	iron-sulfur cluster assembly 2 mitochondrial-like
1954	programmed cell death 7
1961	mitochondrial prohibitin complex protein 2
1980	hypothetical protein KGM_07888
2005	carboxyl-terminal pdz ligand of neuronal nitric oxide synthase
2023	endoplasmic reticulum resident protein 29-like
2033	nfx1-type zinc finger-containing protein 1-like

206	myosin-i heavy chain
2062	solute carrier family facilitated glucose transporter member 3-like
2114	kxdl motif-containing protein cg10681-like
2119	hypothetical protein KGM_15216
2120	actin-related protein 10-like
2130	kinase suppressor of ras 2-like
2152	uncharacterized protein LOC101744858
2231	tau-tubulin kinase 1-like
2246	nucleosome-remodeling factor subunit nurf301
2311	folylpolyglutamate mitochondrial-like
2397	nuclear fragile x mental retardation-interacting protein 1
2412	ring finger protein 126-like
2446	superoxide dismutase
2475	putative survivin
2508	hypothetical protein KGM_09242
2562	protein spire-like
2571	hypothetical protein KGM_18645
2577	three prime repair exonuclease 1
2609	mitochondrial cardiolipin hydrolase-like
2621	histone-lysine n-methyltransferase setmar-like
2698	hypothetical protein KGM_08685
2736	exosome complex component mtr3-like
2737	coiled-coil-helix-coiled-coil-helix domain-containing protein mitochondrial-like
2749	ell complex eap30 subunit
2790	atypical protein kinase c

2828	xylulose kinase
2859	an1-type zinc finger protein 6-like isoform x1
2863	uncharacterized protein LOC101737983
288	aminoacylase-1-like
2889	guanylate cyclase 32e-like
296	ras-related protein rab-28
2964	hypothetical protein KGM_14542
2967	nuclear pore complex protein nup205
2971	digestive organ expansion factor homolog
2977	tuberous sclerosis 2 isoform 3
3036	endonuclease-reverse transcriptase
3084	28s ribosomal protein mitochondrial
3135	atpase family aaa domain-containing protein 5-like
314	zinc finger protein 420-like
318	zinc finger protein
3191	calreticulin
3198	hypothetical protein KGM_19674
3230	cdk5 and abl1 enzyme substrate 1-like isoform x3
3257	hypothetical protein KGM_06385
3260	alpha-2-macroglobulin-like protein 1-like
329	hypothetical protein KGM_16230
3300	multiple ankyrin repeats single kh domain
3361	nuclear pore complex protein nup88-like
3399	an1-type zinc finger protein 1-like
3437	z band alternatively spliced pdz-motif protein isoform b
3445	mitotic spindle assembly checkpoint protein mad2a-like

3459	nedd8 ultimate buster 1-like
3505	probable atp-dependent rna helicase dhx35-like
3587	low quality protein: liprin-alpha-1-like
3651	atm interactor
3671	hypothetical protein KGM_15943
3695	uncharacterized protein LOC101744379 isoform X1
3707	rho gtpase activating protein
3760	uncharacterized protein LOC101743259
3801	cirhin isoform x1
3820	luciferin-regenerating enzyme, *regucalcin*
3830	actin-related protein 5-like
3852	outer dense fiber protein 3
3921	bud13 homolog
3939	lipase 3-like
3971	ribonucleases p mrp protein subunit pop1-like
3977	kinesin-like protein kif3c-like
4009	prominin-like isoform 2
4042	leucine-rich transmembrane protein
4044	lon peptidase n-terminal domain and ring finger protein 3-like
4059	zinc finger protein 729-like
4068	chitin deacetylase 1
4116 [a]	ap-1 complex subunit gamma-1
4182	nadh dehydrogenase
4188	transmembrane protein 222
4214	katanin p80 wd40-containing subunit b1
4218	zinc finger protein 91-like

4254	peroxisome assembly protein 12
4317	condensin complex subunit 3-like
4318	nadh dehydrogenase
4323	hypothetical protein KGM_09036
4324	nmda receptor-regulated protein 2
4325	a kinase anchor protein
4343	nuclear pore membrane glycoprotein 210-like
4364	dna (cytosine-5)-methyltransferase -like
4381	p94-like protein
4445	u4 tri-snrnp-associated protein 1
4447	zinc finger and scan domain-containing protein 10-like isoform x3
4472	nicotinate phosphoribosyltransferase-like isoform x2
4484	domain-containing protein 7
4535	ring finger and spry domain-containing protein 1-like isoform x1
4602	uncharacterized protein LOC101738037
4606	activin receptor type-2b-like
4607	translocation associated membrane protein
4618	probable rna polymerase ii nuclear localization protein slc7a6os-like
462	tbc1 domain family member 13-like
4690	alpha-2-macroglobulin receptor-associated
4699	glutathione s-transferase omega 1
471	hypothetical protein KGM_12191
472	replication protein a 32 kda subunit
4734	hypothetical protein KGM_17470
4736	anaphase-promoting complex subunit 2
4755	colorectal mutant cancer protein

4779	hypothetical protein KGM_20226
4798	e3 ubiquitin-protein ligase ubr7-like
4804	6-phosphogluconolactonase-like
481	endoribonuclease dcr-1
4835	centrosomal protein 350kda
488	juvenile hormone esterase binding protein
4942	ubiquitin carboxyl-terminal hydrolase 10
4945	chitin synthase
4952	peptidyl-prolyl cis-trans isomerase d
4958	serine threonine-protein phosphatase 4 regulatory subunit 3-like
4964	apolipoprotein o-like
5006	pseudouridylate synthase
5053	facilitated trehalose transporter tret1-like
5172	acyl- synthetase family member mitochondrial-like isoform 2
5225	alkaline tissue-nonspecific isozyme-like
5246	hypothetical protein KGM_20043
5252	zgc:173726 protein
5263	cytochrome b-c1 complex subunit mitochondrial-like
5266	mannose-1-phosphate guanyltransferase beta-like
5293	serine threonine-protein kinase gd17699-like
5298	mrna cap-binding protein eif4e
5359	c2 domain-containing protein 5-like
5362	serine proteinase-like protein 1
5380	hypothetical protein KGM_00432
5384	acylphosphatase-1-like
5395	calcium homeostasis endoplasmic reticulum isoform x1

5437	ap-3 complex subunit delta-like
5457	rna-binding protein nob1-like
5518 [b]	lachesin-like, DIP-alpha
5555	hypothetical protein KGM_10100
557	tenascin-like isoform x2
5614	c-Cbl-associated protein isoform A
5646	g patch domain and ankyrin repeat-containing protein 1 homolog
5666	uncharacterized protein LOC101739175, partial
5692	nadph:quinone reductase
5716	ankyrin unc44
5733	bindin, partial
5736	cytosolic fe-s cluster assembly factor nubp2 homolog
5749	hypothetical protein KGM_01384
5770 [b]	hypothetical protein KGM_13863, stops
5789	fanconi anemia group d2
5795	hypothetical protein KGM_05133
5804	hypothetical protein KGM_14348
5825	defective proboscis extension response
5826	zinc finger protein 77
5827	dna replication factor cdt1
5841	probable maleylacetoacetate isomerase 2-like
5846	transcriptional regulator atrx homolog
585	protein n-terminal glutamine amidohydrolase-like
5894	No hits found
5955	spermatogenesis-associated protein 20-like
598	protein transport protein sec24c-like

5990	anaphase-promoting complex subunit 5-like
5991	wd repeat-containing protein cg11141-like
6085	probable cation-transporting atpase 13a3-like
6125	double-stranded rna-specific editase adar-like
6161	zinc finger ccch-type with g patch domain-containing
6168	zinc finger protein
6190	iron-sulfur cluster assembly 1-like mitochondrial
6212	replication protein a3
6247	zinc finger protein
6266	tudor domain-containing protein 1-like
6279	alkylated dna repair protein alkb homolog 8-like
6289	axoneme-associated protein
6312	inositol-tetrakisphosphate 1-kinase-like
6319	ras gtp exchange son of sevenless
6340	nucleolar protein 56-like
6397	cytoplasmic dynein 2 heavy chain 1-like
6443	encore protein
6485	exosome complex component rrp43-like
6511	hypothetical protein KGM_08170
6549	piggybac transposable element-derived protein 3-like
6610 [b]	allatostatin neuropeptide precursor (*AstA*)
6622	zinc finger protein 135-like
664	inactive rhomboid protein 1- partial
6651	ribosomal rna processing protein 1 homolog
6659	beat protein
6665	inhibition of apoptosis protein 2

6693	hypothetical protein KGM_15276
682	mitochondrial ribosomal protein s11
6884	ring finger protein 168
6911	odorant binding protein
6939	neuroblastoma-amplified sequence
7	glutamyl-trna amidotransferase subunit a
72	cytoplasmic dynein heavy chain 1b
7265	valyl-trna synthetase
7344	cleavage stimulation factor subunit 1-like
7346	c-type lectin 27kd
7355	inter-alpha-trypsin inhibitor heavy chain H4-like
7453	substrate- ligase
7487	paternally expressed 3
7549	metal-response element-binding transcription factor 2
7569	disks large-associated protein 5-like isoform x4
7599	regulator of g-protein signaling loco-like
7615	hypothetical protein KGM_00190
7655	flavin-dependent monooxygenase fmo3b
7710	histone-lysine n-methyltransferase ehmt1-like
7734	wd repeat-containing protein 18-like
7770	hypothetical protein KGM_13512
7788	zinc finger protein 155-like
7807 [a]	map7 domain-containing protein 2 isoform x9, dan
7811	hypothetical protein KGM_15082
783	birt-hogg-dube homolog
7859	stam-binding a-like

7910	mucin 68d
793	hypothetical protein KGM_07922, *crim*
7944	inactive ubiquitin carboxyl-terminal hydrolase 53-like isoform x2
7952	dentin sialophosphoprotein
7982	upf0501 protein kiaa1430-like protein
8033	inositol -trisphosphate receptor-like
8040	translation initiation factor 2 gamma subunit
8042	low quality protein: e3 ubiquitin-protein ligase cbl-like
8084	hypothetical protein KGM_19303
8114	glutaminyl-trna synthetase
8122	actin-binding protein ipp
815	uncharacterized protein LOC101740198
840	prolyl 3-hydroxylase 2-like
853	syntaxin-18-like
915	protein transport protein sec31a
93	crag protein
944	transcription factor sp3-like
945	glycerol kinase
972	adenine phosphoribosyltransferase
987	putative N-acetyllactosaminide beta-1,3-N-acetylglucosaminyltransferase

[a] indicates vision-related genes.

[b] indicates genes commonly upregulated in heads.

Table S7. Counts of clusters containing comparisons with a Ka/Ks ratio above one

Numbers of Comparisons with a Ka/Ks >1	Cluster Counts
1	163
2	37
3	26
4	9
5	14
6	7
7	7
8	0
9	2
10	1
11	2
12	1
13	5
14	1
15	0
16	0
17	0
18	0
19	1
More than 19	0

Table S8. GO-term enrichment of conserved clusters containing at least a comparison with a Ka/Ks ratio above one

GO ID	Category	Type	P-value
GO:0070192	chromosome organization involved in meiotic cell cycle	P	0.001
GO:0009226	nucleotide-sugar biosynthetic process	P	0.005
GO:0018022	peptidyl-lysine methylation	P	0.005
GO:0034968	histone lysine methylation	P	0.005
GO:0016571	histone methylation	P	0.009
GO:0051276	chromosome organization	P	0.010
GO:0006325	chromatin organization	P	0.011
GO:0016811	hydrolase activity, acting on carbon-nitrogen (but not peptide) bonds, in linear amides	F	0.012
GO:0048593	camera-type eye morphogenesis	P	0.013
GO:0060041	retina development in camera-type eye	P	0.013
GO:0060042	retina morphogenesis in camera-type eye	P	0.013
GO:0090307	mitotic spindle assembly	P	0.013
GO:0042805	actinin binding	F	0.013
GO:0016226	iron-sulfur cluster assembly	P	0.013
GO:0051371	muscle alpha-actinin binding	F	0.013
GO:0051393	alpha-actinin binding	F	0.013
GO:0031163	metallo-sulfur cluster assembly	P	0.013
GO:0007076	mitotic chromosome condensation	P	0.014
GO:0016810	hydrolase activity, acting on carbon-nitrogen (but not peptide) bonds	F	0.014
GO:0006479	protein methylation	P	0.016

GO:0008213	protein alkylation	P	0.016
GO:0008270	zinc ion binding	F	0.016
GO:1901071	glucosamine-containing compound metabolic process	P	0.017
GO:0006040	amino sugar metabolic process	P	0.017
GO:0006030	chitin metabolic process	P	0.017
GO:0044815	DNA packaging complex	C	0.019
GO:0045132	meiotic chromosome segregation	P	0.019
GO:0016569	covalent chromatin modification	P	0.022
GO:0016570	histone modification	P	0.022
GO:0048565	digestive tract development	P	0.025
GO:0009225	nucleotide-sugar metabolic process	P	0.025
GO:0000796	condensin complex	C	0.025
GO:0002521	leukocyte differentiation	P	0.025
GO:0000793	condensed chromosome	C	0.026
GO:0007498	mesoderm development	P	0.026
GO:0005694	chromosome	C	0.027
GO:0055123	digestive system development	P	0.030
GO:0051536	iron-sulfur cluster binding	F	0.036
GO:0051540	metal cluster binding	F	0.036
GO:0006022	aminoglycan metabolic process	P	0.038
GO:0005201	extracellular matrix structural constituent	F	0.040
GO:0006801	superoxide metabolic process	P	0.040
GO:0043010	camera-type eye development	P	0.040
GO:0043094	cellular metabolic compound salvage	P	0.040
GO:1903047	mitotic cell cycle process	P	0.041

GO:0140014	mitotic nuclear division	P	0.044
GO:0004518	nuclease activity	F	0.050

P, F and C stand for biological process, molecular function and cellular component, respectively.

Table S9. Annotation of positively selected clusters identified by CodeML

Cluster ID	Tested Branch	Annotation
7***[b, c]	*H. cydno*	Glutamyl-tRNA(Gln) amidotransferase subunit A [*Culex quinquefasciatus*]
72***[a, b, c]	*H. sara*	Cytoplasmic dynein heavy chain, putative [*Ixodes scapularis*]
146***[a]	*H. doris*	Microsomal triglyceride transfer protein large subunit-like [*Bombyx mori*]
149**[b, c]	*H. cydno*	l-asparaginase [*Danaus plexippus*]
488***	*D. iulia*	Juvenile hormone esterase binding protein [*Manduca sexta*]
557*[b, c]	*H. hortense*	Possible antimicrobial peptide [*Bombyx mori*]
557**[b, c]	*H. melpomene*	
664*[b]	*H. erato*	Inactive rhomboid protein 1-like [*Bombyx mori*]
664***[b]	*D. iulia*	
682***	*H. hecale*	28S ribosomal protein S11, mitochondrial-like [*Bombyx mori*]
783***[b, c]	*D. iulia*	Folliculin-like [*Bombyx mori*]
793*[a, b, c]	*H. erato*	Hypothetical protein KGM_07922 [*Danaus plexippus*]
815*[b, c]	*H. sara*	Uncharacterized protein LOC101740198 [*Bombyx mori*]
945**[b, c]	*H. sara*	Glycerol kinase [*Bombyx mori*]
972***[b]	*H. hecale*	Adenine phosphoribosyltransferase-like [*Bombyx mori*]
1121*[b, c]	*H. cydno*	Transmembrane protein 68-like, partial [*Bombyx mori*]
1164***[b, c]	*H. erato*	Guanine nucleotide binding protein [*Bombyx mori*]
1212***[b, c]	*D. iulia*	Probable chitinase 2-like [*Bombyx mori*]
1396***[b, c]	*H. hecale*	UPF0585 protein CG18661-like isoform X1 [*Bombyx mori*]

1427***[b, c]	*H. doris*	Putative acetyltransferase C complex catalytic subunit Mak3 [*Danaus plexippus*]
1815**	*H. sara*	Hypothetical protein KGM_10900 [*Danaus plexippus*]
1815*	*H. melpomene*	
1841*[b,]	*H. doris*	Putative transformation/transcription domain-associated protein isoform 1 [*Danaus plexippus*]
1946*[b, c]	*D. iulia*	Hypothetical protein KGM_00635 [*Danaus plexippus*]
2119***[b, c]	*H. erato*	Hypothetical protein KGM_15216 [*Danaus plexippus*]
2120*[a, b, c]	*cydno - melpomene - hecale*	Actin-related protein 10-like [*Bombyx mori*]
2120*[a, b, c]	*D. iulia*	
2412*[b, c]	*cydno - melpomene*	Zinc finger protein 364 [*Danaus plexippus*]
2412*[b, c]	*D. iulia*	
2475**	*H. hortense*	Putative survivin [*Danaus plexippus*]
2475**	*cydno - melpomene - hecale*	
2475***	*H. cydno*	
2475**	*H. erato*	
2508***	*D. iulia*	Hypothetical protein KGM_09242 [*Danaus plexippus*]
2698*[b, c]	*D. iulia*	Hypothetical protein KGM_08685 [*Danaus plexippus*]
2736**	*H. hecale*	mRNA transport regulator 3 [*Danaus plexippus*]
2736*	*D. iulia*	
2737*[b, c]	*hortense - sara*	Hypothetical protein KGM_09857 [*Danaus plexippus*]
2737*[b, c]	*H. sara*	
2737*[b, c]	*H. erato*	
2737*[b, c]	*H. melpomene*	
2863***[b, c]	*cydno - melpomene - hecale*	Actin cytoskeleton-regulatory complex protein PAN1-like isoform X3 [*Ceratitis capitata*]
2863***[b, c]	*cydno - melpomene*	

2964*** [a, b, c]	*H. melpomene*	Hypothetical protein KGM_14542 [*Danaus plexippus*]
2977*	*H. melpomene*	Putative tuberous sclerosis 2 isoform 3 [*Danaus plexippus*]
2977***	*D. iulia*	
3036** [a, b, c]	*H. hecale*	Endonuclease-reverse transcriptase [*Danaus plexippus*]
3135*** [b, c]	*D. iulia*	ATPase family AAA domain-containing protein 5 [*Tribolium castaneum*]
3260*** [b, c]	*H. sara*	Alpha-2-macroglobulin-like protein 1-like [*Bombyx mori*]
3399* [b, c]	*H. cydno*	Hypothetical protein KGM_00143 [*Danaus plexippus*]
3651* [b, c]	*H. melpomene*	Hypothetical protein KGM_16145 [*Danaus plexippus*]
3820** [b, c]	*H. sara*	Luciferin regenerating enzyme [*Danaus plexippus*]
3820*** [b, c]	*D. iulia*	
4042*** [a, b, c]	*H. melpomene*	Leucine-rich repeat G protein-coupled receptor precursor [*Bombyx mori*]
4068* [a, b, c]	*D. iulia*	Serpentine [*Papilio polytes*]
4214*** [b, c]	*H. sara*	Katanin p80 WD40 repeat-containing subunit B1-like isoform X1 [*Bombyx mori*]
4218** [b, c]	*D. iulia*	Zinc finger protein 91-like [*Bombyx mori*]
4323*** [a, b, c]	*hortense - sara - erato*	Hypothetical protein KGM_09036 [*Danaus plexippus*]
4325*** [a, b, c]	*D. iulia*	Putative a kinase anchor protein [*Danaus plexippus*]
4472** [b, c]	*H. sara*	Nicotinic acid phosphoribosyltransferase [*Danaus plexippus*]
4484*	*H. hecale*	Pla2g4b [*Danaus plexippus*]
4607*** [b, c]	*D. iulia*	Translocation associated membrane protein [*Papilio polytes*]
4690* [b, c]	*H. hortense*	Alpha-2-macroglobulin receptor-associated protein-like isoform X1 [*Bombyx mori*]
4690*** [b, c]	*H. hecale*	

4964***[b]	*H. melpomene*	Hypothetical protein KGM_14582 [*Danaus plexippus*]
5246***[b, c]	*H. melpomene*	Myosin regulatory light chain 2 [*Camponotus floridanus*]
5266**[b]	*D. iulia*	Mannose-1-phosphate guanyltransferase beta-like [*Bombyx mori*]
5298***[b, c]	*H. hecale*	Eukaryotic initiation factor 4E [*Papilio xuthus*]
5749**[b, c]	*D. iulia*	Hypothetical protein KGM_01384 [*Danaus plexippus*]
5789***[b, c]	*H. hortense*	Fanconi anemia, complementation group D2 [*Bombyx mori*]
5789***[b, c]	*H. sara*	
5789***[b, c]	*H. melpomene*	
5955**[a, b, c]	*H. melpomene*	Spermatogenesis-associated protein 20-like [*Bombyx mori*]
5991***	*cydno - melpomene*	WD repeat-containing protein CG11141-like [*Bombyx mori*]
5991***	*H. melpomene*	
6161**[b, c]	*D. iulia*	Zinc finger CCCH-type with G patch domain-containing protein-like [*Bombyx mori*]
6168*[b, c]	*D. iulia*	Putative zinc finger protein [*Danaus plexippus*]
6247*[b, c]	*H. hortense*	Putative zinc finger protein [*Danaus plexippus*]
6279*[b]	*H. hecale*	Alkylated DNA repair protein alkB homolog 8-like [*Bombyx mori*]
6312*	*hortense - sara*	Putative inositol 1,3,4-triphosphate 5/6 kinase [*Danaus plexippus*]
6312*	*H. hortense*	
6312*	*cydno - melpomene*	
6312*	*H. sara*	
6443***[b, c]	*D. iulia*	Encore protein [*Danaus plexippus*]
6610***[b, c]	*H. cydno*	Helicostatins [*Danaus plexippus*]
6665**[b, c]	*H. erato*	Survivin-2 [*Bombyx mori*]
6884*[b, c]	*H. erato*	Putative ring finger protein 168 [*Danaus plexippus*]

6939* [b, c]	*H. erato*	Putative neuroblastoma-amplified protein [*Danaus plexippus*]
6939*** [b, c]	*H. melpomene*	
7265** [b, c]	*H. hortense*	Valyl-tRNA synthetase [*Danaus plexippus*]
7346*** [b, c]	*H. hortense*	C-type lectin 27kD [*Papilio polytes*]
7569* [b, c]	*H. erato*	Disks large-associated protein 5-like isoform X4 [*Bombyx mori*]
7710** [b, c]	*H. hecale*	Histone-lysine N-methyltransferase EHMT1-like [*Bombyx mori*]
7734* [b, c]	*H. cydno*	WD repeat-containing protein 18-like [*Bombyx mori*]
7734** [b, c]	*H. melpomene*	
7788** [b, c]	*H. melpomene*	Zinc finger protein 155-like [*Bombyx mori*]
7910** [a, b, c]	*cydno - melpomene*	Microtubule-associated protein futsch isoform X3 [*Apis mellifera*]
7944*** [a]	*H. melpomene*	Transcriptional repressor p66 alpha-like [*Bombyx mori*]
8122*** [c]	*H. melpomene*	Actin-binding protein ipp [*Danaus plexippus*]

* indicates *P*-value < 0.5

** indicates *P*-value < 0.01

*** indicates *P*-value < 0.001

[a] indicates the cluster is also identified by GA-branch.

[b] indicates the cluster is also supported by comparing random site models M7 and M8.

[c] indicates the cluster is also supported by comparing random site models M8a and M8.

Table S10. Annotation of positively selected clusters identified by GA-branch

Cluster ID	Branch with Probability > 80%	Annotation
72[a]	*H. hecale*	Cytoplasmic dynein heavy chain [*Ixodes scapularis*]
146[a]	*H. hortense*	Microsomal triglyceride transfer protein large subunit-like [*Bombyx mori*]
318	*H. cydno*	Putative zinc finger protein [*Danaus plexippus*]
598	*H. cydno*	Protein transport protein Sec24C-like [*Apis mellifera*]
793[a]	*H. hortense*	Crimpled-like protein [*Microplitis demolitor*]
1948	*H. melpomene*	Iron-sulfur assembly protein IscA-like 2, mitochondrial-like [*Bombyx mori*]
1954	*H. cydno*	Putative programmed cell death 7 [*Danaus plexippus*]
1980	*H. melpomene*<br>*cydno - melpomene*	Hypothetical protein KGM_07888 [*Danaus plexippus*]
2120[a]	*H. melpomene*	Actin-related protein 10-like [*Bombyx mori*]
2767	*H. cydno*	NACHT and WD repeat domain-containing protein 1-like isoform 1 [*Strongylocentrotus purpuratus*]
2859	*H. hortense*<br>*H. melpomene*	Hypothetical protein KGM_11223 [*Danaus plexippus*]
2964[a]	*H. melpomene*<br>*H. cydno*	Hypothetical protein KGM_14542 [*Danaus plexippus*]
3036[a]	*H. doris*	Endonuclease-reverse transcriptase [*Danaus plexippus*]
3801	*H. melpomene*	Cirhin-like [*Bombyx mori*]
3830	*H. cydno*	Actin-related protein 5-like [*Bombyx mori*]
4042[a]	*H. cydno*	Leucine-rich repeat G protein-coupled receptor precursor [*Bombyx mori*]
4068[a]	*H. hecale*	Chitin deacetylase 1 [*Papilio xuthus*]
4323[a]	*H. cydno*<br>*H. melpomene*	Hypothetical protein KGM_09036 [*Danaus plexippus*]<br>Hypothetical protein KGM_09036 [*Danaus plexippus*]

4325[a]	*H. sara*	A-kinase anchor protein 1, mitochondrial-like isoform X3 [*Bombyx mori*]
4958	*H. melpomene*	Serine/threonine-protein phosphatase 4 regulatory subunit 3-like [*Nasonia vitripennis*]
5172	*H. cydno*	Acyl-CoA synthetase family member 3, mitochondrial-like [*Bombyx mori*]
5225	*H. cydno*<br>*H. melpomene*	Alkaline phosphatase, tissue-nonspecific isozyme-like [*Bombyx mori*]
5252	*H. melpomene*	Putative zinc finger protein 91-like protein [*Danaus plexippus*]
5384	*H. doris*	Acylphosphatase-2-like isoform X1 [*Bombyx mori*]
5692	*H. hortense*	Quinone oxidoreductase-like *[Bombyx mori*]
5955[a]	*H. cydno*	Spermatogenesis-associated protein 20-like [*Bombyx mori*]
7811	*H. melpomene*	Regulation of enolase 1 [*Pseudomonas syringae*]
7944[a]	*H. melpomene*	Transcriptional repressor p66 alpha-like [*Bombyx mori*]
7910[a]	*H. sara*	Hypothetical protein KGM_21558 [*Danaus plexippus*]

[a] indicates the cluster is also identified by CodeML.

Table S11. Significant sites under positive selection in candidate clusters identified by CodeML

Cluster ID	Tested Branch	Positive sites for tested branch
7***	*H. cydno*	419**, 423*
72***[a]	*H. sara*	92**, 93*, 94**
146***[a]	*H. doris*	126*
149**	*H. cydno*	32**
488***	*D. iulia*	19*, 20**, 22**, 28*, 30*, 31*, 32*
557*	*H. hortense*	NA
557**	*H. melpomene*	104*
664*	*H. erato*	34**, 35*, 40**, 41*, 43*
664***	*D. iulia*	2**, 3**, 4**, 5**
682***	*H. hecale*	38*, 39**, 42**
783***	*D. iulia*	152*, 153**, 155**
793* [a]	*H. erato*	NA
815*	*H. sara*	NA
945**	*H. sara*	1**
972***	*H. hecale*	111**, 114**
1121*	*H. cydno*	2**
1164***	*H. erato*	1**, 2*, 4**, 5*
1212***	*D. iulia*	162*, 163*, 167**
1396***	*H. hecale*	54**, 57*
1427***	*H. doris*	238*
1815**	*H. sara*	54*

1815*	*H. melpomene*	43*
1841*	*H. doris*	1**, 3*
1946*	*D. iulia*	NA
2119***	*H. erato*	3*, 4*
2120* [a]	*cydno - melpomene-hecale*	NA
2120* [a]	*D. iulia*	NA
2412*	*cydno - melpomene*	NA
2412*	*D. iulia*	104*, 108*
2475**	*H. hortense*	NA
2475**	*cydno - melpomene - hecale*	NA
2475***	*H. cydno*	NA
2475**	*H. erato*	NA
2508***	*D. iulia*	26*, 27**, 29**
2698*	*D. iulia*	29*, 57*
2736**	*H. hecale*	NA
2736*	*D. iulia*	NA
2737*	*hortense - sara*	NA
2737*	*H. sara*	NA
2737*	*H. erato*	NA
2737*	*H. melpomene*	NA
2863***	*cydno - melpomene - hecale*	NA
2863***	*cydno - melpomene*	NA
2964*** [a]	*H. melpomene*	3*, 5**, 6**, 7**, 8*, 11**, 12**, 15*, 16**, 20*, 21**, 22**

2977*	*H. melpomene*	2*
2977***	*D. iulia*	71**, 72*, 73**, 75**, 81**, 82**, 84**, 85**, 88**, 89**, 90**, 91**, 92**, 93*, 94**
3036** [a]	*H. hecale*	21**, 25**, 26*, 27*, 28**, 29**, 30**, 31**, 32**, 52*, 58**
3135***	*D. iulia*	59**
3260***	*H. sara*	59*
3399*	*H. cydno*	34*
3651*	*H. melpomene*	33*, 51**, 59*
3820**	*H. sara*	49**, 50*
3820***	*D. iulia*	17**, 18*, 19*
4042*** [a]	*H. melpomene*	81**, 82**, 83**, 84*, 85*
4068*[a]	*D. iulia*	19**, 100**, 103**, 105**, 107**, 108**
4214***	*H. sara*	386*
4218**	*D. iulia*	7**, 15**, 16**, 22*
4323*** [a]	*hortense - sara - erato*	NA
4325*** [a]	*D. iulia*	20**, 22**, 23**, 24*, 25**, 26**, 27**, 28**, 29**, 30**, 33*, 34*, 51*, 62**, 63*, 64*, 66**
4472**	*H. sara*	54**
4484*	*H. hecale*	NA
4607***	*D. iulia*	1*, 4**, 153*
4690*	*H. hortense*	33**
4690***	*H. hecale*	193*, 195**
4964***	*H. melpomene*	191**, 194**, 196**, 197**, 199**, 201**, 204**, 206**, 208**, 210**, 211*, 212**, 213**, 217*, 220**, 222**, 224**, 227**, 228**, 230*, 232**

5246***	*H. melpomene*	4*, 6**, 8**, 9**, 10**, 14**, 16**
5266**	*D. iulia*	NA
5298***	*H. hecale*	1*, 3*, 5**, 6**, 8**, 9**, 10*, 11*, 16*
5749**	*D. iulia*	46*, 48*, 50**, 52**, 53*
5789***	*H. hortense*	NA
5789***	*H. sara*	1**, 2**, 3**, 5**, 65*, 69*
5789***	*H. melpomene*	NA
5955** [a]	*H. melpomene*	472**
5991***	*cydno - melpomene*	NA
5991***	*H. melpomene*	NA
6161**	*D. iulia*	3*
6168*	*D. iulia*	6*
6247*	*H. hortense*	47*
6279*	*H. hecale*	93*
6312*	*hortense-sara*	NA
6312*	*H. hortense*	NA
6312*	*cydno - melpomene*	NA
6312*	*H. sara*	NA
6443***	*D. iulia*	2**, 3*, 5**
6610***	*H. cydno*	156**, 157**, 158**, 159*, 160**, 161**, 162**
6665**	*H. erato*	14*
6884*	*H. erato*	107**
6939*	*H. erato*	NA
6939***	*H. melpomene*	1*, 2*, 4*, 5*, 6**, 7**, 8**, 9**, 11*, 12**, 13**, 15*, 17*, 19**, 20*, 23**, 24**, 27*, 28*, 32**, 33*, 34**, 36**, 37**, 39**, 40*, 41**, 42*, 44*, 45*, 46*, 47**, 48**, 50*, 51*, 52**

7265**	*H. hortense*	NA
7346***	*H. hortense*	1**, 2**
7569*	*H. erato*	67*
7710**	*H. hecale*	72*, 74**. 75**
7734*	*H. cydno*	1*
7734**	*H. melpomene*	38**
7788**	*H. melpomene*	103**
7910** [a]	*cydno - melpomene*	NA
7944***[a]	*H. melpomene*	2**, 3**, 4**, 5**, 6*, 8*, 9**, 10**, 11**
8122***	*H. melpomene*	154*

* indicates a probability in between 95% and 99%.

** indicates a probability above 99%.

[a] indicates the cluster is also identified by GA-branch.

Table S12. Expression data of qRT-PCR candidate genes

Cluster ID	Gene Name	modENCODE Head-specific Expression Level (*D. melanogaster*)								
		Virgin Female			Mated Female			Mated Male		
		1-day	4-day	20-day	1-day	4-day	20-day	1-day	4-day	20-day
146	*Mtp*	moderately high	moderate	moderately high	moderate	moderate	moderately high	moderate	moderate	moderately high
793	*crim*	moderately high	moderate	moderate	moderate	moderate	moderate	moderately high	moderately high	moderately high
3820	*regucalcin*	high	high	very high	high	high	high	high	very high	very high
6610	*AstA*	moderate	moderate	moderate	moderate	moderate	moderate	moderate	moderately high	moderately high

Table S13. Annotation of qRT-PCR candidate genes

Cluster ID	Gene Name		GO Terms (*D. melanogaster*)	
			Based on Experimental Evidence	Based on Predictions or Assertions
146	*Mtp*	**Molecular Function**	phosphatidylcholine transporter activity	NA
		**Biological Process**	dendrite morphogenesis, lipoprotein metabolic process, lumen formation (open tracheal system), synaptic target recognition	lipid transport
		**Cellular Component**	endomembrane system	NA
793	*crim*	**Molecular Function**	NA	NA
		**Biological Process**	liquid clearance (open tracheal system), regulation of tube size (open tracheal system), septate junction assembly	NA
		**Cellular Component**	septate junction	NA
3820	*regucalcin*	**Molecular Function**	NA	calcium ion binding, gluconolactonase activity
		**Biological Process**	multicellular organism reproduction	L-ascorbic acid biosynthetic process
		**Cellular Component**	extracellular region, extracellular space	NA
6610	*AstA*	**Molecular Function**	neuropeptide receptor binding	hormone activity, neuropeptide hormone activity, signaling receptor binding
		**Biological Process**	neuropeptide signaling pathway	negative regulation of juvenile hormone biosynthetic process, neuropeptide signaling pathway
		**Cellular Component**	extracellular space	extracellular space
