# Supplementary material for: Comparative Transcriptomics Provides Insights into Reticulate and Adaptive Evolution of a Butterfly Radiation
Source: Genome Biol Evol. 2019 Sep 13;11(10):2963–75. doi: 10.1093/gbe/evz202 (PMC6821300; doi:10.1093/gbe/evz202)
Supplement: evz202_Supplementary_Data [file evz202_supplementary_data.zip › Additional_file_2.pdf]

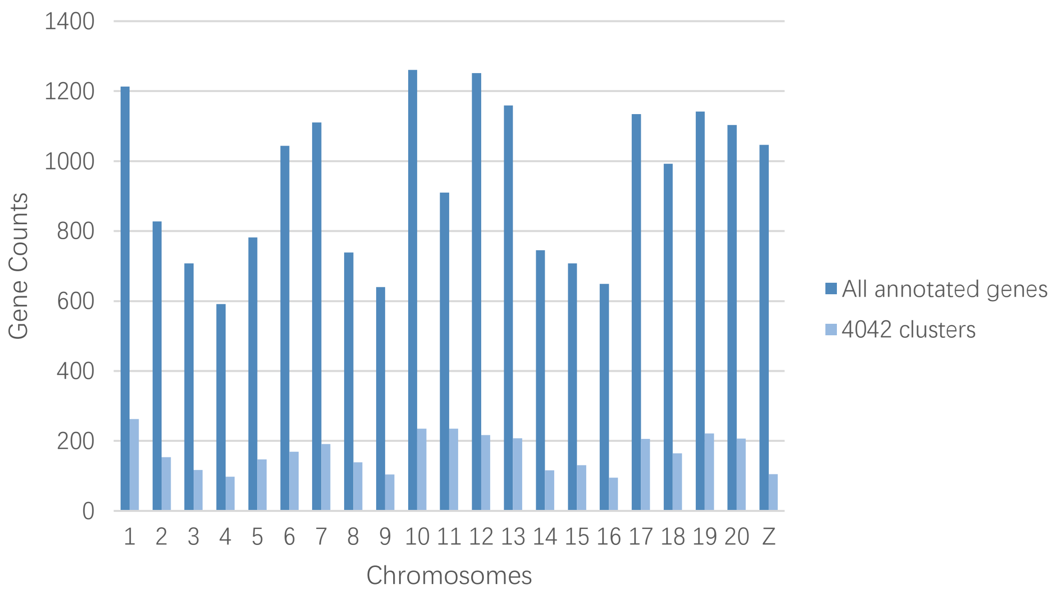

Figure S1. Chromosomal-level distributions of all annotated genes and 4042 clusters.

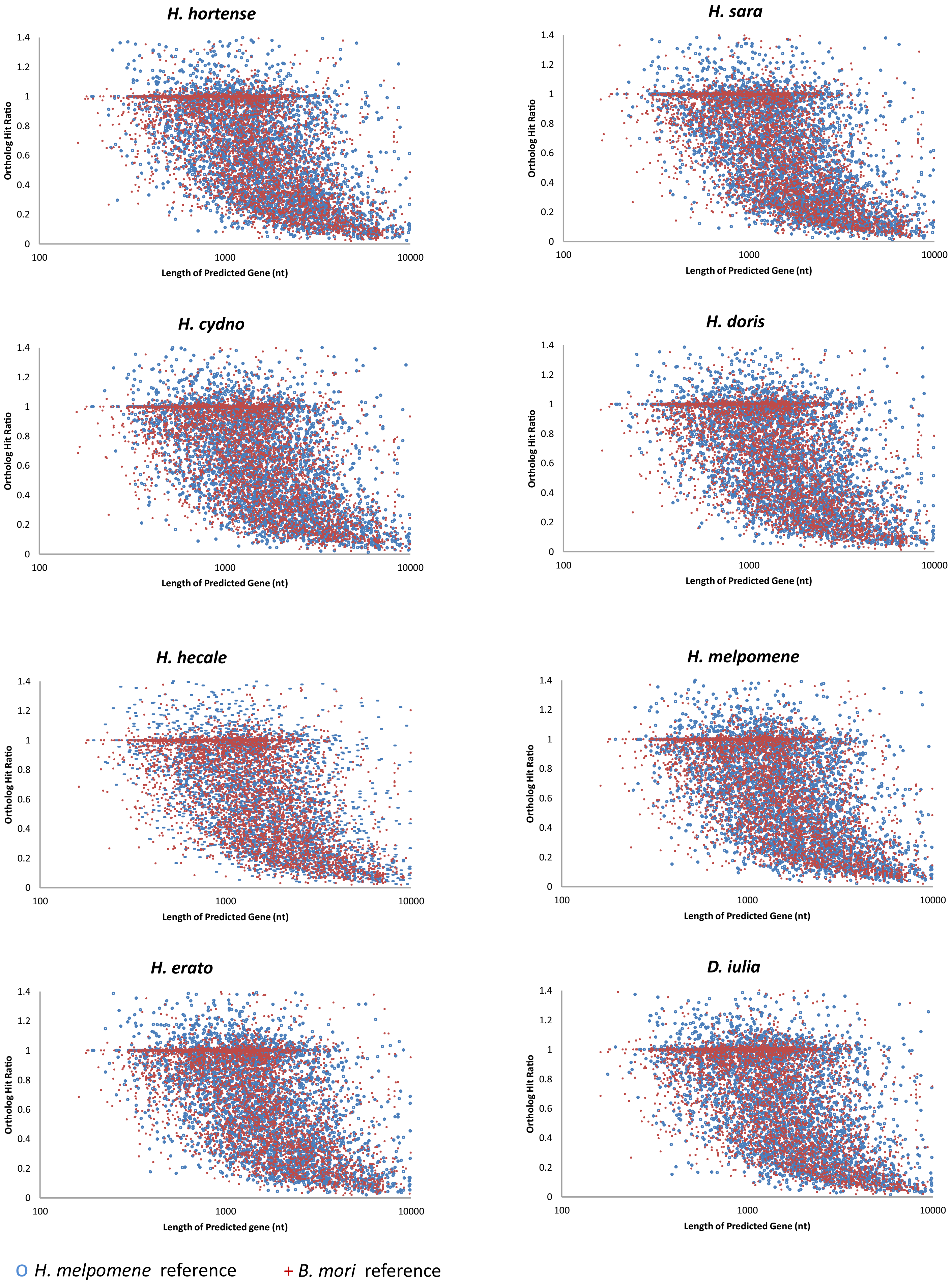

Figure S2. Orthologue hit ratios. Ortholog hit ratios are performed for eight assembled transcriptomes, referring to *H. melpomene* and *B. mori*, separately.

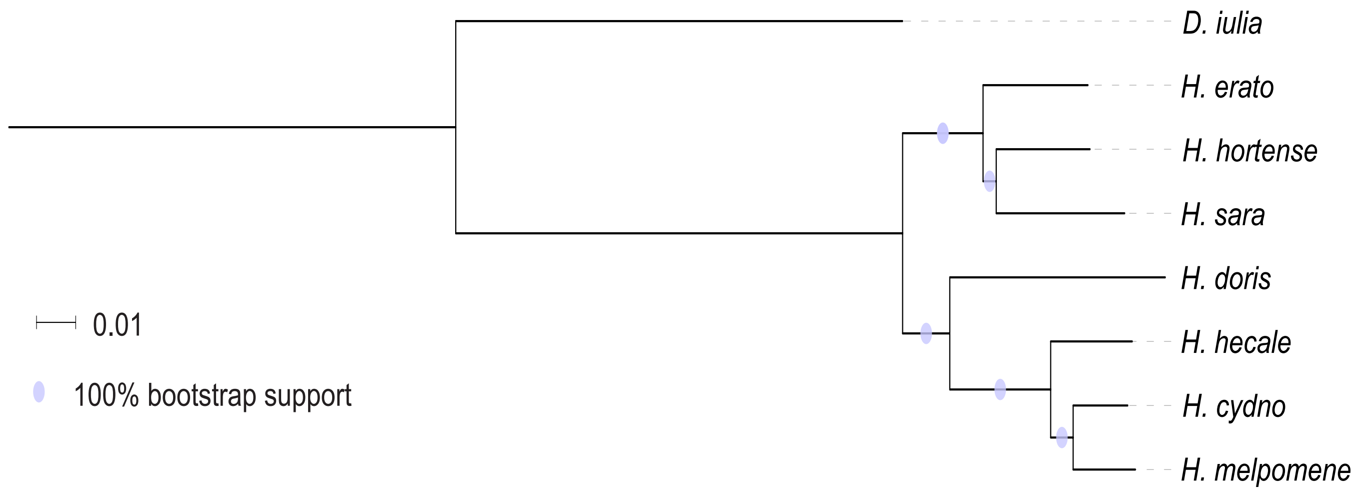

Figure S3. Transcriptome (with positively selected orthologs excluded)-based phylogeny of *Heliconius* species. The maximum likelihood phylogenetic tree is constructed based on conserved CDS with 276 positively selected clusters removed. The scale bars represent the percentage of substitutes per site.

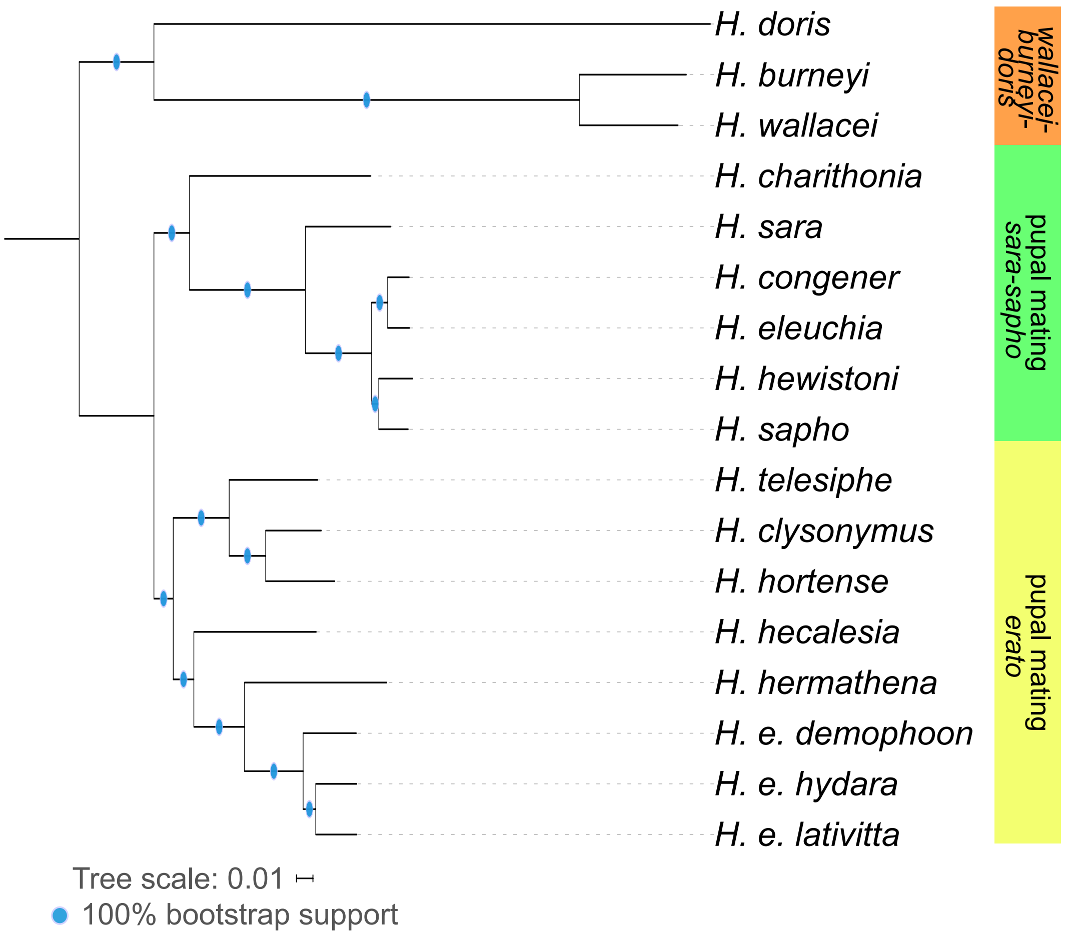

Figure S4. Genome-wide phylogeny of *Heliconius* species. The maximum-likelihood phylogenetic tree is constructed according to genome-wide SNP data with different clades labeled in different colors. The scale bar represents the percentage of substitutions per site.

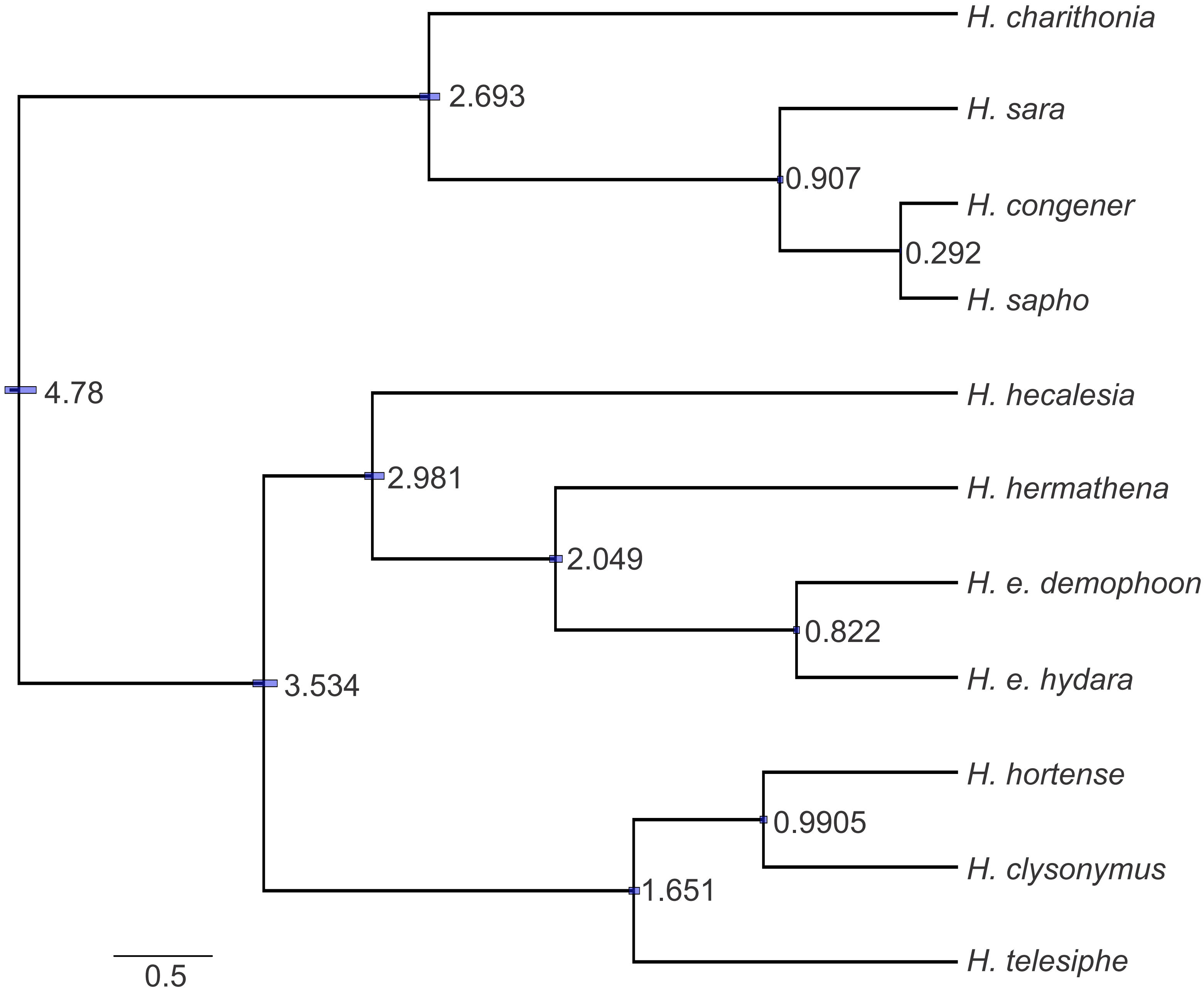

Figure S5. Time-calibrated genome-wide phylogeny of *Heliconius* species. A maximum-likelihood phylogenetic tree is constructed for 11 samples with calibrated nodes, with numbers representing million years and blue bars representing 95% confidence intervals. Divergence times were calibrated using a fast Bayesian approach according to the split time of *H. hecalesia* and *H. erato* (about 4.5 Mya, ranging from 2.7 to 6.3 Mya).

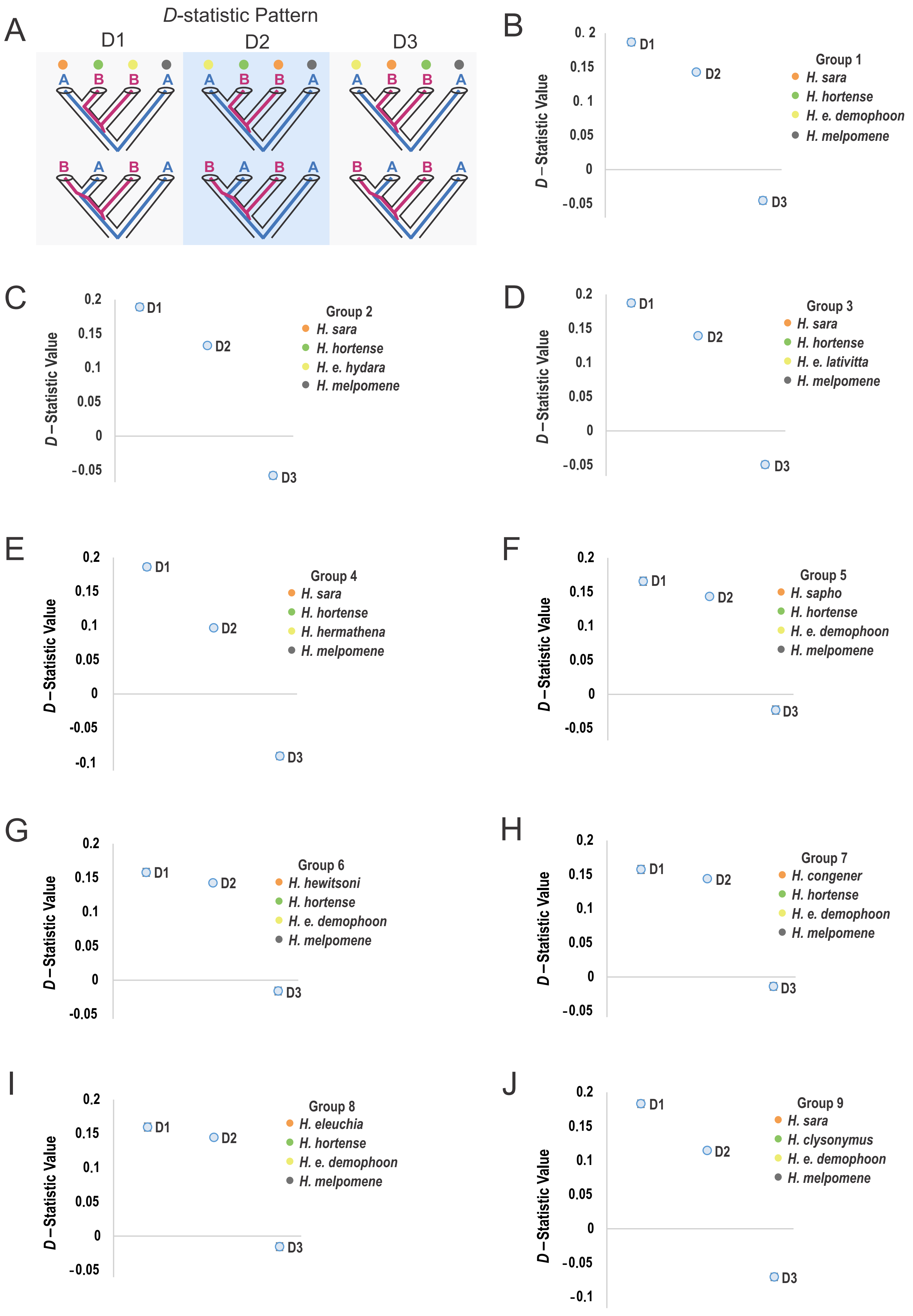
A
D-statistic Pattern
D1
D2
D3
B
D–Statistic Value
Group 1
H. sara
H. hortense
H. e. demophoon
H. melpomene
C
Group 2
H. sara
H. hortense
H. e. hydara
H. melpomene
D
Group 3
H. sara
H. hortense
H. e. lativitta
H. melpomene
E
Group 4
H. sara
H. hortense
H. hermathena
H. melpomene
F
Group 5
H. sapho
H. hortense
H. e. demophoon
H. melpomene
G
Group 6
H. hewitsoni
H. hortense
H. e. demophoon
H. melpomene
H
Group 7
H. congener
H. hortense
H. e. demophoon
H. melpomene
I
Group 8
H. eleuchia
H. hortense
H. e. demophoon
H. melpomene
J
Group 9
H. sara
H. clysonymus
H. e. demophoon
H. melpomene

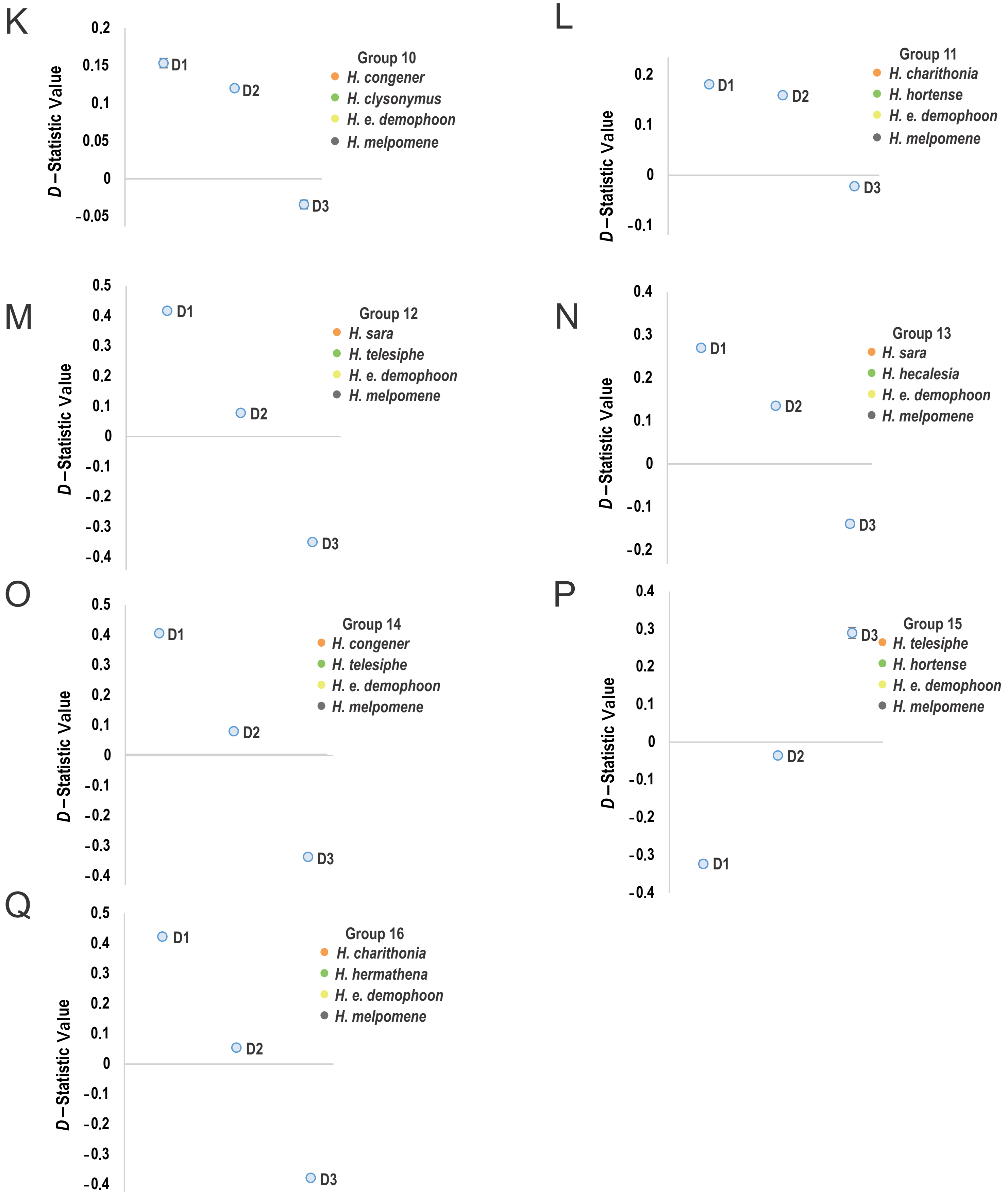

Figure S6. Patterson's *D*-statistic patterns among *Heliconius* comparisons of 16 groups. The topologies and species codes are indicated in panel A. The genome-wide *D*-statistic values are plotted in panel B-Q.

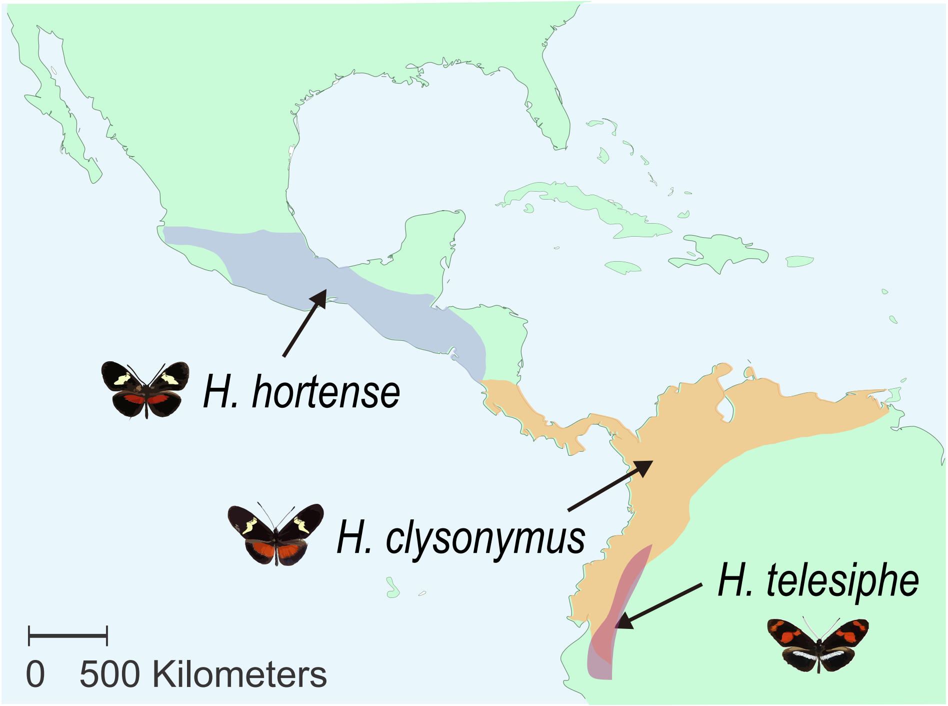

Figure S7. The geographical distribution of *Heliconius* species *H. hortense*, *H. clysonymus* and *H. telesiphe*. The distributions are shown in different colors with corresponding butterfly images of focal species.

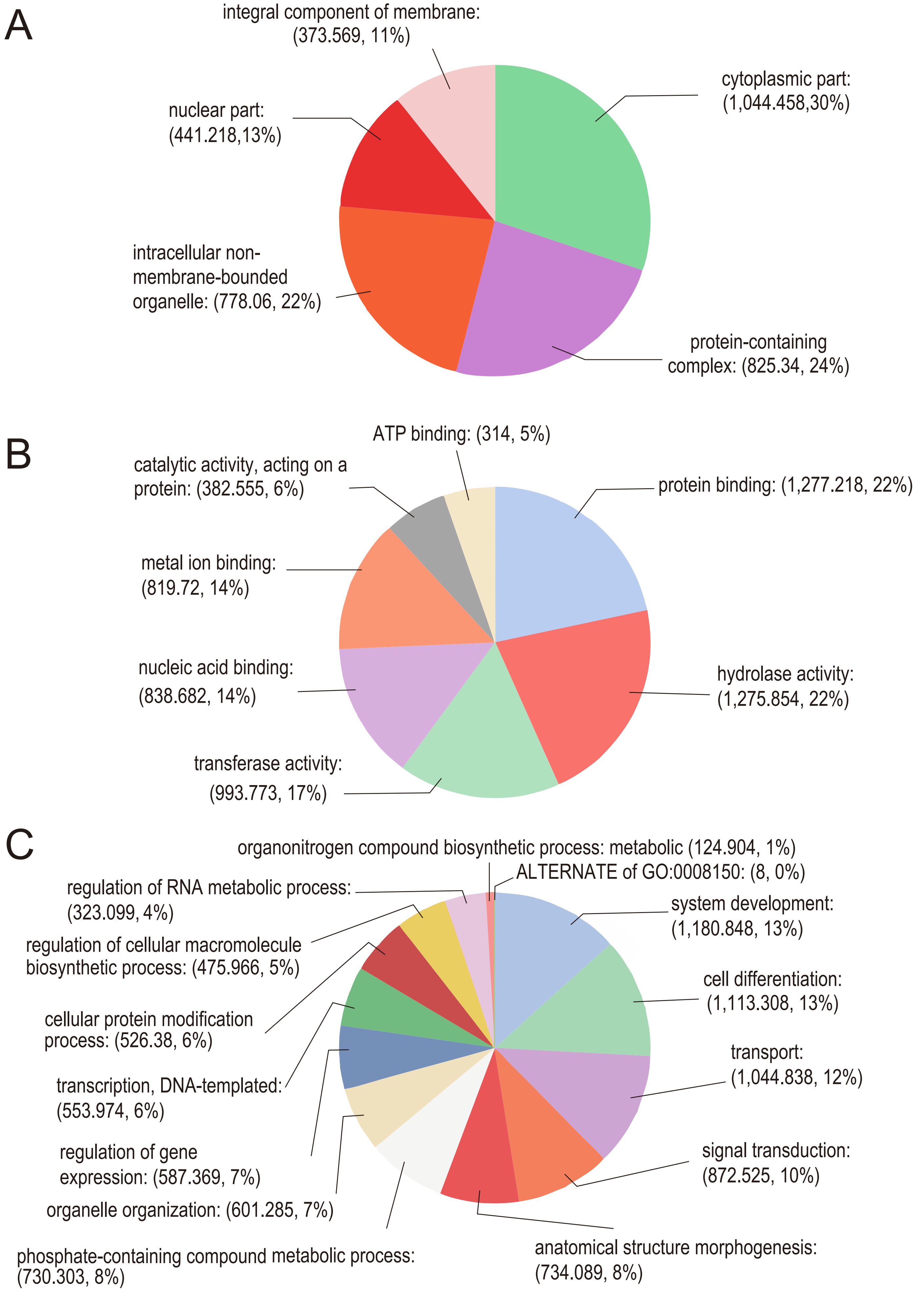

Figure S8. GO distribution of 4042 orthologous clusters. We create multilevel pie charts according to Blast2GO results and show scores and values for group slices in cellular component (A), molecular function (B) and biological process (C).

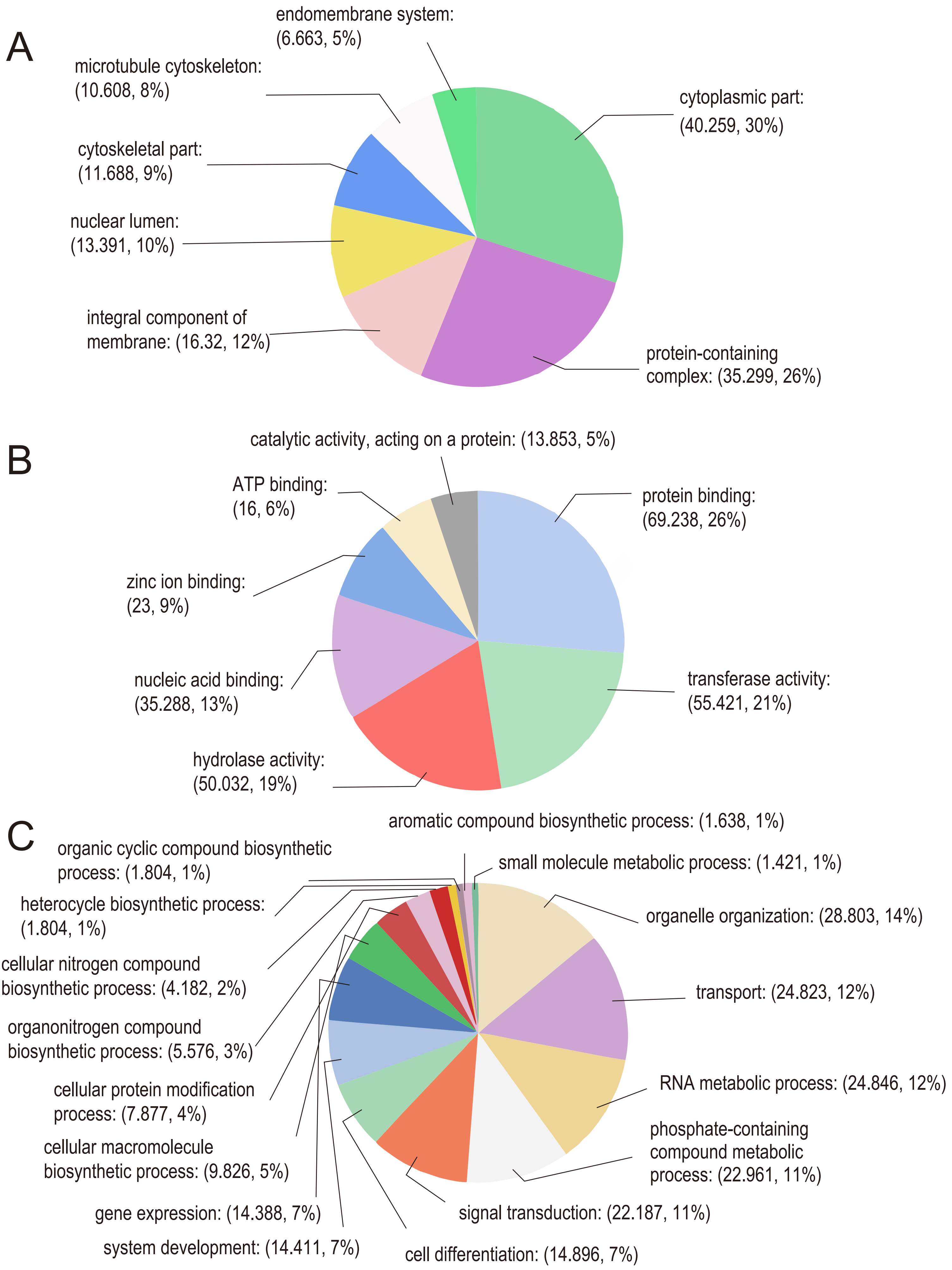

Figure S9. GO distribution of 276 orthologous clusters with Ka/Ks ratios above one. We create multilevel pie charts according to Blast2GO results and show scores and values for group slices in cellular component (A), molecular function (B) and biological process (C).

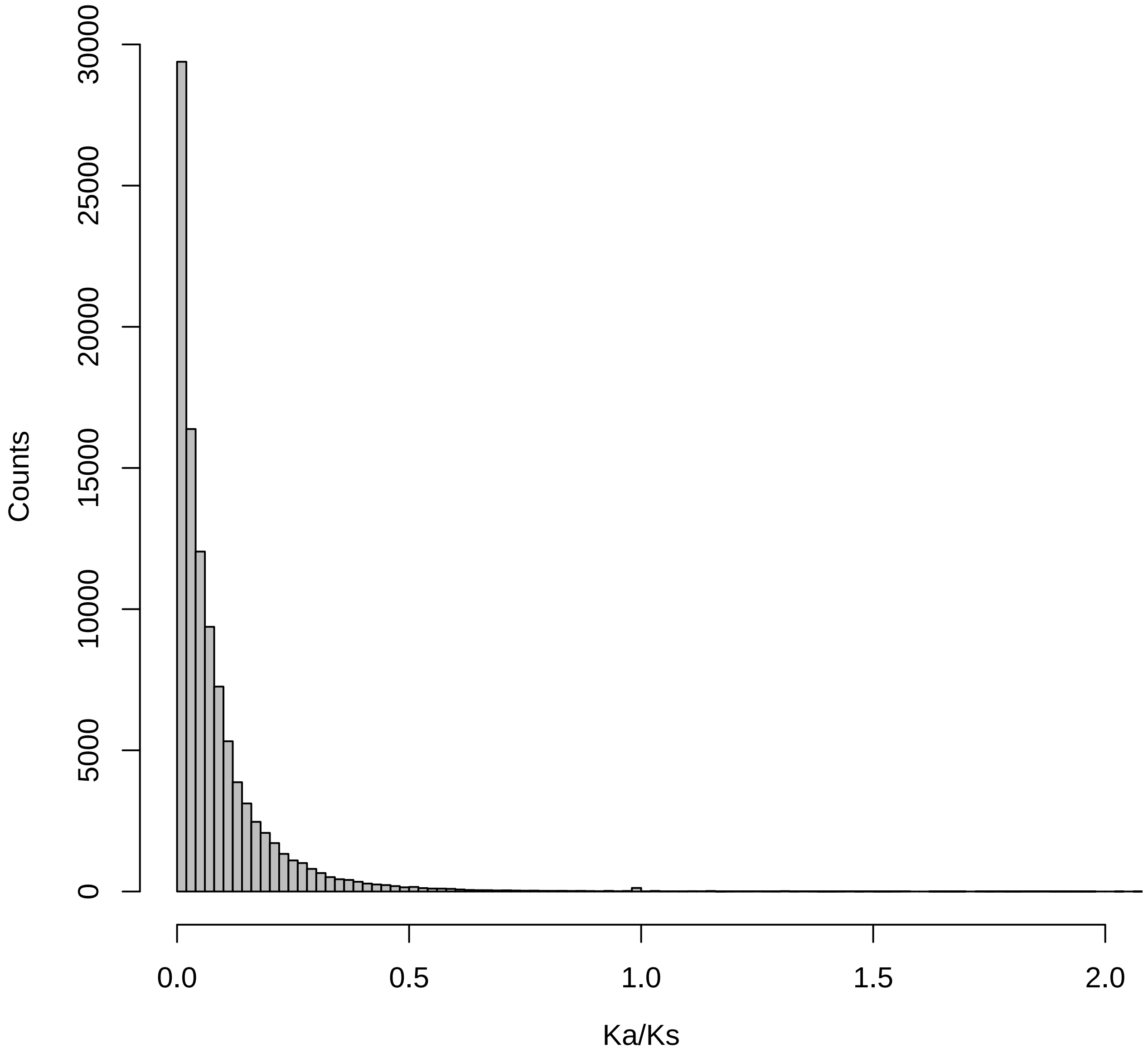

Figure S10. Histogram of Ka/Ks distribution among pairwise comparisons in 4042 orthologous clusters. We plot Ka/Ks ratios for 102,243 comparisons that yield a nonzero Ks value.

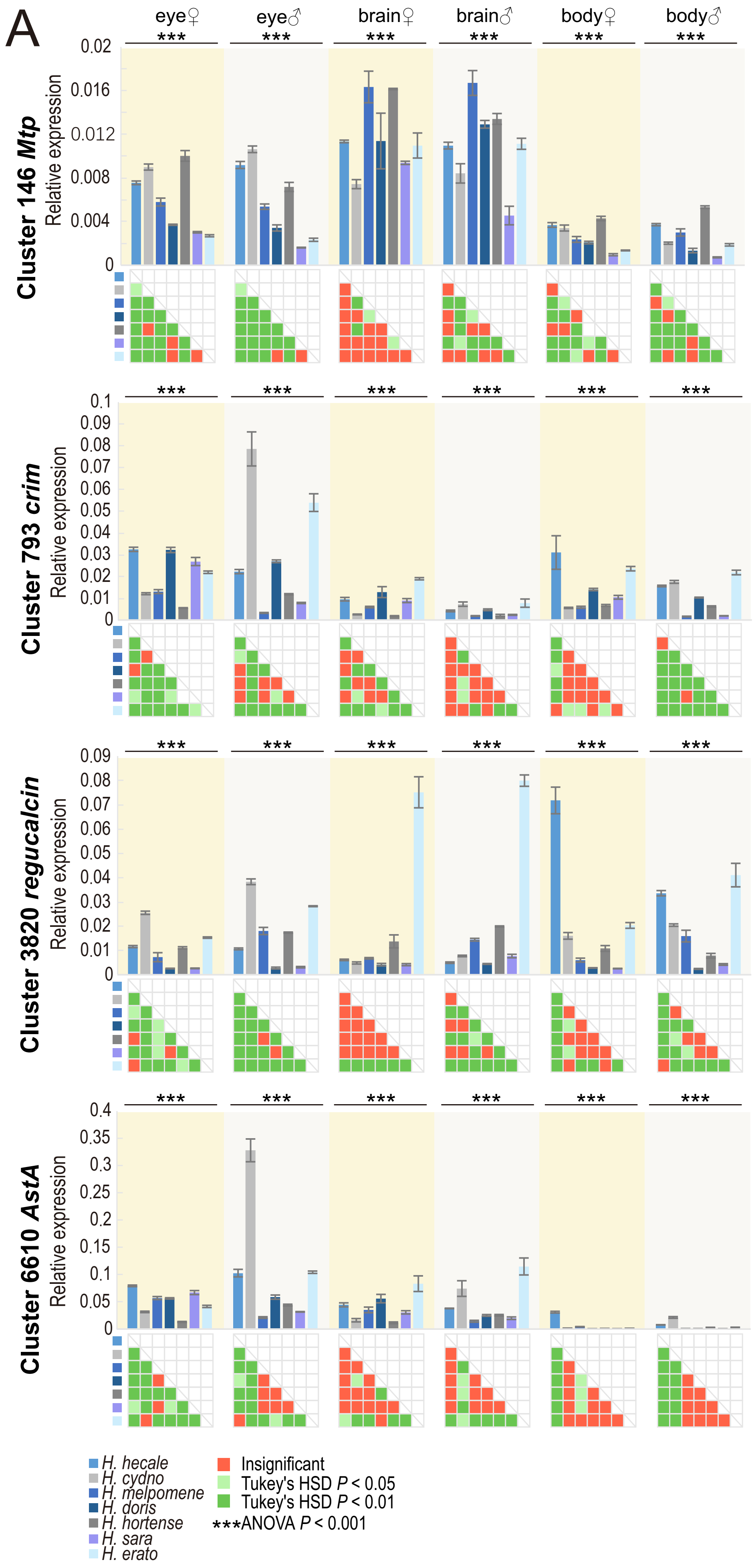
A
eye♀
eye♂
brain♀
brain♂
body♀
body♂
***
Cluster 146 Mtp
Cluster 793 crim
Cluster 3820 regucalcin
Cluster 6610 AstA
Relative expression
H. hecale
H. cydno
H. melpomene
H. doris
H. hortense
H. sara
H. erato
Insignificant
Tukey's HSD P < 0.05
Tukey's HSD P < 0.01
***ANOVA P < 0.001

B

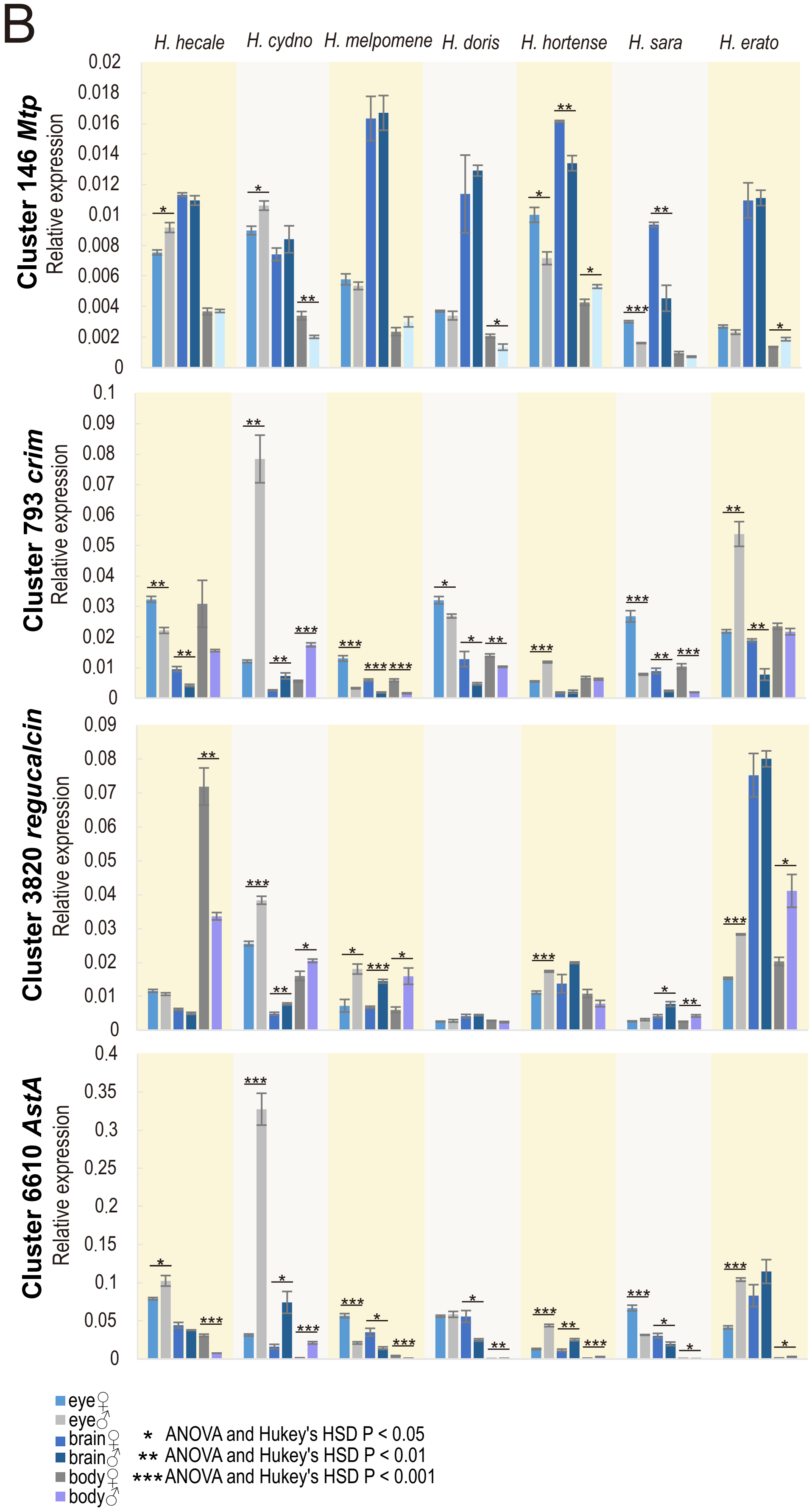

H. hecale
H. cydno
H. melpomene
H. doris
H. hortense
H. sara
H. erato
Cluster 146 Mtp
Relative expression
Cluster 793 crim
Cluster 3820 regucalcin
Cluster 6610 AstA
eye♀
eye♂
brain♀
brain♂
body♀
body♂
* ANOVA and Hukey's HSD P < 0.05
** ANOVA and Hukey's HSD P < 0.01
*** ANOVA and Hukey's HSD P < 0.001

C

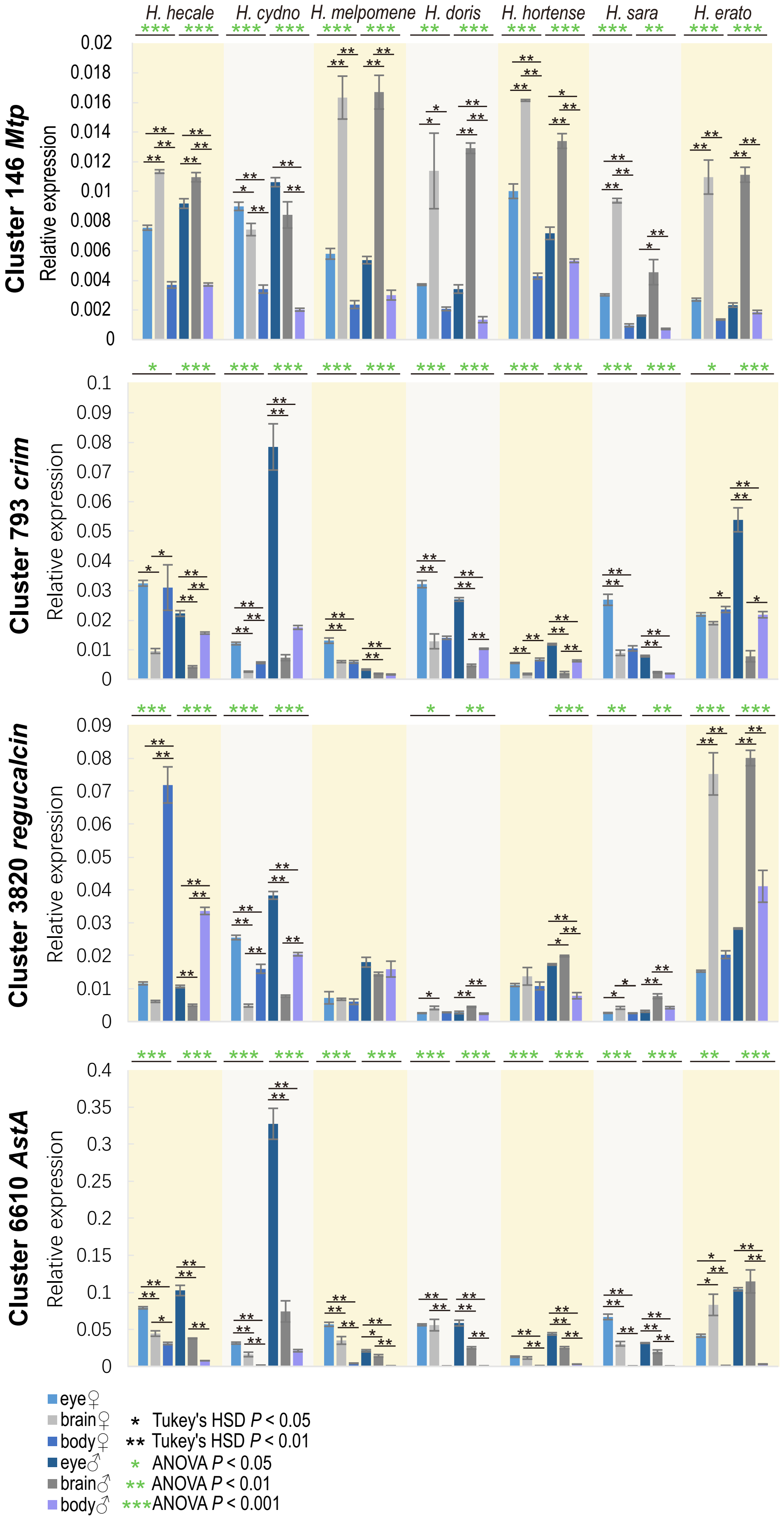

Figure S11. Pairwise comparisons of expression patterns of four target genes. Six gender-specific pairwise comparisons of relative expression patterns in the eye, brain and body tissues are separately compared among seven *Heliconius* species (A), between females and males (B) and among gender-specific eye, brain and body tissues (C) using ANOVA and Tukey's HSD tests.
